# *β*-Hydroxylation of *α*-amino-*β*-hydroxylbutanoyl-glycyluridine catalyzed by a nonheme hydroxylase ensures the maturation of caprazamycin

**DOI:** 10.1038/s42004-022-00703-6

**Published:** 2022-07-28

**Authors:** Saeid Malek Zadeh, Mei-Hua Chen, Zhe-Chong Wang, Elahe K. Astani, I-Wen Lo, Kuan-Hung Lin, Ning-Shian Hsu, Kamal Adhikari, Syue-Yi Lyu, Hsin-Ying Tsai, Yuma Terasawa, Miyuki Yabe, Kazuki Yamamoto, Satoshi Ichikawa, Tsung-Lin Li

**Affiliations:** 1 Genomics Research Center, Academia Sinica, Taipei, Taiwan; 2 Chemical Biology and Molecular Biophysics Program, Taiwan International Graduate Program, Academia Sinica, Taipei, Taiwan; 3 Institute of Bioinformatics and Structural Biology, National Tsing Hua University, Hsinchu, Taiwan; 4 Department of Chemistry, National Taiwan University, Taipei, Taiwan; 5 Department of Chemistry, Faculty of Science, Tarbiat Modares University, Tehran, Iran; 6 Molecular and Biological Agricultural Sciences Program, Taiwan International Graduate Program, Academia Sinica and National Chung Hsing University, Taipei, Taiwan; 7 Graduate School of Pharmaceutical Sciences, Hokkaido University, Sapporo, Japan; 8 Global Station for Biosurfaces and Drug Discovery, Global Institution for Collaborative Research and Education (GI- CoRE), Hokkaido University, Sapporo, Japan; 9 Biotechnology Center, National Chung Hsing University, Taichung City, Taiwan

**Keywords:** Enzyme mechanisms, Oxidoreductases, Biosynthesis, X-ray crystallography

## Abstract

Caprazamycin is a nucleoside antibiotic that inhibits phospho-*N*-acetylmuramyl-pentapeptide translocase (MraY). The biosynthesis of nucleoside antibiotics has been studied but is still far from completion. The present study characterized enzymes Cpz10, Cpz15, Cpz27, Mur17, Mur23 out of caprazamycin/muraymycin biosynthetic gene cluster, particularly the nonheme αKG-dependent enzyme Cpz10. Cpz15 is a β-hydroxylase converting uridine mono-phosphate to uridine 5′ aldehyde, then incorporating with threonine by Mur17 (Cpz14) to form 5′-*C*-glycyluridine. Cpz10 hydroxylates synthetic 11 to 12 in vitro. Major product 13 derived from mutant Δ*cpz10* is phosphorylated by Cpz27. β-Hydroxylation of 11 by Cpz10 permits the maturation of caprazamycin, but decarboxylation of 11 by Mur23 oriented to muraymycin formation. Cpz10 recruits two iron atoms to activate dioxygen with regio-/stereo-specificity and commit electron/charge transfer, respectively. The chemo-physical interrogations should greatly advance our understanding of caprazamycin biosynthesis, which is conducive to pathway/protein engineering for developing more effective nucleoside antibiotics.

## Abstract (web summary)

Caprazamycin is a nucleoside antibiotic that can interrupt bacterial peptidoglycan biosynthesis, but the biosynthesis of caprazamycin is not fully understood yet. Here, the authors characterize five key enzymes from caprazamycin/muraymycin gene cluster, particularly nonheme αKG-dependent enzyme Cpz10 as a β-hydroxylase that recruits two iron atoms and leads to caprazamycin formation.

## Introduction

Nucleoside antibiotics are a group of natural products which specifically inhibit phospho-*N*-acetylmuramyl-pentapeptide translocase (MraY), an essential enzyme involved in the bacterial peptidoglycan biosynthesis^1^. Up to date, no any antibiotics that inhibit MraY have been approved for clinical use, underscoring that MraY and nucleoside antibiotics respectively are drug target and drug candidate in pair as an ideal strategy to conquer antibiotic drug resistance^2^. The pathway elucidation for potent nucleoside antibiotics has been a research hot spot^3^. Two approaches are generally employed in the field, namely, in vivo and in vitro approaches. Both are not exclusive but complementary to each other. Noticeably, collecting and purifying hydrophilic intermediates from given mutants are tedious and challenging^4^. On top of that, no known substrates or substrates unavailability are another difficulty in in-vitro study. These limitations considerably hamper the progress of biosynthetic pathway illustration of natural products. To unravel these shackles, an integrated approach by combining in vivo, in vitro, synthetic chemistry and computational chemistry is emerging that has been proven powerful^5^.

**Fig. 1:**
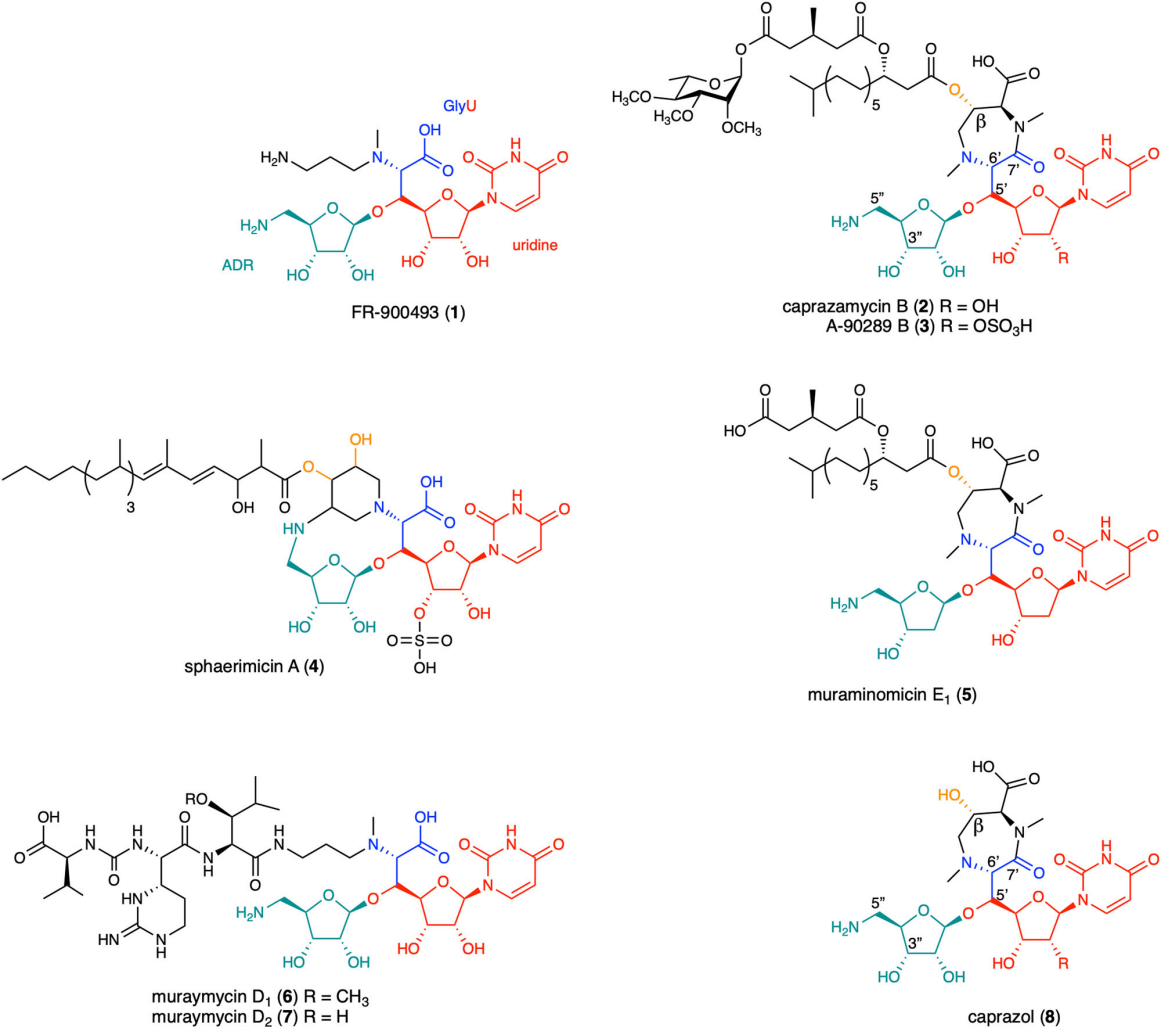
Chemical structures of representative MraY inhibitors. Nucleoside antibiotics are composed of some characteristic moieties: uridine colored red, 5″-amino-5″-deoxyribose (ADR) colored green, the glycine moiety colored blue and the *β*-hydroxyl group colored orange. The numbering system referred to in the text is designated.

Nucleoside antibiotics, such as FR-900493 (**1**), caprazamycin (**2**), liposidomycin (**3**), sphaerimicin (**4**), and muraminomicin (**5**) (Fig. 1), all feature a uridine moiety in addition to an unusual 5′′-amino-5′′-deoxyribose (ADR). In view of structures, nucleoside antibiotics can be subdivided into two groups: the lipid I transition-like subgroup *e.g*. compounds **2**–**5** and the Park’s nucleotide subgroup *e.g*. compounds **6** and **7**. The uridine moiety is coupled with a short-chain amino acid to form an ADR-GlyU (5′-*C*-glycyluridine) core the drug warhead, which can be attached with a 6′-*N*-alkylamine side chain. This alkylamine is hydroxylated at its *β*-position (*β*-OH) for the biosynthesis toward the lipid I transition-like subgroup^6,7^. On the other hand, the decarboxylation of the 6′-*N*-alkylamine side chain is pivotal toward the biosynthesis of the Park’s nucleotide subgroup (see below). FR-900493 (**1**) acts as a rudimentary structure, where the ADR-GlyU disaccharide core is associated with an aminopropyl group and a methyl group at the glycine portion. By contrast, caprazamycin (**2**) includes a characteristic seven-membered diazepanone ring with two methyl groups each at one of two ring-nitrogen atoms (caprazol **8**); this heterocyclic ring is decorated with a long lipid side chain at the *β*-OH, to which 3-methyl glutaric acid and 2,3,4-*O*-methyl-*L*-rhamnose are appended^8–11^.

The addition of the fatty acyl chain to caprazol **8** has been determined in vivo using a gene-deleted mutant (Δ*cpz*23, coding for a lipase-like enzyme)^12^, underlining the importance of the *β*-OH functionality. However, the enzyme that commits the *β*-hydroxylation remains elusive. In view of the biosynthetic gene clusters (BGCs) of lipo-nucleoside antibiotics caprazamycin (**2**) and liposidomycin (A-90289) (**3**), each individual BGC contains two *α*KG-dependent monooxygenases Cpz10,15 and LipG,L^13,14^, the potential candidates. Nonheme iron-dependent enzymes play crucial roles in the diversification of secondary metabolites^15^. This type of enzymes exists across all domains of lives and has been studied extensively, for example, dopamine hydroxylase, deacetoxycephalosporin C synthase (DAOCS) and taurine dioxygenase (TauD)^16–18^. These enzymes utilize a high-spin iron (Fe^II^) to activate dioxygen into a reactive Fe^IV^-oxo species at the expense of one molecule of *α*KG to oxygenate/oxidize/rearrange a wide variety of organic compounds^17,19^. The active site is located within a core *β*-barrel domain in this class of enzymes^16^. Here we report that Cpz15 is the starter enzyme; Cpz10 is the *β*-hydroxylase recruiting an additional auxiliary iron to bring its activity into full play; Cpz27 is a phosphotransferase, alongside biochemical functionalities of Cpz13, 14 and Mur23. Our in vivo and in vitro results suggest that the phosphorylation at the 3″-OH takes place after glycosylation, methylation and alkylation at odds with the previously proposed scenario^4,20^.

## Results

### Cpz10 and 15 are nonheme oxygenases

**Fig. 2:**
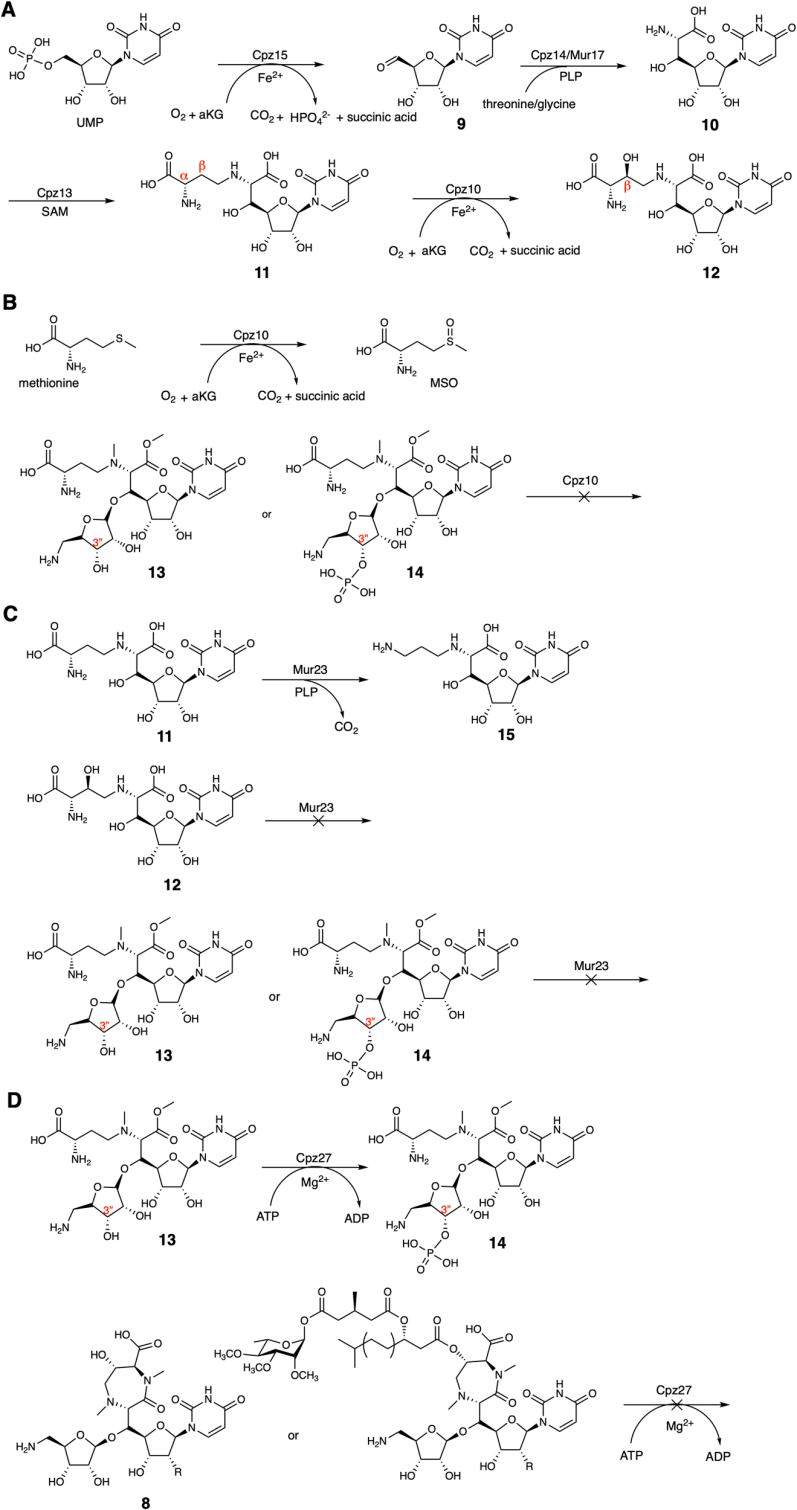
Reaction scheme for selected enzymes referred to in this study. **A** Biosynthetic steps committed by Cpz15, Cpz14/Mur17, Cpz13 and Cpz10 in sequence. **B** Methionine or selenium can be oxygenated by Cpz10; compounds **13** or **14** cannot be accepted by Cpz10. **C** Compound **11** can be decarboxylated by Mur23 to compound **15** as opposed to compound **12** that cannot be consumed. **D** Compound **13** can be phosphorylated by Cpz27 to compound **14**, while compound **8** cannot be utilized.

Two genes, *cpz*10 and *cpz*15, that code for *α*KG dependent enzymes, exhibit in the BGC of caprazamycin. In silico analysis, *cpz*15 is a homolog to the clavaminic acid synthetase (CAS)-like protein, which also appears in the BGCs of liposodomycin, jawsamycin, capuramycin (Supplementary Fig. 1) and muraymycin (**6**, **7**). The one in muraymycin is *mur*16 (Supplementary Fig. 2), while the functions of Cpz15 and Mur16 were annotated differently. Cpz15 is designated as an oxygenase (Supplementary Fig. 3) responsible for the *β*-hydroxylation at a later step in contrast to Mur16 that engages in an oxidative dephosphorylation reaction in the very first step^14^. Rather, Cpz10 was assigned a role as that of Mur16. Recently, Cpz15 is re-proposed as the starter enzyme responsible for the oxidative dephosphorylation reaction^21^. To resolve this controversy, we set off biochemical examination for purified Cpz10 or Cpz15 versus possible substrates, including uridine, uridine monophosphate (UMP), uridine diphosphate (UDP) and uridine triphosphate (UTP). Of them, only did a new peak emerge on the LC trace concomitant with dwindling of UMP in the reactions with Cpz15 (Fig. 2A, and 3A). This new peak was subjected to MS analysis, which showed a mass unit (M + H)^+^ of *m/z* = 243.1 indicating formation of uridine 5′-aldehide (U5’A **9**) (Supplementary Fig. 4)^22^, thereby redefining Cpz15 a nonheme-dependent oxidative de-phosphorylase rather than a *β*-hydroxylase.

**Fig. 3:**
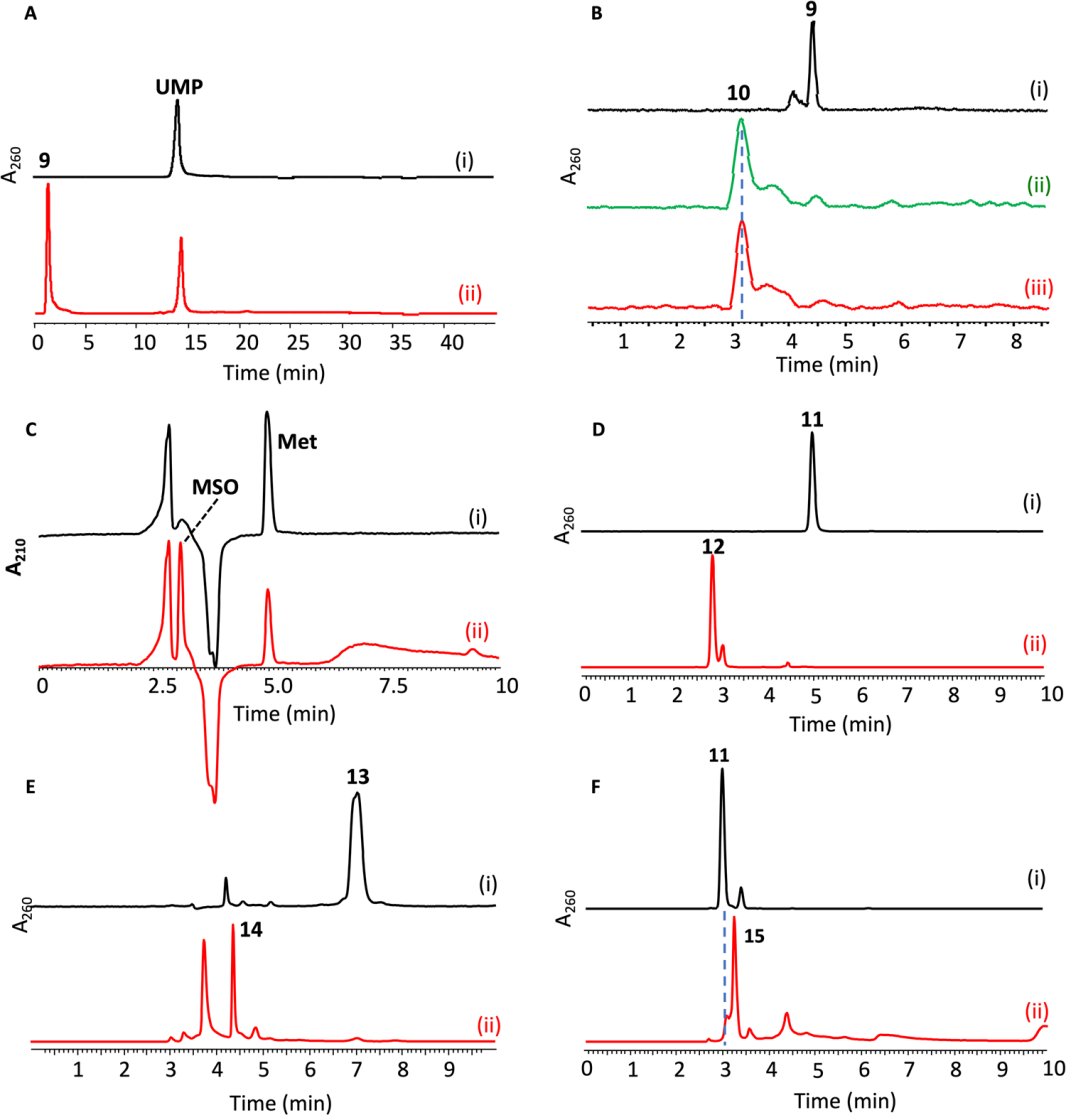
LC traces for enzymatic reactions catalyzed by enzymes involved in the biosynthesis of nucleoside antibiotics. **A** Oxidative dephosphorylation reactions catalyzed by Cpz15, in which UMP (i, without Cpz15) is converted to compound **9** (ii, with Cpz15). **B** Coupling reactions catalyzed by Mur17, in which compound **9** (i, without enzyme) plus either threonine (ii) or glycine (iii) can be converted to compound **10**; both substrate and product were derivatized with dansyl chloride before subjected to LC analysis. **C** Oxygenation reactions catalyzed by Cpz10, in which methionine (i, without Cpz10) can be converted to MSO (ii, with Cpz10). **D** Hydroxylation reactions catalyzed by Cpz10, in which synthetic compound **11** (i, without Cpz10) can be converted to compound **12** (ii, with Cpz10). **E** Phosphorylation reactions catalyzed by Cpz27, in which collected compound **13** (i, without Cpz27) can be converted to compound **14** in the presence of ATP (ii, with Cpz27). **F**. Decarboxylation reactions catalyzed by Mur23, in which compound **11** (i, without Mur23) can be converted to compound **15** (ii, with Mur23); both substrate and product were derivatized with dansyl chloride before subjected to LC analysis.

As shown in Fig. 2A, given that UMP is oxidized by Cpz15 to U5’A (**9**)^22^, compound **9** would then be catalyzed by Cpz14 with threonine to form 5′-*C*-glycyluridine (5’GlyU **10**). To confirm this prediction, we expressed Cpz14 in *E. coli* or Streptomyces, while, unfortunately, it is insoluble in either system. To overcome this problem, we borrowed Mur17 from the muraymycin BGC, an enzyme equivalent to Cpz14 and soluble in *E. coli*. The LC/MS analysis shows formation of compound **10** (expected mass unit at *m/z* = 318.16 (M + H)^+^) for the reactions with compound **9** and threonine plus Mur17 (Figs. 2A and 3B; and Supplementary Fig. 5) in agreement with previous results^6,23^. Interestingly, not only threonine but also glycine can be accepted by Mur17 while assayed against all proteinogenic *L*-amino acids and their *D*-isomers.

### Cpz10 is the *β*-hydroxylase

Since Cpz15 is not the *β*-hydroxylase, the likelihood now befalls Cpz10. In silico analysis, Cpz10 also shows protein sequence similarity/identity analogous to nonheme *α*KG dependent enzymes closest to aspartyl/asparaginyl *β*-hydroxylase (Supplementary Fig. 6). Concerning the reaction timing/order, the *β*-hydroxylation could occur on free amino acids, compound **11** (see below), or the seven-membered heterocyclic ring (*e.g*. compound **8** without *β*-OH). We first assayed Cpz10 against all *L*/*D*-amino acids, dopamine and pipecolic (Supplementary Fig. 7). Interestingly, *L-*methionine and *L-*selenomethionine were found subject to oxygenation, while the oxygenation takes place at the sulfur (MSO)/selenium atom instead of the expected *β*-carbon (*β*C; HR-MS, LC-MS/MS in conjunction with 2D NMR confirms the product is MSO with the (M + H)^+^ ion at *m/z* = 166.05, Figs. 2B and 3C and Supplementary Fig. 8). Next, *S*-adenosylmethionine (SAM) was examined, which was however ruled out as no product found (data not shown). It has been proposed that the four-carbon sidechain is transferred from SAM to compound **10** by Cpz13 to compound **11** (Figs. 2A and 3B, D and Supplementary Fig. 4a). Because compound **9** is highly unstable, the production of compounds **10** and **11** is therefore limited. These two compounds were then chemically synthesized (see Supplementary methods) following previously published methodologies in total synthesis of caprazamycin^14^. With the synthetic compound **11** in hand, Cpz10 were examined, of which a new peak was brought on LC traces (Fig. 2A, and Fig. 3D). HR-MS and LC-MS/MS analysis showed that this new peak has an (M + H)^+^ ion at *m/z* = 435.1357 in line with the predicted molecular weight of the hydroxylated compound **11** (m/z = 435.1358 for C_15_H_22_N_4_O_11_ + H) (see Supplementary Note 1). X-ray crystallography, at long last, confirmed that the peak is ɑ-amino-*β*-hydroxylbutanoyl-glycyluridine (compound **12**) (see below). Because of limited quantity of compound **11**, we took advantage of *L-*methionine (as Cpz10 can oxygenate it to MSO) to estimate the basic kinetics for Cpz10; the Michaelis constant and turnover rate were determined under the pseudo-first order condition: *K*_m_ = 892.1 ± 9 μM and *k*_cat_ = 58.6 ± 0.8 min^−1^, which are comparable to those of typical nonheme monooxygenases (Supplementary Fig. 9).

### Cpz10 X-ray crystal structures reveal two irons

**Fig. 4:**
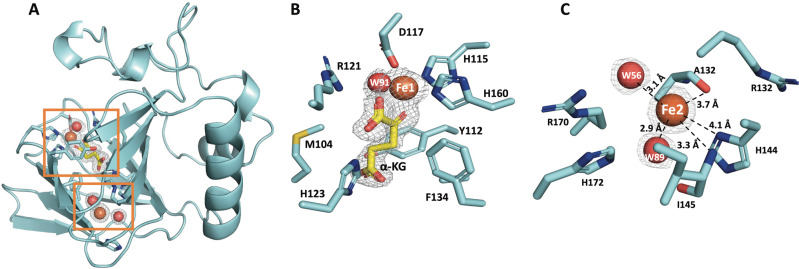
Crystal structure of Cpz10 in complex with irons and *α*-KG. **A** The overall structure of Cpz10, of which the polypeptide chain (colored cyan) folds into a major distorted jelly roll *β*-sheet core and an *α*-helical-loop domain. Two irons and *α*-KG are positioned in the center of the *β*-sheet core. **B** The closed-up view of active site, where the active site iron (Fe1, colored orange) is held by a typical facial triad H115, H160, and D117 (cyan sticks) in association with the *α*-KG bidentate (yellow stick) and a water molecule (W91, colored red) to form a tetragonal bipyramidal coordination geometry. **C** The closed-up view of the second iron (Fe2, colored orange) which is located in the end of the *β*-sheet core. Fe2 is associated intimately with two water molecules (W89 and W56 in 2.9 and 3.1 Å, respectively) as well as with the backbone of R133 and in short distance with residues F134, H144, R170, and H172.

To better understand the catalytic mechanism of Cpz10, we set out to determine crystal structures of Cpz10 in apo and complex forms (Supplementary Fig. 10). The purified Cpz10 was first crystallized and subjected to X-ray diffraction. The phase problem was solved using molecular replacement (MR), whereby the initial model obtained was used as the template for building complex structures. We determined five structures, including one apo structure (PDB code: 7V4N), two mutants (PDB codes: 7V4O and 7V4P) and two complexes (with *α*KG (PDB code: 7V4M), or with succinic acid (SA), compound **12** (PDB code: 7V4F) (see below, and Supplementary Fig. 11B for stereo views and density maps; the data-collection and refinement statistics are summarized in Table 1). Based on gel filtration chromatograph, Cpz10 is a homodimer in an aqueous solution (Supplementary Fig. 12). Consistently, two polypeptide chains are packed in an asymmetric unit (Supplementary Fig. 11) with immense protein-protein interactions at the dimer interface. A single polypeptide chain is folded in a twisted *β*-sheet jelly roll (cupin) domain, a common fold for a typical *α*KG-dependent nonheme enzyme^24^. Unexpectedly, two iron metals were identified intrinsically existing in all structures determined except the apo form. The ferric iron, labeled Fe1, is held by an archetypal metal-chelating triad (His115, His160, Asp117), *α*KG that coordinates to Fe1 in a bidentate manner, and a water molecule (Wt91) that takes the sixth coordinate altogether to form a tetragonal bipyramidal geometry (Fig. 4)^17,25,26^. This 6-coordinate complex is akin to most reported complexes of *α*KG-dependent enzymes, where the carboxyl group of *α*KG coordinates *trans* to the distal histidine. As to the second iron, labeled Fe2, it is associated with two water molecules (Wt56 and Wt89) in close proximity to residues Arg170, Arg133, His144, Thr128, and Ile145 but away from Fe1 by 12 Å (Fig. 4 and Supplementary Fig. 13).

**Table 1:** Data collection and refinement statistics (molecular replacement).

	7V4N	7V4M	7V4F	7V4O	7V4P
*Data collection*					
Space group	P 4_3_ 2_1_ 2	C 2 2 21	P 4_3_ 2_1_ 2	C 1 2 1	P 4_3_ 2_1_ 2
Cell dimensions					
*a*, *b*, *c* (Å)	82.2, 82.2, 104.2	75.18, 77.56, 68.11	82.04, 82.04, 103.89	150.83, 39.03, 76.59	82.04, 82.04, 103.89
α, β, γ (°)	90, 90, 90	90, 90, 90	90, 90, 90	90, 119.33, 76.59	90, 90, 90
Resolution (Å)	29.83–2.20 (2.28–2.20)	19.86–1.90 (1.97–1.90)	26.48–1.97 (2.04–1.97)	25.03–1.65 (1.71–1.65)	26.46–1.94 (2.02–1.95)
*R*_sym_ or *R*_merge_	0.078 (0.79)	0.039 (0.501)	0.040 (0.570)	0.051 (0.141)	0.066 (0.509)
*I*/σ*I*	24.9 (3.4)	23.6 (2.4)	33.5 (3.8)	32.3 (13.1)	36.9 (4.2)
Completeness (%)	100.0 (100.0)	100.0 (100.0)	96.2 (98.1)	99.0 (100.0)	100.0 (100.0)
Redundancy	14.4 (14.7)	5.3 (5.4)	9.8 (9.9)	7.0 (7.9)	15.7 (13.2)
*Refinement*					
Resolution (Å)	2.2	1.9	1.98	1.65	1.95
No. reflections	17812	15444	25285	46715	26621
*R*_work_/*R*_free_	0.1864/0.2393	0.1865/0.2331	0.1911/0.2510	0.1803/0.2138	0.1877/0.2413
No. atoms					
Protein	1403/1406	1374	1400/1390	1447/1447	1404/1399
Ligand/ion	2	18	45	17/17	2
Water	44	135	171	421	112
*B*-factors					
Protein	30.14/36.74	21.73	24.36/32.23	16.77/13.93	23.24/23.41
Ligand/ion	49.99	28.3	26.93	25.125	37.14
Water	25.97	32.41	30.75	27.21	20.29
R.m.s. deviations					
Bond lengths (Å)	0.0085	0.007	0.008	0.006	0.011
Bond angles (°)	1.3695	0.919	1.13	1.03	1.27

**Fig. 5:**
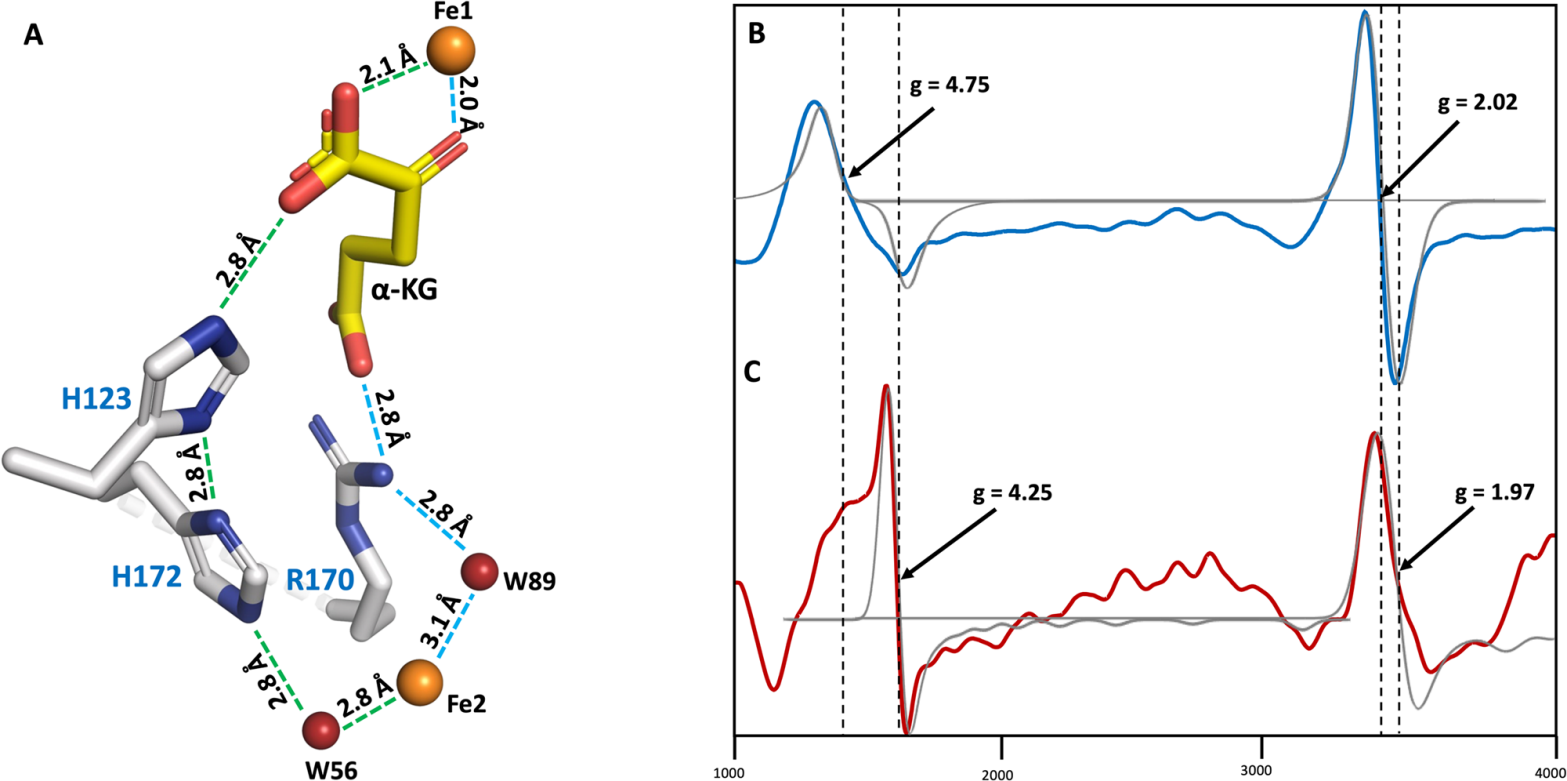
Schematic representation of the CT Path in Cpz10 and EPR spectra. **A** The hidden CT conduit is schematically presented, where CT routes are proposed from Fe2 en route pass points of Wt89, His172, His123, and *α*KG to Fe1 or alternatively through Arg170, *α*KG to Fe1. **B** EPR spectrum (black line) for an enzymatic reaction of Cpz10 in a 50 mM potassium phosphate buffer solution at pH 7.5 containing 0.2 mM *α*KG and 0.2 mM ascorbic acid under an aerobic condition; simulated EPR spectrum (blue line). **C** EPR spectrum (black line) for an enzymatic reaction of Cpz10 in a 50 mM potassium phosphate buffer solution containing 0.2 mM *α*KG, 0.2 mM ascorbic acid, and 0.2 mM methionine at pH 7.5 under an aerobic condition; simulated EPR spectrum (red line).

Based on sequence alignment, the metal-chelating residues His115, Asp117, and His160 are highly conserved as an iron-binding motif HRDX_n_H in the nonheme oxygenase superfamily; Arg170, another conserved residue, recognizes and interacts with *α*KG through electrostatic forces. Interestingly, a cluster of aromatic or soft atom-containing residues, His172, His144, Thr128, His123, Tyr112, and Met104, are identified to form a cryptic electron/charge-transfer tunnel in a manner similar to that observed in Rieske-type oxygenases (Supplementary Fig. 14a, b)^27–29^: residues His172 and His123 (2.8 Å) take a unique geographic position in a two-hand holding shape in close proximity to Fe2 akin to the one reported in *L*-proline *cis*-4-hydroxylase (PDB CODE: 4P7X) (Supplementary Fig. 15)^16^; residues Thr128 and His144 are on par with residues His172 and His123 in a position to interact with Fe2. Fe1 that is held firmly by chelating residues in a short and strong coordinate covalent bond manner (2.0–2.2 Å); in contrast, Fe2 is held in a relatively loose way by nearby residues (3–4 Å) alongside a satellite water molecule (Wt56, 2.9 Å). These two irons are spatially and temporarily associated for electron/charge transfer by an electron/charge relay system made of a constellation of well-cooperative network Wt89, His172, His123, and *α*KG toward Fe1 (see below, and Fig. 5A, Supplementary Movie 1, Supplementary Movie 2), where the distances between each set of consecutive relay stations are <2.8 Å. To confirm the importance of residues His172 and His123 in this electron/charge transfer system, we performed site-directed mutagenesis (H172A and H123A). Both mutants, somehow, are insoluble, implying that they may assume a critical structural role in maintaining the integrity of the tunnel in addition to the proposed electron/charge relay station.

**Fig. 6:**
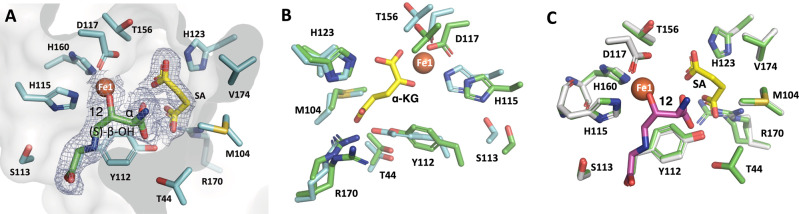
Crystal structure of Cpz10 in complex with Fe1 and *α*-KG and 2-amino-3-hydroxybutanoic acid. **A** Closed-up view of the Cpz10 active site, where the active site Fe1 (colored orange) is coordinated with H106, H154, D108, succinic acid (SA) and 2-amino-3-hydroxybutanoic acid moiety of compound **12** (colored green). *α*KG has undergone decarboxylation to form SA (colored yellow); compound **11** that replaces water (W91; Fig. 4B) at the sixth coordinate has been converted to compound **12**, where the newly introduced OH group poses an *S*-configuration at *β*C in agreement with expected product. **B** Closed-up active-site view of Cpz10 apo (colored green) superimposed over that in complex with *α*-KG (colored yellow) with a r.m.s.d of 0.54, indicating Cpz10 undergoing some degree of conformational change. **C** Closed-up active-site view of Cpz10 apo (colored green) superimposed over that in complex with compound **12** (colored magenta) and SA (colored yellow) with a r.m.s.d of 0.1, indicating Cpz10 undergoing less extent of conformational change after formation of compound **12** and SA. 2F_o_-F_c_ electron-density maps are contoured at 1σ (Supplementary Fig. 11 for stereo views).

To determine the regioselectivity and stereoselectivity reactions catalyzed by Cpz10, we were prompted to pursue the quaternary structure of Cpz10 (in complex with Fe-*α*KG and compound **11**). The complex that finally obtained shows that *α*KG soaked in the crystals has been converted to succinic acid likely undergone an oxidative decarboxylation reaction. Likewise, the soaked compound **11** has been converted to compound **12**, where a new hydroxyl group (*β*-OH) is installed at *β*C of the 2-amino-butanoic acid moiety in *S*-configuration (compound **12**) despite that the ADR-GlyU moiety cannot be flawlessly built because of lack of well-defined electron density as a result of exposed to bulk solvent (Fig. 6 and Supplementary Figs. 11 and 16). Based on the quaternary complex, the overall catalytic mechanism of Cpz10 is proposed as follows: *α*KG is ushered into the active site in association with Fe1 (Fe^III^) to form a high-spin iron-centered tetragonal bipyramidal complex with a water molecule atop the tetragonal plane. Fe1 is then reduced to Fe^II^ by Fe2 through the internal electron/charge relay system identified herein. When compound **11** is placed at its binding site, O_2_ supersedes H_2_O to form a Fe^II^-superoxide anion (or a Fe^III^-superoxide). The superoxide anion subsequently attacks the carbonyl carbon electrophile of *α*KG, resulting in formation of succinic acid (oxidative decarboxylation) and the reactive Fe^IV^-oxo species (by heterolysis). Fe^IV^-oxo then abstracts the nearby *pro*-*S* H-atom from the *β*C of compound **11** in situ to form Fe^III^-OH and the corresponding compound **11** radical. Compound **12** is instantly formed following the rebound recombination reaction between the compound **11** radical and the iron-bound hydroxyl radical (Fig. 6)^30^. As to the sulfoxide formation in *L*-methionine, it can be attributed to this mimetic substrate adapting an unnatural conformation as well as the preference for the soft sulfur base and soft Fe^III^-OH acid pair (Fig. 6 and Supplementary Fig. 11)^17,30–32^.

Nineteen mutants (primers/functions list, Supplementary Table 1A, B) of key residues referred above were made and assayed, most of which lose their enzymatic activity, indicating the importance of their roles proposed. Structurally, Tyr112 exhibits two different conformations in both apo/mutant and ternary complexes. The distances of the phenolic oxygen to the nonheme iron (Fe1) differs in 7.13 Å and 6.32 Å, where the variation is analogous to its counterparts in isopenicillin N synthase or TauD, and is correlated with appearance or disappearance of *α*KG, succinic acid or n-oxalylglycine in the active site^33,34^. Tyr112, however, seems to adapt a fixed conformation to Fe1 in the apo and mutant (R107A) structures (Supplementary Fig. 17). Two mutant structures R170A (at a resolution of 1.65 Å) and D117G (at a resolution of 1.95 Å) were determined: In the former, both irons are absent, *α*KG is no longer present, and Tyr112 takes a fixed conformation close to His160 (2.9 Å) (Supplementary Fig. 17). In the latter, the overall structure is similar to that of apo but retaining Fe2 in contrast to apo or R170A that contains no Fe2 (Supplementary Fig. 18), suggesting there is a dynamic interplay between Arg170 and Fe2. It is worth noting that Fe1 is held tightly by the facial triad with short dative bonds (~2.2 Å), while Fe2 with two satellite water (Wt56 and Wt89) is less confined at a fixed position (Supplementary Fig. 16). This pair of irons may be spatially connected via a hidden electron/charge transfer conduit en route pass points of Wt56, His172, His123, and *α*KG to Fe1 or alternatively via Arg170, *α*KG to Fe1 (Fig. 5A) as a relay system reminiscent to that in Rieske-type oxygenases (the distances between the pass points are <2.8 Å in contrast to >3 Å of those in the apo form, where Fe2 is missing)^27–29^.

### EPR for Cpz10

To probe the oxidation/spin states of the two irons in Cpz10, the solid-state protein (freezing the sample in liquid nitrogen) at defined conditions was examined using electron paramagnetic resonance (EPR) spectroscopy at 77 K. The EPR spectra display two signals, which is correlated to the two irons in Cpz10. These two signals reflect the states in the presence of *α*KG (Fig. 5B) or in the same condition with additional ascorbic acid and *L*-methionine (Fig. 5C). In the former, the EPR spectrum shows a strong S = 1/2 low-spin Fe^III^ signal at g-value 2.02 and a relatively weak S = 5/2 high-spin Fe^III^ signal at g-value 4.75 (Fig. 5B). The presence of an axial low-spin signal indicates spin relaxation of ferromagnetic Fe^III (^^35–37^^)^ as a result of magnetic exchange, accounting for a less constrained metal ion^24,37,38^. In contrast, the high-spin signal comparable to a typical nonheme Fe^III^ is relatively broader and less intensive with a g-value shifted from 4.25 to 4.75 likely due to nonheme iron electronic reconfiguration as well as coordination dynamics^39^. This signal is relatively symmetric suggesting OH^−^ ions in association to the fifth and/or sixth coordinates of the nonheme ferric iron^40^. In the latter, the reduced ferrous iron (Fe^II^) is usually insensitive to EPR (S = 0 or S = 2), while the appearance of a relatively weak signal indicates a portion of Fe^II^ have oxidized and moved to a different spatial position^35,39,41,42^. Given that the Fe^III^ nonheme signal (S = 3/2) moved back to the typical g-value at 4.25^43^ together with the low spin signal dwindled and shifted to 1.97, these phenomena suggest reactions are taking place and proteins are undergoing conformational changes in consistence with the reaction in the presence of substrates and reducing agents. Along with the relative distance change between the two irons (12–8 Å) and the overall protein conformational changes of Cpz10 with/without substrates (superimposition of structures with r.m.s.d. 0.54), a metal-coupled electron transfer (MCET) mechanism was proposed here, whereby electrons are transferred from the less-constrained ferrous iron to the nonheme ferric iron with assistance of an aromatic residues-relay system down the path (Supplementary movie, Fig. 5A). This reasoning is coherent with the EPR analysis where the peak shape is twisted and g-value is changed to g = 1.97 (Fig. 5C)^27,44–47^. This spectrum is similar to the EPR spectra reported for proteins with an antiferromagnetically coupled Fe_2_ (II/III) cluster that displays a broad axial signal with a g-value <2 (S _total_ = 1/2)^48,49^. Moreover, minor peaks between the two major peaks are visible as shown in Fig. 5C but undefinable, which should be attributed to formation of intermediate iron adducts such as ferric oxide, ferric hydroxy and paramagnetic ferric iron-substrate or product complexes^37,50^.

### Investigation of the nature of Fe^III^ and Fe^II^ EPR signals by NBO analysis

To better understand the charge/spin dynamics of active-site nonheme Fe^III^/Fe^II^ during the reaction, we drew on the natural bond orbital (NBO) analysis. Fe^III^ in both intermediate-spin (IS) and high-spin (HS) states has the same natural electron configuration (NEC) as [core] 4 s^0.20^ 3d^6.33^ 4p^0.40^ 4d^0.02^ 5p^0.01^, in which the first three orbitals are considered the valance orbitals containing a total of 6.93 electrons as opposed to the last two orbitals that are Rydberg orbitals with 0.03 electrons. The 6.33 valance electrons occupy the 3d orbitals as follows: d_xy_^1.437^ d_xz_^1.230^ d_yz_^1.360^ ${{{{{{\rm{d}}}}}}_{{{{{{{\rm{x}}}}}}^{2}}-{{{{{\rm{y}}}}}}^{2}}}^{1.243}$ ${{{{{{\rm{d}}}}}}_{{{{{{\rm{z}}}}}}^{2}}}^{1.058}$. Furthermore, the natural charge of the Fe^III^ in both spin states is equal to +1.069 e. Since the NEC of free Fe^III^ cation is designated as [core] 4s 3d^5^ 4p with the natural charge +3.0 e, the increase of valance orbital occupancies and the significant change in the natural charge of the Fe^III^ cation bound to Cpz10 are ascribed to charge transfer (CT) interactions from six ligands, in which the 2 s and 2p orbitals of oxygens and nitrogens of *α*KG, Wt91, His115, Asp117, and His160 coordinate with the 4 s, 3d, and 4p orbitals (Supplementary Fig. 19).

During CT interactions, hybridization of valance orbitals on each cited atom is taken place in order to form the lone pair (LP) hybrid orbitals on either oxygen or nitrogen ligands as well as the lone pair* (LP*) hybrid orbitals on the Fe^III^, including LP*(6), LP*(7), LP*(8), and LP*(9). In all of these CT interactions, electrons are donated from the LP hybrid orbitals of either the oxygen or the nitrogen ligands into nearly empty LP* hybrid orbitals of the Fe^III^. NBO analysis indicates that not only the LP → LP* interaction types identified in HS state are totally common to those characterized in IS state, but also the local orbital types of partner atoms in each of these interactions as well as their hybridization types and electronic occupancies are the same in both spin states (Supplementary Table 2). The energy of LP → LP* interaction, called the second-order stabilization energy, E^(2)^, is evaluated by the second-order perturbation theory according to the Eq. (1) below:

1 $${E}^{(2)}=\Delta {E}_{CT}=\Delta E({n}_{i}\to {n}_{j}^{\ast })=-2\frac{{\langle {n}_{i}|F|{n}_{j}^{\ast }\rangle }^{2}}{(\varepsilon ({n}_{j}^{\ast })-\varepsilon ({n}_{i}))}$$

$\langle {n}_{i}|F|{n}_{j}^{\ast }\rangle$ is the Fock matrix element, while the denominator is the energy difference between the donor (LP) and the acceptor (LP*) orbitals.

It is evident from the results shown in Supplementary Table 2 that the second-order stabilization energy, E^(2)^ (refs. ^51–53^), of a given CT interaction in IS state is only slightly different from that in HS state. On the other hand, the amount of the transferred charge, qCT(refs. ^52^), between the interacting local orbitals in each common CT interaction of these two spin states are nearly the same (or the same). The stabilization energy of the LP → LP* interaction is an appropriate criterion to evaluate the magnitude of the interaction strength between the LP* of Fe^III^ and the LP of its partner atom, approximately equal to E^(2)^ values of these common CT interactions signifying their almost identical strengths in both spin states. Therefore, on the basis of NBO results, we conclude that the nature of the weak EPR signal detected at the g value of 4.75 g (Fig. 5B) can be derived from both the intermediate-spin state (S = 3/2) and the high-spin state (S = 5/2) of the Fe^III^ bound to the Cpz10 active site.

NBO analysis shows that LP (1) orbitals on Nε2 atoms in His115 and His160 overlap with LP*(9) and LP*(8) of Fe^III^, respectively. The result of these orbital overlaps is the formation of two bonds of Nε2–Fe type in pairs of His115–Fe and His160–Fe (Supplementary Fig. 19). As there is only a slight difference between E^(2)^ values of these CT interactions, the interaction strengths of the Fe^III^ with these two His are the same (Supplementary Table 2). As displayed in Supplementary Fig. 19, the Fe^III^ forms four bonds of O–Fe kind of with the oxygens of Asp117, *α*KG, and Wt91. In all cases, electrons of both LP (1) and LP (2) orbitals on oxygens are transferred to the LP* orbitals on the Fe^III^. As can be seen from Supplementary Table 2, the interactions between the LP (2) and LP* orbitals are much stronger than those between the LP (1) and LP* orbitals because E^(2)^ values of the LP (2) → LP* interactions are considerably greater than those of the LP (1) → LP* interactions. Among them, the LP (2) O4 → LP* (7) Fe^III^ interaction in *α*KG–Fe pair is the strongest CT interaction because it is having the highest values of E^(2)^ (115.90 kJ mol^−1^) and qCT (0.1116 e) compared to other interactions. In addition to O4, LP* (6) and LP* (8) of the Fe^III^ also accept electrons from LP (1) and LP (2) orbitals of its O2 atom. Of these, the LP (2) O2 → LP* (6) Fe^III^ with an E^(2)^ of 73.81 kJ mol^−1^ and a qCT of 0.0624 e is the strongest CT interaction compared to other interactions of O2–Fe. Moreover, the LP (2) Oδ2 → LP* (6) Fe^III^ in Asp117–Fe pair is its second strong interaction out of the surrounding atoms (Supplementary Table 2). Indeed, these three aforementioned CT interactions play essential roles in electron transfer process within Cpz10. In the case of interaction Fe^III^ with Wt91, the strongest CT belongs to the LP (2) O → LP* (9) Fe^III^ interaction with an E^(2)^ of 33.14 kJ mol^−1^ and a qCT of 0.0273 e.

In order to emerge the EPR signal from the Fe^II^ cation, the number of unpaired electron(s) in its 3d orbitals in different spin states must be odd. As known, the NEC of a free Fe^II^ cation is as [core] 4 s 3d^6^ 4p. Therefore, the free ferrous iron lacks unpaired electron in its 3d orbitals in the low-spin state (S = 0), whereas these orbitals possess two and four unpaired electrons in the intermediate-spin state (S = 1) and the high-spin state (S = 2), respectively. It is hence expected that the free Fe^II^ is unable to generate the EPR signal. Nevertheless, the appearance of the EPR signal at g value of 2.02 signifies the presence of Fe^II^ as a ferrous iron. NBO analysis of the Fe^II^–binding site within Cpz10 demonstrates that the Fe^II^ cation has different NECs in three states of LS ([core] 4 S^0.26^ 3d^6.99^ 4p^0.13^ 5S^0.01^ 4d^0.01^ 5p^0.02^), IS ([core] 4S^0.30^ 3d^6.99^ 4p^0.13^ 5S^0.01^ 5p^0.02^), and HS ([core] 4S^1.30^ 3d^6.05^ 4p^0.13^ 4d^0.01^ 5p^0.03^). As a result, due to the participation of Fe^II^ in CT interactions with surrounding atoms (Supplementary Fig. 20), it has an extra unpaired electron in its 3d orbitals in both LS and IS states, while the accepted electrons are mainly transferred into its 4S orbital in HS state. As a consequence, this extra unpaired electron in the 3d orbitals of Fe^II^, which is observed in both LS and IS states, is the reason of the appearance of the EPR signal at 2.02 g. Moreover, due to the transfer of electrons from hybrid orbitals of the neighboring atoms into LP and LP* hybrid orbitals, NBO charges on the Fe^II^ cation in LS and IS states are 0.586 e and 0.542 e, respectively.

In both spin states, NBO analysis detects three CT interactions of the LP O → LP* Fe type in pairs of Ala132–Fe, Wt56–Fe, and Wt89–Fe as well as the LP Cδ1 → LP* Fe interaction in Ile145–Fe pair (Supplementary Table 3). Among them, the LP (2) O Wt89 → LP* (4) Fe^II^ interaction with an E^(2)^ of 13.22 kJ mol^−1^ and a qCT of 0.0302 e is the strongest CT interaction inside the Fe^II^–binding site of Cpz10. The LP (1) O Ala132 → LP* (6) Fe^II^ is the second strong CT interaction of Fe^II^. Thus, these two interactions play important roles in the electron transfer process within the Fe^II^–binding site. It is evident (Supplementary Data 3) that the Fe^III^ not only involves in much more CT interactions than the Fe^II^, but also it interacts more strongly with its neighboring atoms within the Cpz10 active site.

### In vivo study using the *cpz*10-deletion mutant

To validate the function of Cpz10 in vivo, an in-frame *cpz*10-deletion mutant was prepared. The 34.7 kb *cpz* biosynthetic gene cluster (BGC) was cloned into pMKBAC02 to give pMKBAC02-CPZ-Int (Supplementary Fig. 21). In vitro CRISPR/Cas9 was performed to edit pMKBAC02- CPZ-Int to overcome the high GC content of *Streptomyces albus* J1074::ErmE*-crpsc^54^. Both pMKBAC02- CPZ-Int and pMKBAC02-Cpz-Int∆*cpz*10 were individually integrated into the genome of *Streptomyces albus* J1074 for heterologous expression of Cpz or Cpz∆*cpz*10 (Supplementary Fig. 22)^55^. Liquid chromatography mass spectroscopy (LC–MS) analysis identified three Cpz aglycones respectively with molecular weights at *m/z* 930, 944, and 958 in consistence with previous reports (Supplementary Fig. 23)^14^. The caprazamycin production was totally abolished in the ∆*cpz10* mutant in contrast to that in Cpz-containing species where caprazamycin is produced. ∆*cpz10* was cultured in a scale of 30 litters, from which only was one compound (2 mg) purified (Supplementary Scheme 1) (Supplementary Fig. 24 and Supplementary Note 1)^4,12,14,20,56^. This compound was subjected to MS and NMR analysis, confirming that it is an ADR-GlyU disaccharide species (compound **13**, where both C6′ amine and C7’ carboxyl groups are methylated) instead of the expected compound **11**. The chemical structure resolved is unexpected, while it is very informative pertaining to the cyclization of the seven-membered diazepanone ring. Our reasoning is that both the C6′ amine and the C7’ amide group of the diazepanone ring is methylated by the action of the same N-methyltransferase (Cpz11 or 26) while the methylation at the C7’ amide group takes place after the ring closure (envisioning that both secondary α-amines have a similar spatial geometry and the ring is rotatable at C5’ allowing molecular recognition by the same enzyme). The C7’ carboxyl group, on the other hand, is methylated by the action of the O-methyltransferase (the remaining Cpz11 or 26), of which the resulting methoxyl group turns out to be a good leaving group for the subsequent ring cyclization to take place. This process differs from the canonical amino acid adenylation by reacting with ATP and the β-hydroxyl group appears to be critical for the ring to cyclize. The isolation of compound **13** further suggests that the proposed ribosyltransferase Cpz17 can transfer ADR onto compounds **11** and **12**, while the confirmation is beyond the scope of this study. Next, Cpz10 was examined against compound **13**, which, however, cannot be consumed by Cpz10, suggesting that it is not a substrate of Cpz10. Next, Cpz10 was examined against compound **14** (produced by Cpz27, see below), which was proposed the substrate in the biosynthesis of muraymycin and polyoxin. Again, we did not find any new product^4,20^, concluding that both compounds **13** and **14** are not the substrates of Cpz10 in contradiction to the biosynthetic pathways proposed previously^4,20^ (Supplementary Scheme 2).

### Cpz27 is responsible for phosphorylation of compound 13

In silico analysis, the *cpz* BGC holds two genes *cpz*12 and *cpz*27 both coding for kinase-like proteins (Supplementary Fig. 25). *cpz*27 contains a walker ATP motif (GXXGXGKS/T) in opposite to *cpz*12 that does not; *cpz*27 also shows 24% protein sequence identity to tunicamycin-resistance protein (TmrD)^57^. To elucidate their biochemical roles, we cloned and expressed both genes in *Streptomyces lividans* TK64 given as *E. coli* was not an appropriate host for these two proteins (Supplementary Fig. 26). Both gene products were purified from *Streptomyces* cultures and examined against compound **13** or tunicamycin. None of them accepts tunicamycin, while can only compound **13** be phosphorylated by Cpz27 to form compound **14** at the expense of one molecule of ATP (Fig. 2D, Fig. 3E and Supplementary Fig. 27). However, we could not isolate compound **14** from the culture of ∆*cpz*10 likely due to low yield and/or high instability. As mention above, compounds **13** and **14** are not substrates of Cpz10 at odds with previous reports^4,20^, where muraymycin, capuramycin and caprazamycin are phosphorylated at the corresponding position to abolish their antimicrobial activity (as a mechanism of self-resistance)^5,12,58^. In the present study, this likelihood is low (see below) but still cannot be ruled out. Alternatively, the 3″-OH phosphorylation as a reaction switch was proposed in control of the reaction order^4,20,59^. To know if the phosphorylation is a reaction switch (it takes place before ring cyclization or at the late-stage products), we purified caprazamycins and prepared compound **8** from the pMKBAC02-CPZ-Int containing Streptomyces. Biochemical examination revealed that neither is the substrate of Cpz27, confirming that the phosphorylation takes place before the heterocyclic ring cyclization. As a result, Cpz27 is a phosphotransferase controlling the reaction order but is irrelevant to 7-membered ring cyclization. On the other hand, Mur23 a pyridoxal phosphate (PLP) dependent decarboxylate (Supplementary Fig. 28) was reported catalyzing decarboxylation of 3″-phospho-6′-*N*-2-aminobutanoyl-ADR-GlyU^20^, where the 3″-phosphate is considered essential as Mur23 cannot react without it (Supplementary Scheme 3). This notion does not agree with our result as Mur23 is capable of decarboxylating compound **11** (without the 3″-phosphate group) to compound **15** (molecular ion (M + H)^+^ at *m/z* = 375.05 by 44) (Figs. 2C and 3F). Additionally, Mur23 was first assayed in the presence of compound **13**, then it was assayed in conjunction with Cpz27 against compound **14**. Whichever the condition there is no new peak emerged, suggesting compounds **13** and **14** are not the substrate of Mur23 (Fig. 2C). Taken together, the decarboxylation is independent of phosphorylation, which likely takes place after *β*-hydroxylation, methylation but prior to ring cyclization (Supplementary Fig. 29). Given the existence of muraymycin D4 (ref. ^60^), one should agree that the decarboxylation of compound **11** is independent of the ADR moiety. Mur19 (the ADR transferase), however, cannot transform muraymycin D4 (Supplementary Fig. 1) to muraymycin D2 as well as compound **15** to ADR-**15** in the presence of UDP-ADR, suggesting that ribosylation takes place prior to decarboxylation. Plus, one previous report showed that Mur23 is incapable of decarboxylating compound **16** (without the 3″-phosphoryl group; Supplementary Fig. 1), indicating both ADR and 3″-phosphate groups are critical^20^. These results somehow conflict each other, not least at odds with our result. This inconsistence is indeed not yet reconciled but awaits further clarification in the future.

## Discussion

**Fig. 7:**
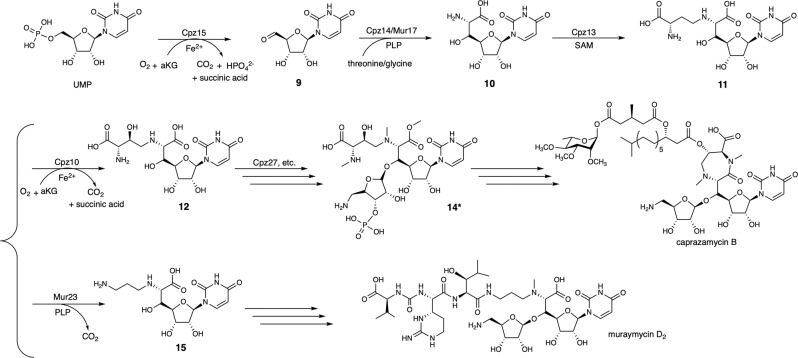
Biosynthesis schemes of caprazamycin and muraymycin. **A** Compound **11** stands at a watershed, of which *β*-hydroxylation catalyzed by Cpz10 permits seven-membered diazepanone ring formation and subsequent acylation to a fully-active caprazamycin or decarboxylation catalyzed by Mur23 directs the synthesis otherwise toward muraymycin. Compound 14* is compound **14** with a OH group at its *β*C position.

The present study elucidated the biochemical roles of selected enzymes involved in the biosynthesis of caprazamycin, particularly two nonheme *α*KG-dependent enzymes Cpz10 and Cpz15 (Supplementary Fig. 30). Cpz15 that was previously assigned as a *β*-hydroxylase turns out to be the starter enzyme converting uridine mono phosphate (UMP) to uridine 5′ aldehyde (U5′A **9**). In contrast, Cpz10 is the actual *β*-hydroxylase in a position to hydroxylate synthetic compound **11** to compound **12** in vitro, standing at a pivotal point to lead the biosynthesis toward caprazamycin. The importance of Cpz10 was parallelly examined in vivo using the in-frame gene-deletion mutant ∆*cpz10*, by which the major product was determined to be compound **13** agreeing with the in vitro result that compound **13** lacks the *β*-OH group. Compound **13** carries two unexpected methyl groups at 6′-amino and 7′-carboxyl in addition to one ADR at 3′-OH. None of compounds **13** and **14** was accepted by Cpz10, suggesting that *β*-hydroxylation takes place earlier than methylation, glycosylation and phosphorylation. Compound **11** is likely the physiological substrate for Cpz10; beyond that, Cpz10 oxygenates *L*-methionine to MSO as a sideline activity. MSO, a nonproteinogenic amino acid exhibits in *N*-acetylmureidomycin E, may be useful as a building unit concerning combinatorial biosynthesis as an approach to generate unnatural natural products (Supplementary Fig. 1)^61,62^. In light of *β*-OH that bridges a long aliphatic side chain and permits the linear chain to form the seven-membered heterocyclic ring, the Cpz10-mediated *β*-hydroxylation appears to be the determining step in the biosynthesis toward the successful production of caprzamycin. Concerning compound **14**, Cpz27 is the phosphotransferase to phosphorylate compound **13** at its 3″-OH with a 3″-phosphate group (compound **14**). Based on protein blasting, there are 29 BGCs for the class of nucleoside antibiotics reported thus far, while only do caprazamycin, A-90289 and liposidomycins contain two putative kinases^4^. Cpz27 out of the two putative kinases is the one directing the formation of compound **14**, while it conflicts with 3″-phospho-caprazol that was previously reported to be the product. In contrast, Mur23 is determined to decarboxylate compound **11** that also contradicts to the report of Cui, Z. et al., in which they specified that Mur23 can only accept the substrate with 3″-phosphate^20,60^. Given caprazmycin (**2**) and muraymycin (**6**), one can appreciate that compound **11** is a watershed, of which *β*-hydroxylation catalyzed by Cpz10 permits the heterocyclic ring formation and subsequent acylation to a fully-active caprazamycin or decarboxylation catalyzed by Mur23 otherwise leads the synthesis to muraymycin (Fig. 7). To this end, we conclude that the *β*-hydroxylation of compound **11** takes place prior to 3′-glycosylation, 6′- and 7′-methylation, 3″-phosphorylation and the heterocyclic ring cyclization, and the formation of caprazol **8**.

X-ray crystal structures revealed that Cpz10 contains two irons (Fe1 and Fe2) in 12 Å away from each other. Fe1 sits at the active center held by the His115, His160 and Asp117 triad together with *α*KG and a water molecule (Wt91; at the sixth coordinate) to form a tetragonal bipyramidal coordination, a typical geometry in the nonheme *α*KG-dependent protein family. Compound **11** is placed above the sixth coordinate with its pro-*S* H atom of *β*-carbon pointing toward the sixth coordinate, thus accounting for the regioselectivity and stereoselectivity in the Cpz10-mediated reactions. Nonheme *α*KG-dependent enzymes that contain two irons are not uncommon but often ignored^42,63–65^. The second iron (Fe2) does not appear in random but exists in a deliberately organized conduit ideally for electron/charge transfer. As a matter of fact, Fe2 is not confined in a fixed position but dynamically interacts with a constellation of residues in the tunnel; this observation is line with the NBO calculation. On top of that, EPR spectra reflect signal changes for both Fe2 (2.02 → 1.97 g) and Fe1 (4.75 → 4.25 g) in the absence or presence of substrates in the Cpz10-mediated reaction, echoing dynamic changes in electron/charge flow, oxidation status, coordination geometry, etc^42^.

From the viewpoint of crystal field theory (CFT)^66,67^, the five-degenerate d-orbitals in Fe^II^ and Fe^III^ are reorganized by the static electric field arising from the distribution of charges upon association with the coordinate residues, *α*KG, and dioxygen/water molecules. In the case of Fe^III^ with octahedral molecular geometry, the crystal filed splits its five d-orbitals into two sets with different energy levels. The lower set, which is a collection of the three lower-energy orbitals of the d_xy_, d_xz_, and d_yz_, is referred to as t_2g_. The upper set, which consists of the two higher-energy orbitals of the d_x_^2^_-y_^2^ and d_z_^2^, is referred to as e_g_ (refs. ^68,69^). From the quantum–mechanical point of view^70^, five d-electrons in 3d orbitals of the Fe^III^ are distributed in three different spin states, including low-spin (LS) state (S = 1/2), intermediate-spin (IS) state (S = 3/2), and high-spin (HS) state (S = 5/2), in these t_2g_ and e_g_ orbitals. The spin states of 1/2, 3/2, and 5/2 are assigned to electron-spin multiplicities, 2 S + 1, of doublet, quartet, and sextet, respectively. The Fe^II^ has tetrahedral molecular geometry in which the splitting of 3d orbitals is the opposite of that in octahedral geometry, namely, three higher-energy orbitals of the d_xy_, d_xz_, and d_yz_ are located above two lower-energy orbitals of the ${{{{{\rm{d}}}}}}_{{{{{{{\rm{x}}}}}}^{2}}-{{{{{\rm{y}}}}}}^{2}}$ and ${{{{{\rm{d}}}}}}_{{{{{{\rm{z}}}}}^2}}$ (refs. ^68,69^). The occupancies of the six d-electrons of Fe^II^ in the e_g_ and t_2g_ orbitals are as three spin states of 0 (LS), 1 (IS), 2 (HS) attributing to multiplicities of singlet, triplet, and quintet, respectively^70^. Indeed, on the basis of NBO results for Fe1 and Fe2, we conclude that another possible role of two iron cations is playing an essential role in an electron transfer process within the Cpz10. In this study, both complex structures and EPR examination have demonstrated that the second iron plays significant role to replace the succinic acid to new *α*KG. This new phenomenon not only controlling the reaction inhibitors but also accelerating the reaction. An important feature of iron that makes it appealing for biological redox processes is the easy one-electron interconversion of Fe^II^ and Fe^III^ (ref. ^71^). This discovery in the caprazamycin biosynthetic pathway has important implications for our comprehensive understanding of reaction mechanisms of nonheme metal iron-dependent enzymes, which is in support of our previous quantum chemistry analysis where key components of caprazamycin contribute significantly to the high binding affinity with MraY^5^.

In summary, the biosynthesis of nucleoside antibiotics has been studied to some extent while it is still far from completion^8,72,73^. The present study concludes that the *β*-hydroxylation catalyzed by Cpz10, a nonheme dioxygenase, plays a critical role governing the subsequent heterocyclic ring formation as well as the long aliphatic sidechain acylation toward full-blown bioactivity of caprazamycin. By contrast, the decarboxylation of the 6′-*N*-alkylamine side chain catalyzed by Mur23, a PLP dependent decarboxylase, diverges the biosynthesis toward the Park’s nucleotide subgroup. In conjunction with biochemical determination of other related enzymes, the in vitro, in vivo and biochemical/biophysical profiling has not only met our scientific interest with respect to the biosynthesis of caprazamycin but also paved a foundation for pathway engineering toward more effective nucleoside antibiotics in a way to help conquer the most formidable adversary multidrug-resistant pathogens that man is facing now.

## Experimental section

### General chemicals, reagents and analytical methods

Those chemicals are commercially unavailable were chemically (see Supplementary methods) and enzymatically synthesized, but most of the reagents were purchased from commercial sources without further purification. NMR spectra were collected by the Bruker AV600-MHz spectrometer, a two-channel system equipped with 5 mm DCI Dual cryoprobe. NMR spectra were processed and analyzed with SpinWorks v.3.0. High-performance liquid chromatography (HPLC) analysis was conducted on an Agilent 1200 series HPLC system consisting of G1311A pump, G1316A column oven, G1367B autosampler and G1315D diode array equipped with an ODS Prodigy column (Phenomenex) or an Xbridge Amide column (5 μm, 4.6 × 250 mm, Waters). High resolution and high mass accuracy experiments were done on an LTQ Orbitrap XL ETD mass spectrometer (Thermo Fisher Scientific, San Jose, CA) equipped with a standard ESI ion source. 5 μL of samples were flow injected at a rate of 50 μL min^−1^ in 80% ACN/H_2_O with 0.1% formic acid to the Waters Acquity UPLC (Waters, Milford, MA). Full-scan MS condition is the mass range at m/z 200–2000, the resolution 60,000 at m/z 400. Electrospray voltage was maintained at 4 kV and capillary temperature was set at 275 °C. For LC–ESI–MS/MS, samples were detected by LC-ESI-MS on an Orbitrap Fusion mass spectrometer (Thermo Fisher Scientific, San Jose, CA) equipped with EASY-nLC 1200 system (Thermo, San Jose, CA, US) and EASY-spray source (Thermo, San Jose, CA, US). The digestion solution was injected (5 μL) at 1 μL min^−1^ flow rate to an easy column (C_18_, 0.075 mm × 150 mm, ID 3 μm; Thermo Scientific). Chromatographic separation was using 0.1% formic acid in water as mobile phase A and 0.1% formic acid in 80% acetonitrile as mobile phase B operated at 300 nL min^−1^ flow rate. Briefly, the gradient employed was 2% buffer B at 2 min to 40% buffer B at 40 min. Full-scan MS condition: mass range m/z 375–1800 (AGC target 5E5) with lock mass, resolution 60,000 at m/z 200, and maximum injection time of 50 ms. Target m/z were isolated for CID with NCE35 and maximum injection time of 100 ms. Electrospray voltage was maintained at 1.8 kV and capillary temperature was set at 275 °C. High resolution mass spectrometry analyses were performed by Mass Core Facility of GRC, AS. Size exclusion column chromatography analyses were conducted on an AKTA purifier (GE Healthcare Life Sciences) with a Superdex 200 16/600 GL column (GE Healthcare Life Sciences, UPC-900 and P-920). pGUSRolRPA3 was provided as a gift by the laboratory of Prof. Andriy Luzhetskyy (Helmholtz Institute for Pharmaceutical Research Saarland, Saarland University). *E. coli* ET12567[pUZ8002] was provided as a gift by the laboratory of Prof. Chih-Hung Huang (Laboratory of Bacterial Genetic, National Taipei University of Technology, Taipei, Taiwan).

### Bacteria strains and plasmids used in this study

*E. coli* strains were grown in Luria-Bertani (LB) medium supplemented with either apramycin (50 µg mL^−1^), kanamycin (35 µgmL^−1^) or chloramphenicol (35 µg mL^−1^). The caprazamycin producing strain and the host strain for heterologous expression, *Streptomyces* sp. MK730-62F2 and *Streptomyces albus* J1074::ErmE*-*crpsc*, were grown at 28 °C with MS medium agar (2% soy flour, 2% mannitol, 2% agar). apramycin (50 µg mL^−1^) and nalidixic acid (25 µg mL^−1^) were supplemented for conjugation. BAC clones with large inserts were isolated using the ZR BAC DNA Miniprep Kit (ZYMO research). Transformation of BAC plasmid into *E. coli* was performed using 10 G BAC-Optimized Electrocompetent Cells (Lucigen).

### Construction of pMKBAC02

The origin of transfer region (*ori*T) and apramycin resistance gene, *aac*III(IV), were amplified from plasmid pGUSRolRPA3^74^ using the primer set 325-oriT-Am-F (5′-ATTACCACCTTAATTAAGGATCCGAATTCGAAGATCCTTTGATCTTTTC′) and 326-oriT-Am-R (5′-ATTATTATTCCTAGGAGCTTGCATGCCTGCAGGTCGA-3′). The backbone of the pMKBAC02 vector was amplified from plasmid pBeloBAC11 (New England Biolabs, NEB) using primer set p327-BAC-F (5′-ATTATTATTCCTAGGGTTTAAACAGGGCTTCCCGGTATCAAC-3′) and p328-BAC-R (5′-ATTACCACCTTAATTAAAAGCTTGGTTACTCCGTTCTACAGGTTAC-3′). The 2.1 kb PCR product amplified from the first primer set and the 5.4 kb PCR product amplified from the second primer set were digested within AvrII and PacI individually and then ligated together to produce plasmid pMKBAC02.

### Isolation of entire caprazamycin aglycone biosynthetic gene cluster into pMKBAC02

To isolate the CPZ BGC from the chromosome of *Streptomyces sp*. MK730-62F2, a homologous DNA fragment including a portion of CPZ amplified within 537-pMKBAC02-cpz-F (5′-ATTATTATTAAGCTTGTACACACAGCACTACCAGCACCACTTCG) and 538-pMKBAC02-cpz-R (5′- ATTATTATTGAATTCTCGTCCACGGTCGTCAGGAACAGCTGTA-3′) was cloned into pMKBAC02 within HindIII and EcoRI restriction enzyme sites respectively to generate pMKBAC02-CpzH. Conjugation was later performed to integrate pMKBAC02-CpzH into the chromosomal DNA of Streptomyces sp. MK730-62F2 via homologous recombination as. Next, the qualified mutants were carefully chosen on apramycin containing MS agar^75^, verified using PCR within primer sets, p574-check-genome-BAC-cpz-F (5′- TGCACATGAACCAAAAGGATCTAGGTGAAGATCCTTTTTGATAA-3′) and p575-check-genome-BAC-cpz-R (5′- GAGTCGTCGTGCAGTTGCTCTTCGGACA-3′) (Supplementary Fig. 22). This strain was cultured in TSB media for 1 day at 30 °C, after which its genomic DNA was prepared and then digested by restriction enzyme HindIII. The digested DNA fragments were purified and concentrated by ethanol precipitation before self-ligation using T4 ligase (Thermo; Fisher Scientific)). The ligation mixture was used for electroporation of 10 G BAC-Optimized Electro-competent Cells. Recombinants were selected on apramycin-supplying LB medium gar, after which plasmids were verified by PCR using primers, P238-check-pMKBAC02-F (5′-TAATGTCCTTTGTTACAGGCCAGAAAGCATAA-3′) and P239-check-cpz4 (5′-ATGAACGCCTCCTTCAAGGATCCGTT -3′), the BAC plasmid carrying CPZ aglycone BGC was identified as pMKBAC02-CPZ (Supplementary Fig. 22). The DNA fragment containing attP-intΦC31 moiety was amplified from plasmid pGUSRolRPA3 within primer sets, 329-AvrII-attP-int-phiC31-F (5′-ATTATTATTCCTAGGCTAGCGATTCCAGACGTCCCGAAG-3′) and 330-AvrII-attP-int-phiC31-R (5′-ATTATTATTCCTAGGAATTCCCCAATGTCAAGCACTTCCGG-3′). The amplicon was digested and cloned into pMKBAC02-CPZ by AvrII restriction enzyme site for generating pMKBAC02-CPZ-Int.

### Generation of *cpz*10-deficient mutants via in vitro CRISPR-Cas9 digestion

In vitro CRISPR-Cas9 digestion was performed as previous described^76^. Briefly, the gene sequence to be edited was screened for the presence of a 20-bp guide and PAM sequence (5′-N20NGG-3′) for sgRNA design. Transcription templates of the sgRNA were amplified by overlap extension PCR with the primers, 893-sgRNA-Dcpz10-1 (5′-GATCACTAATACGACTCACTATAGTCTTTCCAACAACTCCACGAGTTTTAGAGCTAGAAATAGCAA-3′), 894-sgRNA-Dcpz10-2 (5′-GATCACTAATACGACTCACTATAGCGTTCAAGCCCATTCGCACGGTTTTAGAGCTAGAAATAGCAA-3′), 229-sgRNA-F (5′-GTTTTAGAGCTAGAAATAGCAAGTTAAAATAAGGCTAGTC-3′) and 230-sgRNA-R (5′-AAAAGCACCGACTCGGTGCCACTTTTTCAAGTTGATAACGGACTAGCCTTATTTTAACT-3′).

### In vitro transcription of sgRNA was performed using the TranscriptAid

T7 high-yield transcription kit (Thermo; Fisher Scientific) pursuant to the manufacturer’s instructions. The sgRNA product was pretreated by heating at 95 °C for 10 min and slowly cooling down to room temperature.

In vitro Cas9-mediated editing was performed following the protocol of Cas9 nuclease, *S. pyogenes* (NEB). First, a 30 μL reaction mixture containing 30 nM Cas9 protein, 30 nM sgRNA and 3 nM DNA was prepared and incubated in buffer 3.1 (New England Biolabs) at 37 °C for 2 h.

The reaction was terminated by addition of 0.2 mg mL^−1^ RNase and continued incubation at 37 °C for 15 min. Then the reaction mixture was treated with 1 mg mL^−1^ proteinase K (Thermo; Fisher Scientific) and incubated at 37 °C for 30 min. Finally, the Cas9-digested DNA was recovered by ethanol precipitation and self-ligated in the ligation mixture and then the mixture was incubated at 16 °C for overnight.

The ligation mixture was used for electroporation of 10 G BAC-Optimized Electrocompetent Cells. Gene-deficient mutants were selected on apramycin-adding medium gar, after that the plasmids were verified by PCR using primers, 895-check-Dcpz10-F (5′-CTCATGACGCGGATCCGAGGATTCAT-3′) and 896-check-Dcpz10-R (5′-AATCCTGGCAGGTCCTTTCCCGCTT-3′) (Supplementary Fig. 22).

### Cloning for gene expression

All genes in caprazamycin and muraymycin gene clusters were cloned from the genomic DNA of producing strains by applying to standard procedures (primers listed in Supplementary Table 1A, B). The PCR products for *cpz*15 and *cpz* 10 were digested with EcoR1-HinDIII and ligated to the appropriate sites of pET28a to yield pET28a- *cpz*15 and pET28a- *cpz*10, respectively. PCR results were confirmed by DNA sequencing. The PCR products for *cpz*27 and *cpz*12 were digested with Nde1-HidIII, sequenced and inserted into a pET28a (pET28a- *cpz*27 and pET28a- *cpz*12) vector by using ligation-independent cloning, following the commercial protocol. The PCR products for *mur*16, *mur*17 and mur23 were digested with NdeI_XhoI and ligated to the appropriate sites of pET28a to yield pET28a-*mur*16, pET28a-*mur*17 and pET28a-*mur*23, respectively. Moreover, *cpz*27 was cloned to PGM1202 plasmid to yield PGM1202-*cpz*27 by using AseI_HindIII enzymes.

### Site-directed mutagenesis

R170A, D117G, D117A, M104A, M104L, D72A, D72V, R102A, R102V, R121A, and R121V point mutations of *cpz*10 and a R259A point mutation of *cpz*15 were generated by PCR amplification with high-fidelity, hot start, DNA polymerase using pET28-*cpz*10 or pET28-*cpz*15 as a template, respectively. The templates pET28a-*cpz*10 and pET28a-*cpz*15 were obtained by using ligation-independent cloning following the commercial kit protocol. The PCR products were digested with 10 units DpnI for overnight at room temperature and transformed into *E. coli* DH5*α*-competent, then BL21-competent cells. The entire gene sequences, including of the correct point mutation sites, were confirmed by sequencing.

Recombinant protein production. Plasmids pGM1202-*cpz*27 was transformed into *S. lividans* TK64 by applying PEG (poly ethylene glycol)-mediated protoplast transformation and plated on R5 medium containing 50 μg mL^−1^ of apramycin. After the Incubation at 28 °C for 6 days, successful transformants were affirmed by colony PCR. Successfully transformed strains were applied to inoculate 50 ml YEME medium containing 50 μg mL^−1^ of apramycin, incubated for 3 days at 28 °C and then 4 mL was transferred to 400 mL fresh YEME medium including 50 μg mL^−1^ of apramycin. After 36 h incubation at 28 °C, protein (Cpz27) expression was induced by the amount of thiostrepton (5 μg mL^−1^) and before harvesting for more 36 h the culture was incubated. The cells from 400 ml of the culture were harvested by centrifugation. The pellet was completely re-suspended in ice-cold buffer D (100 mM Tris-HCl, 300 mM KCl, 50 mM arginine, 1% glycerol pH 8.0), and extra 4 mg mL^−1^ of lysozyme was later added to the suspension. After incubation at 30 °C for 30 min, the cell suspension was lysed and micro-fluidized at 150 MPa in a Microfluidizer processor model M-110 (Quadro Engineering Corp.) or sonicated with Misonix sonicator for 15 min. After centrifugation, the protein was purified by applying the affinity chromatography with IMAC sepharose 6 fast flew resin, and proteins were eluted with high concentrations of imidazole in buffer B (100 mM HEPES, 300 mM KCl, 50 mM arginine, 1% Glycerol, 400 mM imidazole, pH 8.0). Purified proteins were concentrated and buffer exchanged into buffer C (17 mM KH_2_PO_4_, 33 mM K_2_HPO_4_, pH 8.0) by using Amicon Ultra 10 K MWCO centrifugal filters and applying fresh for storage at −80 °C. Protein purity was assessed using sodium dodecylsulfate (SDS)–15 and 10% poly acrylamide gel electrophoresis; His_6_-tagged proteins were used without further modifications^77^.

All the plasmids; pET28a-*cpz*10, pET28a-*cpz*10 (all mutants), pET28a-*cpz*15, pET28a-*cpz*15 (R259A), pET28a-*cpz*12, pET28a-*mur*16, pET28a-*mur*17 and pET28a-*mur*23 were introduced into BL21(DE3), and the transformed strains were grown in LB supplemented with 50 μg mL^−1^ of kanamycin. After inoculation of 30 ml lysogeny broth with 3 μg mL^−1^ of kanamycin, the cultures were grown at 37 °C until the cell density reached an A_600_  ≈  0.7, when expression was induced with 0.3 or 0.5 mM isopropyl *β*-D-1-thiogalactopyranoside. Cells were harvested and processed as described for Cpz27 but with different buffers.

### Reactions with Cpz15 and Mur16

Cpz15 and a mutant and Mur16 were lysed with buffer D: 100 mM Tris-HCl, 300 mM KCl, pH 8.0 and protein was eluted by with buffer E: 100 mM HEPES, 300 mM KCl, Imidazole 50 mM, pH 8.0. Reaction mixtures consisted of 100 mM HEPES (pH 8.0), 100 mM KCl, 1 mM UMP (uridine monophosphate), 1.5 mM *α*KG, 200 μM ascorbate, 100 μM FeSO_4_, and 100 ng Cpz15 and a mutant for 3 h at 28 °C, and the reactions were subsequently quenched by adding a volume of chloroform followed by centrifugation (15000 rpm, 30 min) to separate two phases then remove the precipitated proteins. The reactions were monitored by LC–MS with a prodigy column (5 µm, 100 Å, 250 mm × 4.6 mm) and HPLC with a SOURCE™ 15Q 4.6/100 PE column for ion exchange chromatography. A series of linear gradients was developed from water with 0.1% Trifluoroacetic acid (mobile phase A) to acetonitrile with 0.1% Trifluoroacetic acid (mobile phase B) in the following manner (beginning time and ending time with linear change to percentage B): 0–6 min, 0.5% B; 10–13 min, 90% B; 15–20 min, 0.5% B. The flow rate was kept constant at 1 mL min^−1^ and elution was monitored at 260 nm.

### Activity of Mur17

Reaction mixtures consisted of 50 mM potassium phosphate (pH 8.3), 2 mM U5’A, 2 mM Thr/Ser/Gly, 500 μM PLP and 200 nM Mur17 for 5 h at 30 °C, and the reaction was terminated by adding a volume of chloroform followed by centrifugation. The reaction components were analyzed by LC–MS with a prodigy column (5 µm, 100 Å, 250 mm × 4.6 mm) column as described in reactions with Cpz15 and Mur16.

### Reaction with Cpz10

Cpz10 and all mutants were lysed with buffer D: 100 mM Tris-HCl, 300 mM KCl, pH 8.0 and protein was eluted with buffer F: 100 mM HEPES, 300 mM KCl, imidazole 400 mM, pH 7.5. Purified proteins were concentrated and buffer exchanged into buffer G (17 mM KH_2_PO_4_, 33 mM K_2_HPO_4_, pH 7.5) by using Amicon Ultra 10000 MWCO centrifugal filters. Reaction mixtures consisted of buffer G, 2 mM compound **9** or methionine, 1.5 mM *α*KG, 200 μM ascorbate, 100 μM FeSO_4_, 100 ng Cpz10 and the mutated proteins for 3 h at 28 °C, and the reactions were subsequently quenched by adding a volume of chloroform followed by centrifugation (21,000 g, 30 min) to remove the precipitated proteins. The reactions were monitored by LC–MS with a prodigy column (5 µm, 100 Å, 250 mm × 4.6 mm) and for HPLC with SOURCE™ 15Q 4.6/100 PE column for Ion exchange chromatography. A series of linear gradients was developed from water with 0.1% Trifluoroacetic acid (mobile phase A) to acetonitrile with 0.1% Trifluoroacetic acid (mobile phase B) in the following manner (beginning time and ending time with linear change to percentage B): 0–6 min, 0.5% B; 10–13 min, 90% B; 15–20 min, 0.5% B. The flow rate was kept constant at 1 mL min^−1^ and elution was monitored at 260 nm for compounds **11**, **12**, **13**, and for methionine and MSO was 210 nm.

### Reactions with Cpz27 and Cpz12

Reaction mixtures comprised of 50 mM potassium phosphate (pH 8.0 for Cpz27 and pH 7.0 for Cpz12), 500 μM **13**, 1 mM ATP, 1 mM MgCl_2_/MgSO_4_, and 200 nM Cpz27 and Cpz12 for 3 h at 28 °C, and the reaction was later quenched by adding two volumes of acetonitrile followed by centrifugation (15000 rpm, 30 min) to remove the precipitated protein. The reaction was monitored by LC–MS or HPLC with a prodigy column (5 µm, 100 Å, 250 mm × 4.6 mm). A series of linear gradients was developed from water with 0.1% trifluoroacetic acid (mobile phase A) to acetonitrile with 0.1% Trifluoroacetic acid (mobile phase B) in the following: 0–10 min, 2.0% B; 10–20 min, 35% B; 24–34 min, 98% B; 35–40 min, 2% B. The flow rate was kept constant at 1 mL min^−1^ and elution was monitored at 260 nm.

### Reaction with Mur23

Reaction mixtures consisted of 50 mM HEPES (pH 8.3), 1 mM **11**, 1 mM PLP and 200 nM Mur23 for 1 h at 30 °C, and the reaction was terminated by adding a volume chloroform followed by centrifugation. The reaction was monitored by LC–MS or HPLC with a prodigy column (5 µm, 100 Å, 250 mm × 4.6 mm). A series of linear gradients was developed from water with 0.1% trifluoroacetic acid (mobile phase A) to acetonitrile with 0.1% trifluoroacetic acid (mobile phase B) in the following manner (beginning time and ending time with linear change to percentage B): 0–10 min, 2.0% B; 10–20 min, 35% B; 24–34 min, 98% B; 35–40 min, 2% B. The flow rate was kept constant at 1 mL min^−1^ and elution was monitored at 260 nm.

### Purification of *cpz*10-knockout intermediate

The compound **13** as a knockout intermediate was purified using culture condition pH (6–7), then centrifuge the culture broth for 30 min in 6000 rpm. After separating broth, it’s pH decrease to 4 for remain proteins aggregation. Centrifuge again same condition. Collected broth first mixed with ethyl acetate (EA) then butanol to separate hydrophobic compounds from the collected broth. Remain mix was added with Diaion HP20 resin (Sigma-Aldrich) for overnight in 4 °C, and washed the by water. The target compound was eluted out by the acetone, and dried with the rotary vapor. After rehydrating by the DD water, the intermediate was loaded to Sephadex LH20 (GE Healthcare) column for further purification. Eluate was collected, and loaded on CM Sephadex C-25 to separating sugar impurities. Collected remainder was injected to HPLC with a prodigy column (5 µm, 100 Å, 250 mm × 4.6 mm). A series of linear gradients was same as previously mentioned. The final compound was checked by HR-MS and NMR.

### UV/Vis spectral analysis

Absorbance spectra of the analytes were recorded on a Beckman coulter DU 800-spectrophotometer using 30 μM indicated protein, with a range from 0.1 to 1 mM of the respective substrates (**9** and *L*-methionine) at room temperature in buffer G supplemented with 100 μM FeSO_4_ and 200 μM ascorbate. The spectra were recorded every 1 min and 5 min.

### Crystallization and data collection

Cpz10 and its mutants were crystallized by using hanging drop and sitting drop vapor diffusion methods at 20 °C, 16 °C and 4 °C. A total of 10 mg and 5 mg of Cpz10 in 10 mM HEPES (pH 7.0, 100 mM KCl) were mixed with the same volume of reservoir solution for the crystallization. Octahedron crystals (Supplementary Fig. 10) can be obtained in 5 days. The crystalized condition for the apo form protein is 100 mM calcium acetate, 100 mM MES (pH 6.5), 9% (v/v) PEG 4 K; the other condition for complex screening is 10 mg and 5 mg Cpz10 in 10 mM K_2_HPO_4_ (pH 7.0), 0.1 mM FeSO_4_ and 6 mM *α*KG, contains 0.2 M potassium/sodium tartrate, 0.1 M Bis Tris propane (pH 6.5), 20% (w/v) PEG 3350. Moreover, for mutants, first Cpz10-D117G crystals were obtained in following crystallization condition: 0.2 M calcium acetate hydrate, 20% (w/v) PEG 3350, (pH 7.5) and for Cpz10-R170A: 0.1 M lithium sulfate monohydrate, 0.1 M ADA (pH 6.5), 12% PEG 4 K and 2% v/v 2-propanol. For the soaking condition, an overnight before collecting data, the crystals were exposed to glutaraldehyde. X-ray diffraction data sets were collected on an ADSC Quantum-315 or MX300HE CCD detectors at beamlines 13B1, 13C1, 15A1, 05 A of the National Synchrotron Radiation Research Center (Taiwan), or the beamline 44XU of the Spring-8 (Japan).

### Structure determination and refinement

The data sets were processed and scaled with HKL-2000^78^. The crystal structures of Cpz10 was determined by molecular replacement (MR) and a PHASER software in CCP4^79^. The crystal structure of Hyps (Protein Data Bank (PDB) code 4P7W) was used as the search model. All structures were further refined using REFMAC^80^. Subsequent iterative cycles of model building and refinement were performed by COOT^81^ and PHENIX^82^. All non-hydrogen atoms were refined with anisotropic displacement parameters (ADP). Both rigid body and real space refinement were applied. Both bulk-solvent correction and anisotropic scaling were also implemented. Refinement was carried out using the conjugate-gradient least-squares method in SHELXL, in which non-geometric restraints were applied for Cpz10 conformers. The weights for stereochemical and ADP restraints were optimized by scaling in the PHENIX refinement. Structure models and electron density maps were presented using PyMOL^83^.

### Computational methods

To characterize accurately the physical nature of the intermolecular interactions in the bonds of residue–Fe, *α*KG–Fe, and Wt–Fe, the active site and the Fe–binding site of Cpz10 were undergone natural bond orbital (NBO) analysis^51,52^. To attain this objective, we firstly designed two structural models, one of which includes the Fe (III) cation, Wt91, *α*KG, and the cited residues, named model I, and the other consists of the Fe (II) cation, Wt56 and Wt89 in addition with the specified residues, named model II. Because of the lack of coordinates of hydrogen atoms in the X-ray structure of Cpz10, each structural model was partly optimized to correctly determine the positions of hydrogen atoms by using the unrestricted hybrid meta–GGA density functional (UM06-2X)^84^ and the standard basis sets of 6–31 G** (for C H N O atoms) and Lanl2dz (for the Fe cations)^85^. The coordinates of non-hydrogen atoms were frozen during the geometry optimization. For the LS, IS, and HS states of Fe (III), the structural model I was separately optimized in three multiplicities of doublet, quartet, and sextet. Likewise, for the LS, IS, and HS states of Fe (II), the geometry optimization of the structural model II was discretely carried out in multiplicities of singlet, triplet, and quintet. Afterwards, NBO analysis was performed on each optimized structural model at the same computational level. All density functional theory (DFT) calculations were done by means of the Gaussian 09 software^86^.

### Setup CPZ–*α*-KG Complex for MD simulation

The crystal structure of CPZ–*α*KG was used as initial coordinateness for molecular dynamics (MD) simulation utilizing NAMD program package^87^. To attain this objective, firstly, the protein topology file of CHARMM36 force field^88^ was applied to create topology entries of *α*KG and Fe ions as well as to add the missing hydrogen atoms to the non-hydrogen atoms of CPZ protein by employing the VMD software^89^. Secondly, the CPZ–*α*KG complex was located in a periodic rectangular box comprising a 12 Å layer of TIP3P water molecules. Afterwards, counter sodium and chloride ions were added to the surface of protein to neutralize the total charge of the protein–water system. Thirdly, this neutral solvated CPZ–*α*KG system was initially simulated for 2 ns in the NPT ensemble at 310 K and 1 atm and then the MD simulation was continued for 10 ns in the NVT ensemble. Simulations in both ensembles were performed with time step 1 fs and by using the SHAKE algorithm. Particle Mesh Ewald (PME) approach was applied to calculate the electrostatic interactions, whereas a 12 Å cut off was imposed on van der Waals interactions modeled by the Lennard–Jones potential.

## Supplementary information

Supplementary Information

Description of Additional Supplementary Files

Li_PR File

Supplementary data 1

Supplementary data 2

Supplementary data 3

Supplementary Video 1

Supplementary Video 2

## Data Availability

Relevant data are available from the corresponding author on reasonable request. The atomic coordinates and structure factors of Cpz10 apo form (PDB ID code: 7V4N), Cpz10/*α*KG (PDB ID code: 7V4M), Cpz10/SA/compound **12** (PDB ID code: 7V4F), Cpz10 R170A mutant (PDB ID code: 7V4O), Cpz10 D117G mutant (PDB ID code: 7V4P). Two pdb files for CPZ-ligand configurations at initial and final states were provided in Supplementary Data 1 and Data 2, two DFT atomic coordinates of the optimized computational models were provided in Supplementary Data 3 and two Supplementary Movies were provided in Supplementary Movie 1 and Supplementary Movie 2.
