# Supplementary material for: β-Hydroxylation of α-amino-β-hydroxylbutanoyl-glycyluridine catalyzed by a nonheme hydroxylase ensures the maturation of caprazamycin
Source: Commun Chem. 2022 Jul 28;5:87. doi: 10.1038/s42004-022-00703-6 (PMC9814697; doi:10.1038/s42004-022-00703-6)
Supplement: Supplementary file 1 — Supplementary Information [file 42004_2022_703_MOESM1_ESM.pdf]

Supplementary Information

β-Hydroxylation of *α*-amino-*β*-hydroxylbutanoyl-glycyluridine catalyzed by a nonheme hydroxylase ensures the maturation of caprazamycin

Saeid Malek Zadeh[1,2,3], Mei-Hua Chen[1,2,4], Zhe-Chong Wang[1], Elahe K. Astani[5], I-Wen Lo[1], Kuan-Hung Lin[1], Ning-Shian Hsu[1], Kamal Adhikari[1,6], Syue-Yi Lyu[1], Hsin-Ying Tsai[1], Yuma Terasawa[7], Miyuki Yabe[7], Kazuki Yamamoto[7], Satoshi Ichikawa[7,8] & Tsung-Lin Li[1,2,5,9]*

[1] Genomics Research Center, Academia Sinica, Taipei 115, Taiwan

[2] Chemical Biology and Molecular Biophysics Program, Taiwan International Graduate Program, Academia Sinica, Taipei 115, Taiwan

[3] College of Life Science, National Tsing Hua University, Hsinchu 300, Taiwan

[4] Department of Chemistry, National Taiwan University, Taipei 106, Taiwan

[5] Department of Chemistry, Faculty of Science, Tarbiat Modares University, Tehran 14115-175, Iran

[6] Molecular and Biological Agricultural Sciences Program, Taiwan International Graduate Program, Academia Sinica and National Chung Hsing University, Taipei 115, Taiwan

[7] Graduate School of Pharmaceutical Sciences, Hokkaido University, Sapporo 060–0812, Japan

[8] Global Station for Biosurfaces and Drug Discovery, Global Institution for Collaborative Research and Education (GI- CoRE), Hokkaido University, Kita-12, Nishi-6, Kita-ku, Sapporo 060-0812, Sapporo, Japan

[9] Biotechnology Center, National Chung Hsing University, Taichung City 402, Taiwan

* Correspondence: tlli@gate.sinica.edu.tw

This supplementary information includes:

Supplementary Methods

Figures S1 to S30

Supporting Note 1

Tables S1-S3

Scheme S1-S3

References 1-4

Supplementary Methods

6-Amino-1-(uracil-1-yl)-6-deoxy-β-D-*glycero*-L-*talo*-heptofuranuronate (**10**)

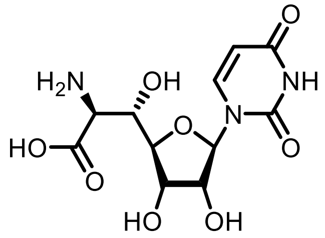

Chemical Formula: $C_{11}H_{15}N_3O_8$
Exact Mass: 317.09
Molecular Weight: 317.25

A suspension of methyl 6-benzyloxycarbonylamino-1-(3-benzyloxymethyluracil-1-yl)-6-deoxy-2,3-*O*-isopropylidene-β-D-*glycero*-L-*talo*-heptofuranuronate[1] (50 mg, 98.9 μmol) and trimethyltin hydroxide (90.4 mg, 500 μmol) in 1,2-dichloroethane (1 mL) was heated to 90 °C. After stirring for 2.5 h, the reaction mixture was cooled and silica gel was added to the mixture. The solvent was removed *in vacuo*, and the resulting silica gel was applied to short silica gel pad and eluted with 5% MeOH/$CHCl_3$. The filtrate was concentrated *in vacuo* to afford the crude carboxylic acid (16.7 mg). A solution of the carboxylic acid in CH_2Cl_2 (0.3 mL) was treated with BCl_3 (1 M in CH_2Cl_2, 68 μL) at −78 °C and the reaction mixture was stirred at 0 °C for 1 h. The reaction mixture was cooled to −78 °C and BCl_3 (1 M in CH_2Cl_2, 68 μL) was added. The reaction mixture was stirred at 0 °C for 45 min. Methanol (1 mL) was added to the reaction mixture, and the solution was concentrated *in vacuo*. Because the isopropylidene group remained from mass analysis, the residue was treated with 80% *aq.* TFA (0.5 mL) for 15 min. The reaction mixture was concentrated *in vacuo* and the residue was purified by ODS silica gel column chromatography (MeCN/H_2O 5 to 50% with 0.1% TFA) to afford **10** (4.1 mg, 10%) as a white solid.

^{1}H NMR (D_2O, 400 MHz) δ 8.05 (d, 1H, H-6, $J_{6,5}$ = 8.4 Hz), 5.91 (d, 1H, H-1', $J_{1',2'}$ = 4.0 Hz), 5.84 (d, 1H, H-5, $J_{5,6}$ = 8.4 Hz), 4.52 (dd, 1H, H-5', J = 3.2, J = 1.2 Hz), 4.33-4.24 (m, 4H, H-2', H-3', H-4', H-6'); LR-ESIMS 340.0 [(M+Na)$^+$].

Methyl 6-(*N*-3-(*S*)-*tert*-Butoxycarbonylamino-*tert*-butoxycarbonylpropyl)amino-1-(uracil-1-yl)-6-deoxy-2,3-*O*-isopropylidene-β-D-*glycero*-L-*talo*-heptofuranuronate

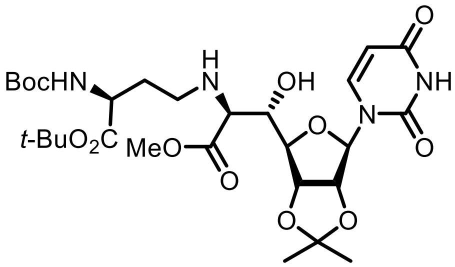

Chemical Formula: $C_{28}H_{44}N_4O_{12}$
Exact Mass: 628.30
Molecular Weight: 628.68

A suspension of methyl 6-benzyloxycarbonylamino-1-(3-benzyloxymethyluracil-1-yl)-6-deoxy-2,3-*O*-isopropylidene-β-D-*glycero*-L-*talo*-heptofuranuronate[1] (20.0 mg, 39.6 μmol) and Pd black (10 mg) in MeOH was stirred at room temperature under H_2 gas. After 1 h, the insoluble was filtered off through Celite pad, and the filtrate was concentrated *in vacuo*. The resulting amine and *tert*-butyl 4-oxo-2-(*S*)-*tert*-butoxycarbonylaminobutanoate (11.0 mg, 40.0 μmol), which was prepared from *tert*-butyl 2-(*S*)-*tert*-butoxycarbonylamino-4-pentenoate by ozonolysis, in CH_2Cl_2 was treated with AcOH (11.5 μL, 200 μmol) and $NaBH(OAc)_3$ (42.4 mg, 200 μmol) at room temperature for 21 h. The reaction was quenched with *sat. aq.* $NaHCO_3$, and the resulting mixture was extracted with EtOAc. The organic layer was washed with *sat. aq.* $NaHCO_3$ and brine, dried over Na_2SO_4, filtered and concentrated *in vacuo*. The residue was purified by silica gel column chromatography (ϕ1.0×5 cm; hexane/EtOAc = 2/1 → 1/1 → 1/2, then ϕ1×5.0 cm; $MeOH/CHCl_3$ = 0% → 1%) to afford the title compound (6.2 mg, 25%) as a colorless oil.

^1H NMR ($CDCl_3$, 400 MHz) δ 7.92 (d, 1H, H-6, $J_{6,5}$ = 8.2 Hz), 6.16 (d, 1H, H-1', $J_{1',2'}$ = 3.6 Hz), 5.78 (d, 1H, H-5, $J_{5,6}$ = 8.2 Hz), 5.21 (d, 1H, BocN*H*, *J* = 7.3 Hz), 4.91 (br d, 1H, H-3', *J* = 4.5 Hz), 4.73 (t, 1H, H-2', *J* = 4.8 Hz), 4.32-4.25 (m, 1H, Dab-α-C*H*), 4.25 (br s, 1H, H-4'), 3.78 (s, 3H, OMe), 3.66 (br d, 1H, H-5', *J* = 8.6 Hz), 3.34 (d, 1H, H-6', *J* = 9.1 Hz), 2.84 (dt, 1H, Dab-γ-CH_2, *J* = 11.8, *J* = 5.9 Hz), 2.56-2.49 (m, 1H, Dab-γ-CH_2), 2.05-1.96 (m, H, Dab-β-CH_2), 1.86-1.76 (m, 1H, Dab-β-CH_2), 1.61 (s, 3H, isopropylidene-CH_3), 1.47 (s, 9H, *t*-Bu), 1.45 (s, 9H, *t*-Bu), 1.35 (s, 3H, isopropylidene-CH_3); HR-ESIMS calc. $C_{28}H_{45}N_4O_{12}$ 629.3028, detected 629.3031.

6-(*N*-3-(*S*)-*tert*-Butoxycarbonylamino-*tert*-butoxycarbonylpropyl)amino-1-(uracil-1-yl)-6-deoxy-2,3-*O*-isopropylidene-β-D-*glycero*-L-*talo*-heptofuranuroic acid

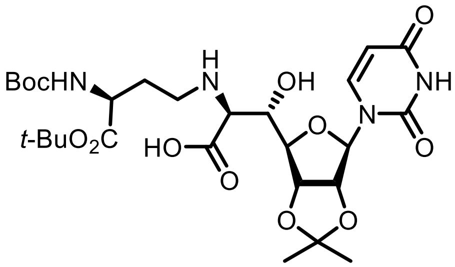

Chemical Formula: $C_{27}H_{42}N_4O_{12}$
Exact Mass: 614.28
Molecular Weight: 614.65

A suspension of methyl 6-(*N*-3-(*S*)-*tert*-butoxycarbonylamino-*tert*-butoxycarbonylpropyl)amino-1-(uracil-1-yl)-6-deoxy-2,3-*O*-isopropylidene-β-D-*glycero*-L-*talo*-heptofuranuronate (6.2 mg, 9.86 μmol) and Me_3SnOH (9.0 mg, 50.0 μmol) in 1,2-dichloroethane (1 mL) was heated to 90 °C and stirred for 29 h. The reaction mixture was cooled and concentrated *in vacuo*. The residue was purified by silica gel column chromatography (ϕ0.6×4 cm; MeOH/$CHCl_3$ = 0% → 5% → 10%) to afford the title compound (4.3 mg, 71%) as a white solid.

^{1}H NMR (DMSO-d_6, 400 MHz) δ 7.83 (d, 1H, H-6, $J_{6,5}$ = 8.1 Hz), 7.18 (d, 1H, BocN*H*, *J* = 7.6 Hz), 5.87 (s, 1H, H-1'), 5.62 (d, 1H, H-5, $J_{5,6}$ = 8.1 Hz), 4.84-4.75 (m, 2H, H-2', H-3'), 4.26 (br s, 1H, H-4'), 3.92-3.79 (m, 2H, H-5', Dab-α-C*H*), 3.15 (br s, 1H, H-6'), 2.91-2.60 (m, 2H, Dab-γ-CH_2), 1.93-1.74 (m, 2H, Dab-β-CH_2), 1.47 (s, 3H, isopropylidene-CH_3), 1.37 (s, 9H, *t*-Bu), 1.27 (s, 3H, isopropylidene-CH_3); HR-ESIMS calc. $C_{27}H_{43}N_4O_{12}$ 615.2872, detected 615.2902.

6-(*N*-3-(*S*)-*tert*-Butoxycarbonylamino-*tert*-butoxycarbonylpropyl)amino-1-(uracil-1-yl)-6-deoxy-2,3-*O*-isopropylidene-β-D-*glycero*-L-*talo*-heptofuranuroic acid (**11**)

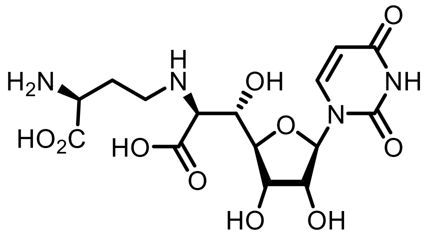

Chemical Formula: $C_{15}H_{22}N_4O_{10}$
Exact Mass: 418.13
Molecular Weight: 418.36

6-(*N*-3-(*S*)-*tert*-Butoxycarbonylamino-*tert*-butoxycarbonylpropyl)amino-1-(uracil-1-yl)-6-deoxy-2,3-*O*-isopropylidene-β-D-*glycero*-L-*talo*-heptofuranuroic acid (4.3 mg, 7.00 μmol) was treated with 80% *aq.* TFA (1 mL) at room temperature for 22 h. The reaction mixture was concentrated *in vacuo*, and the residue was co-evaporated with toluene three times. The resulting solid was washed with Et_2O to afford **11** (3.9 mg, 86% as 2TFA salt) as a white solid.

^1H NMR (D_2O, 400 MHz) δ 7.99 (d, 1H, H-6, $J_{6,5}$ = 8.1 Hz), 5.88 (d, 1H, H-1', $J_{1',2'}$ = 4.0 Hz), 5.85 (d, 1H, H-5, $J_{5,6}$ = 8.1 Hz), 4.36 (t, 1H, H-2', J = 4.9 Hz), 4.29-4.25 (m, 2H, H-3', H-5'), 4.22 (dd, 1H, H-4', J = 5.4, J = 1.8 Hz), 3.92-3.88 (m, 2H, H-6', Dab-α-C*H*), 3.37-3.22 (m, 2H, Dab-γ-CH_2), 2.32-2.18 (m, 2H, Dab-β-CH_2); HR-ESIMS calc. $C_{15}H_{23}N_4O_{10}$ 419.1409, detected 419.1383.

Figures

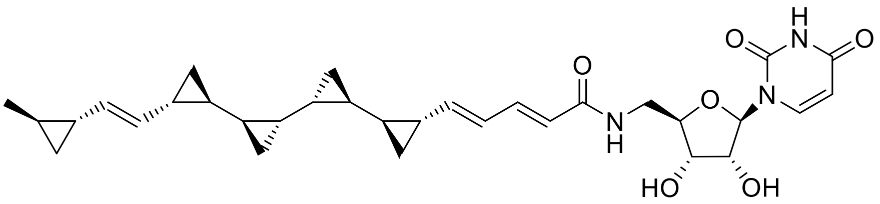

Jawasamycin

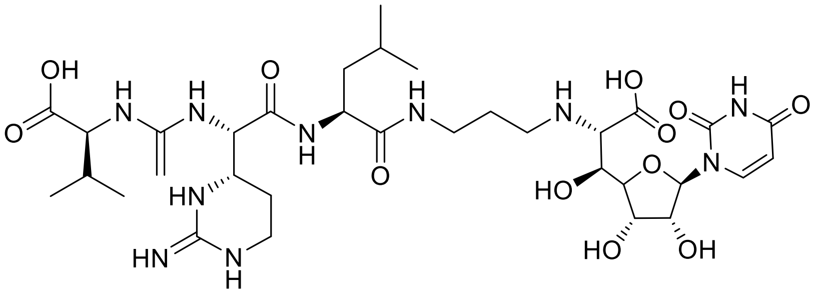

Muraymycin D4

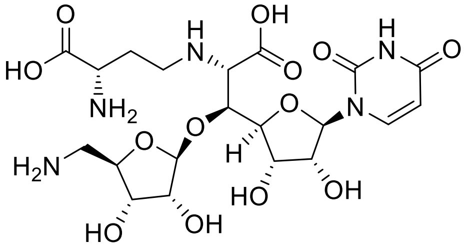

16

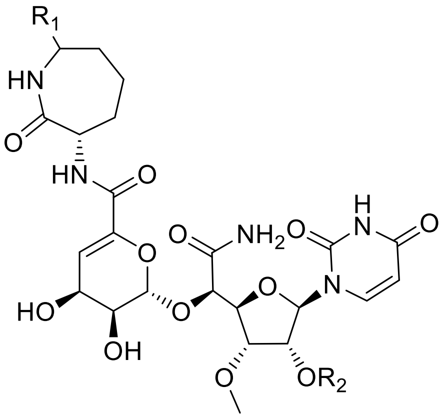

Capuramycin $R_1 = R_2 = H$
A-500359 A $R_1 = CH_3$, $R_2 = H$
A-503083 A $R_1 = CH_3$, $R_2 = CONH_2$

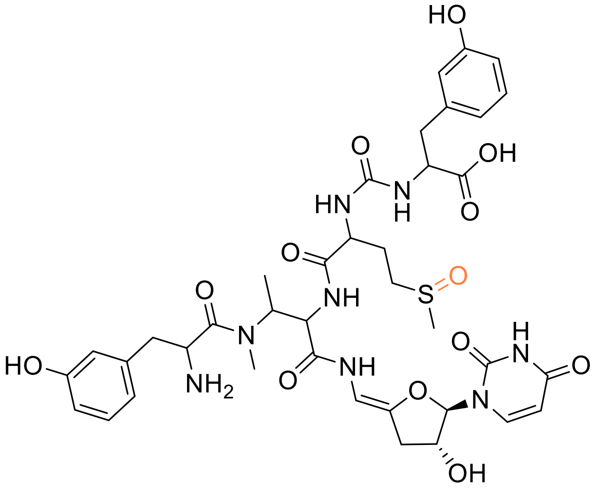

N-acetylmureidomycin E

Supplementary Figure 1. Chemical structures of MraY inhibitors referred to in the main text.

a

Protein (MO)	Cpz15	LipL	LpmM	Jaw7	Mur16	Cpr19	CapA	Orf7
Cpz15		86/90	82/89	79/86	45/58	38/53	35/52	36/52
LipL			80/86	77/85	46/56	39/52	36/50	37/51
LpmM				80/85	43/56	37/52	35/51	38/52
Jaw7					43/53	37/51	37/51	36/50
Mur16						53/67	52/65	52/66
Cpr19							76/88	75/88
CapA								84/91
Orf7								

b

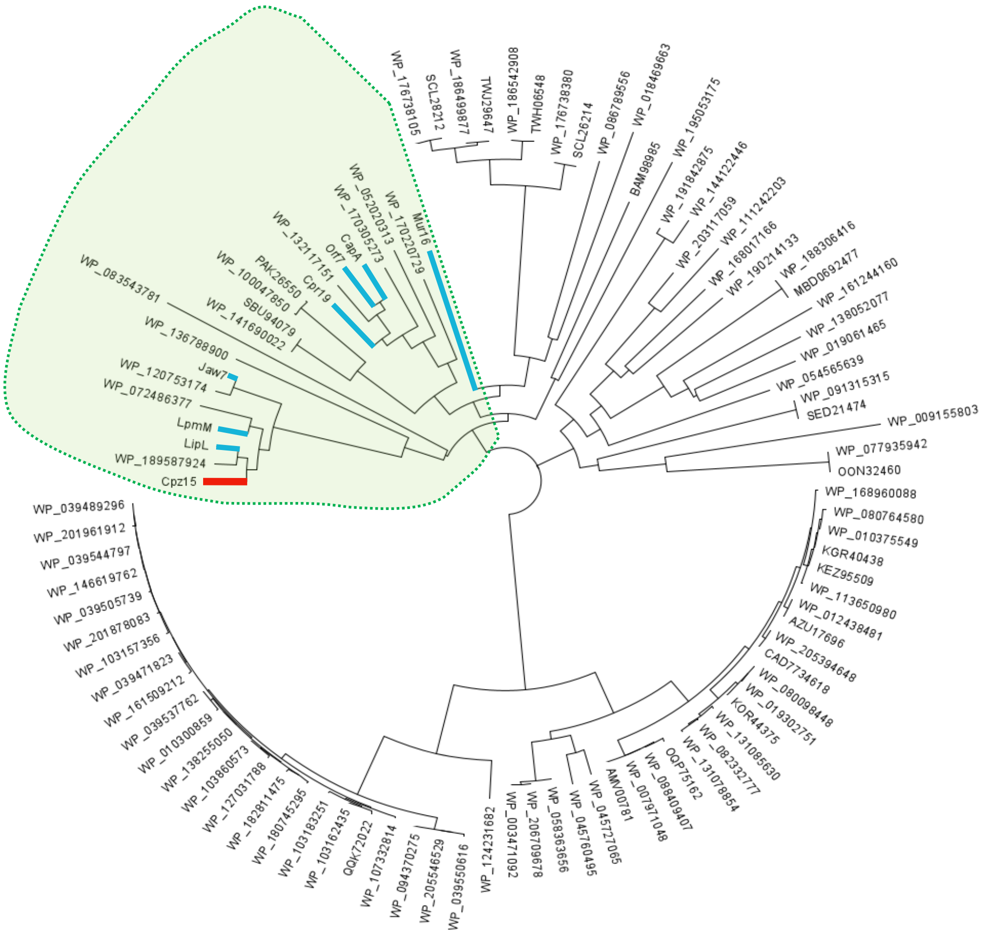

Supplementary Figure 2. Bioinformatic analysis of Cpz15. a. The sequence identity and similarity of Cpz15 against selected homologous proteins were showed in percentage. The Cpz15 shows higher similarity and identity with LipM, LpmM and Jaw7. **b**. The phylogenetic analysis of Cpz15. Cpz15, oxidative dephosphorylase, was functionally assigned in this research. Mur16 is a dioxygenase involved in the biosynthesis of compound **6** and **7**. The depicted tree and the table (a) contain representative proteins from sequence alignments including: LipL, UMP dioxygenase; LpmM, dioxygenase; Jaw7 and Cpr19, UMP dioxygenase; Orf7, putative dioxygenase; CapA, putative nonheme oxygenase. Sequences were aligned with Geneious prime of Blosum45 and assembled by the Jukes-Cantor Genetic Distance model with the Neighbor-Joining Tree Build method. The phylogenetic tree was resampled using the Bootstrap method with 100 replicates.

a

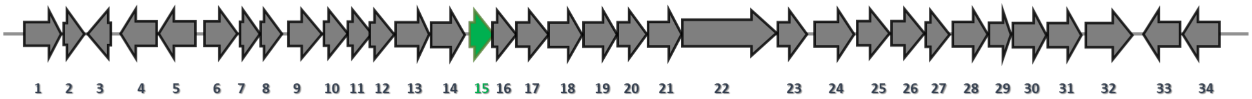

b

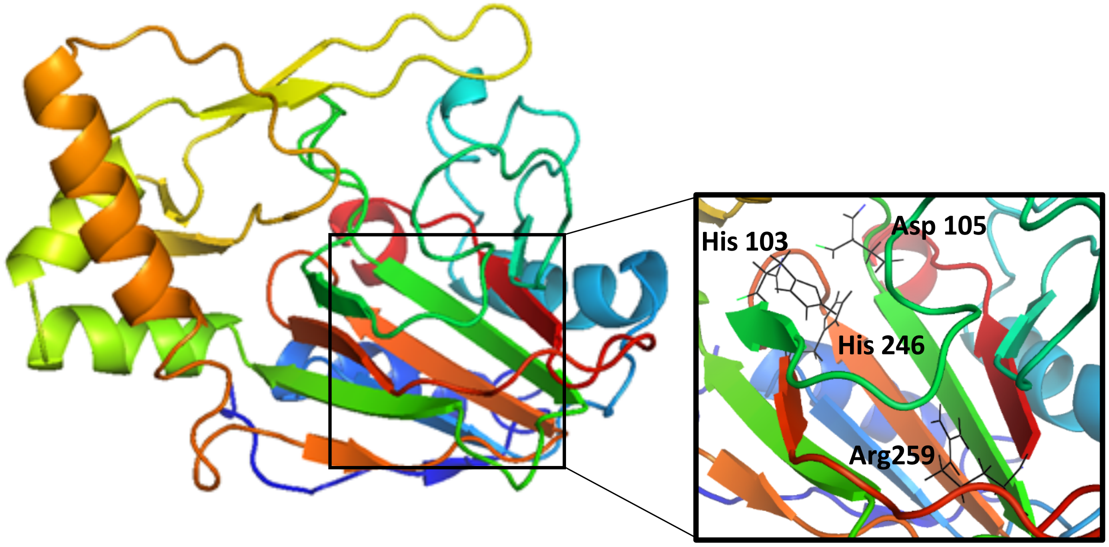

Supplementary Figure 3. Homology modeling of Cpz15. **a**, The location of the Cpz15 in the *cpz* gene cluster. **b**, The i-TASSER homolgy modeling of Cpz15. The iron binding motif residues H103, D105 and H246 are in a facial-triad geometry, and the R259 was identified as a C-5 stabilizer amino acid.

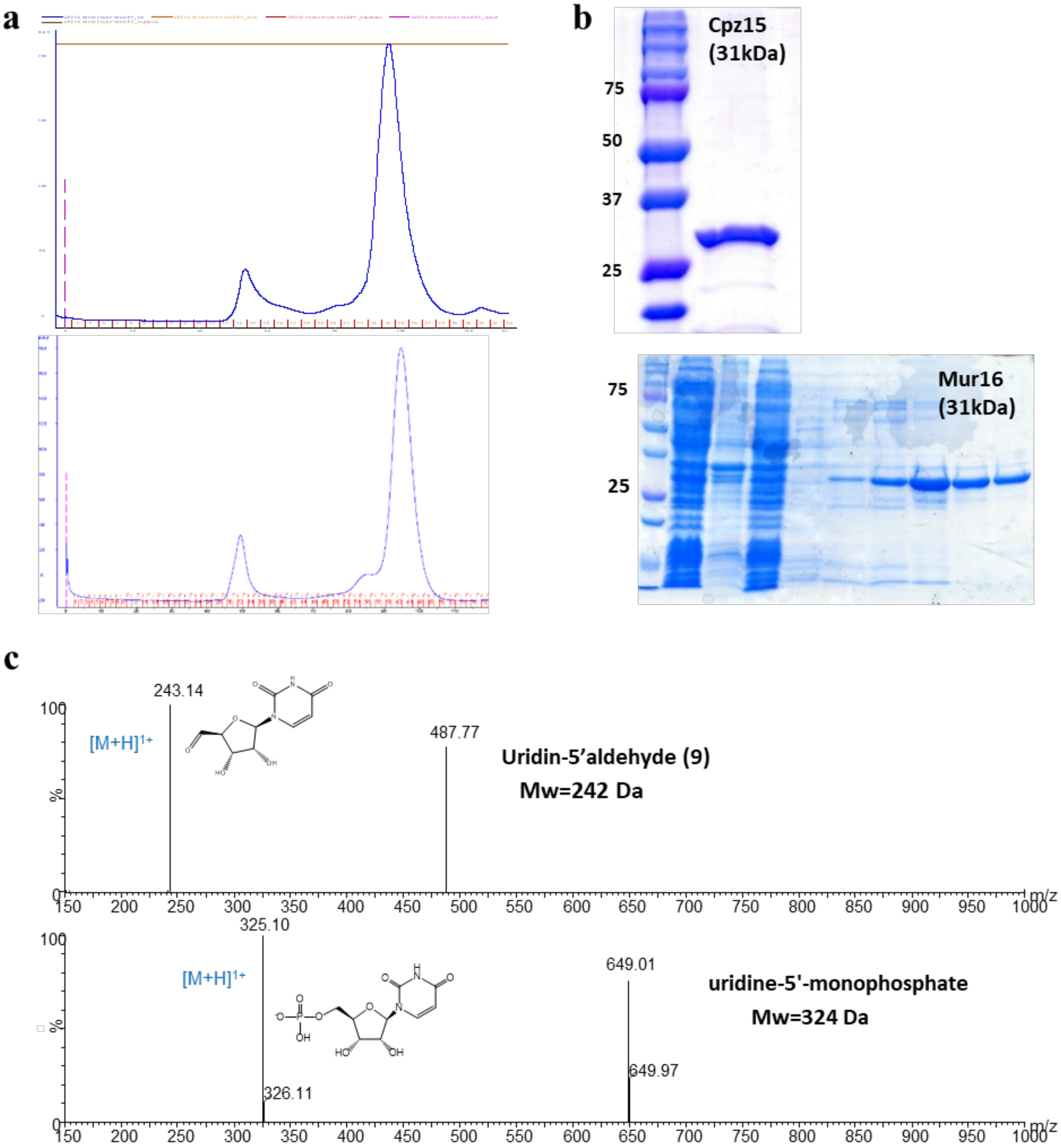

Supplementary Figure 4. SDS-PAGE, FPLC and ESI-MS analysis of Cpz15 and Mur16 reactions. a, Fast Protein Liquid Chromatography (FPLC) profile of Cpz15 (up) and Mur16 (down). **b,** SDS-PAGEs of pure His$_6$-Cpz15 and His$_6$-Mur16. **c,** ESI-MS of UMP and compound **9**. Calc. for $C_9H_{14}N_2O_9P$ (UMP) expected $(M+H)^+$ ion at *m/z* = 324 and calc. for $C_9H_{10}N_2O_6$ (**9**) expected $(M+H)^+$ ion at *m/z* = 243.

a

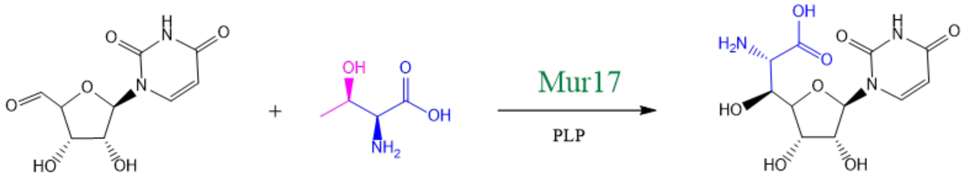

b

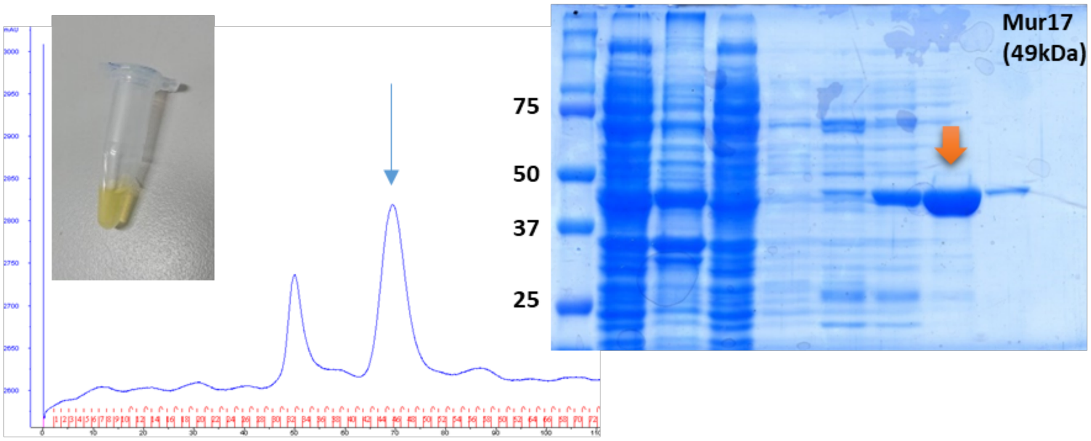

c

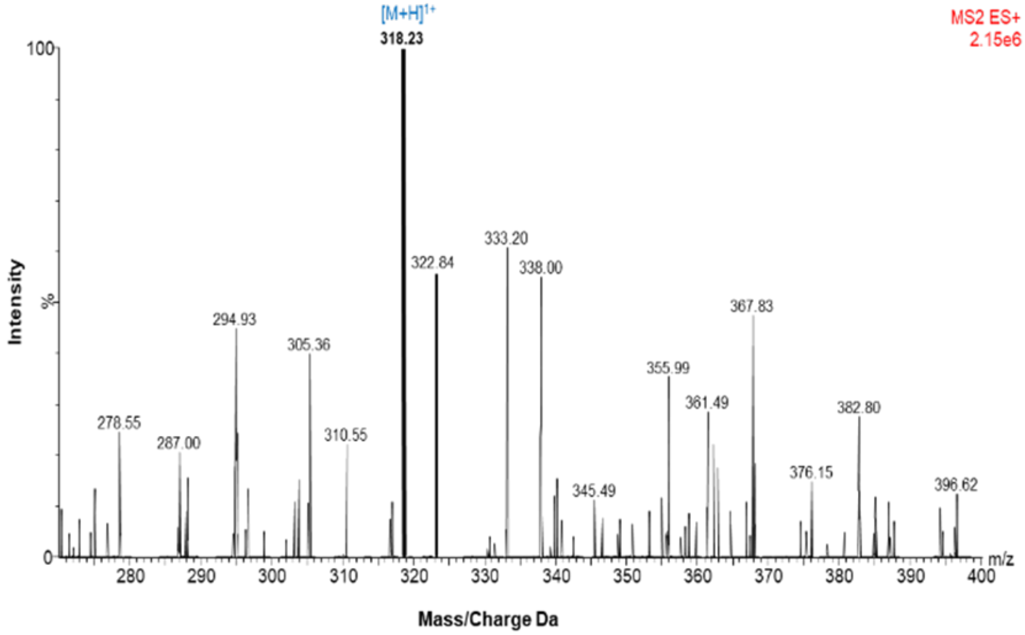

Supplementary Figure 5. Functional assignment of Mur17. a, Chemical reaction catalyzed by Mur17. **b,** Fast Protein Liquid Chromatography (FPLC) profile of Mur17 and SDS-PAGE of purified His_6-Mur17 (Due to the PLP, the color of protein is yellow). **c,** ESI-MS of compounds **10**. calc. for $C_{11}H_{15}N_3O_8$ (**10**) expected $(M+H)^+$ ion at *m/z* = 317.25.

a

Protein (BH)	Cpz10	LipG	LpmH	Mra18	SphK
Cpz10		91/94	87/93	84/91	47/66
LipG			83/92	80/90	46/66
LpmH				83/91	47/65
Mra18					46/67
SphK					

b

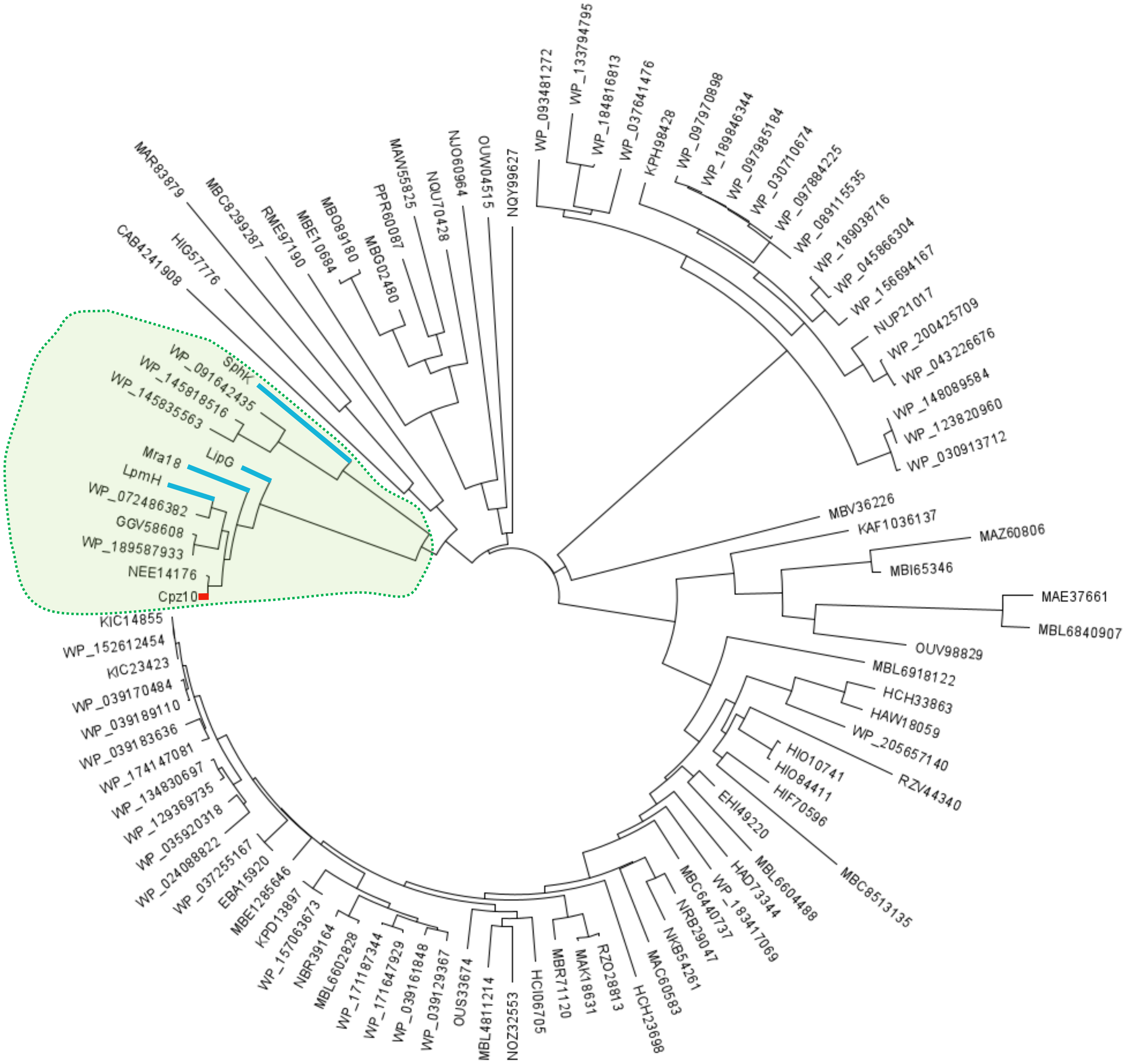

c

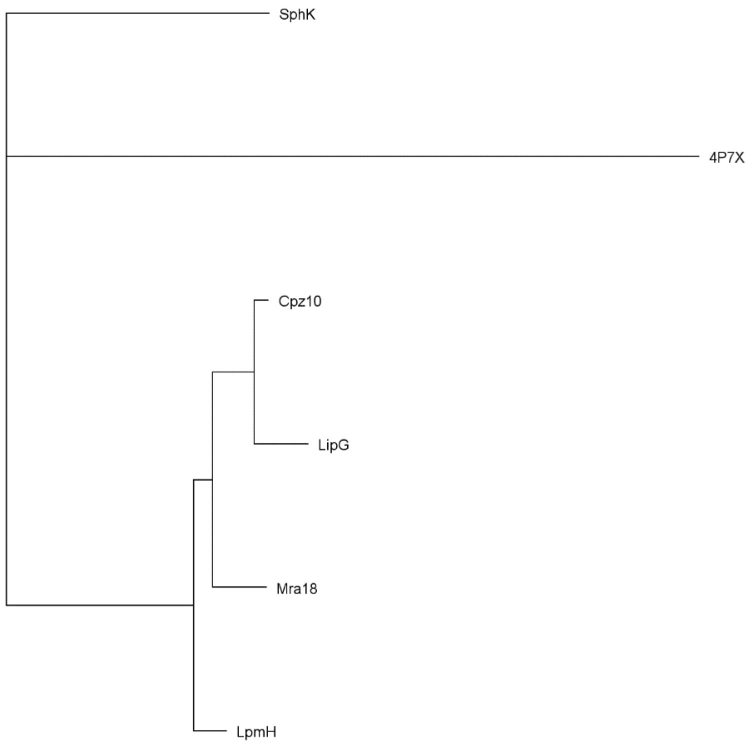

Supplementary Figure 6. Bioinformatic analysis of Cpz10. a, The sequence identity and similarity of Cpz10 and other homologous proteins were showed in percentage. The Cpz15 has higher similarity and identity with LipG, LpmH and Mra18 respectively. **b**, Phylogenetic analysis of Cpz10. Cpz10 was functionally assigned as a β-hydroxylase in this research. LipG and LpmH are putative β-hydroxylases involved in the biosynthesis compound **3**. The depicted tree and the table (a) contain representative proteins from sequence alignments including: Mra18, putative β-hydroxylase and SphK, putative β-hydroxylase. **c,** Phylogenetic analysis of Cpz10 and selective nucleoside antibiotics homologous enzymes with the Hyps enzyme (PDB code. 4P7X) suggested they are structurally close to each other. Sequences were aligned with Geneious prime of Blosum45 and assembled by the Jukes-Cantor Genetic Distance model with the Neighbor-Joining Tree Build method. The phylogenetic tree was resampled using the Bootstrap method with 100 replicates.

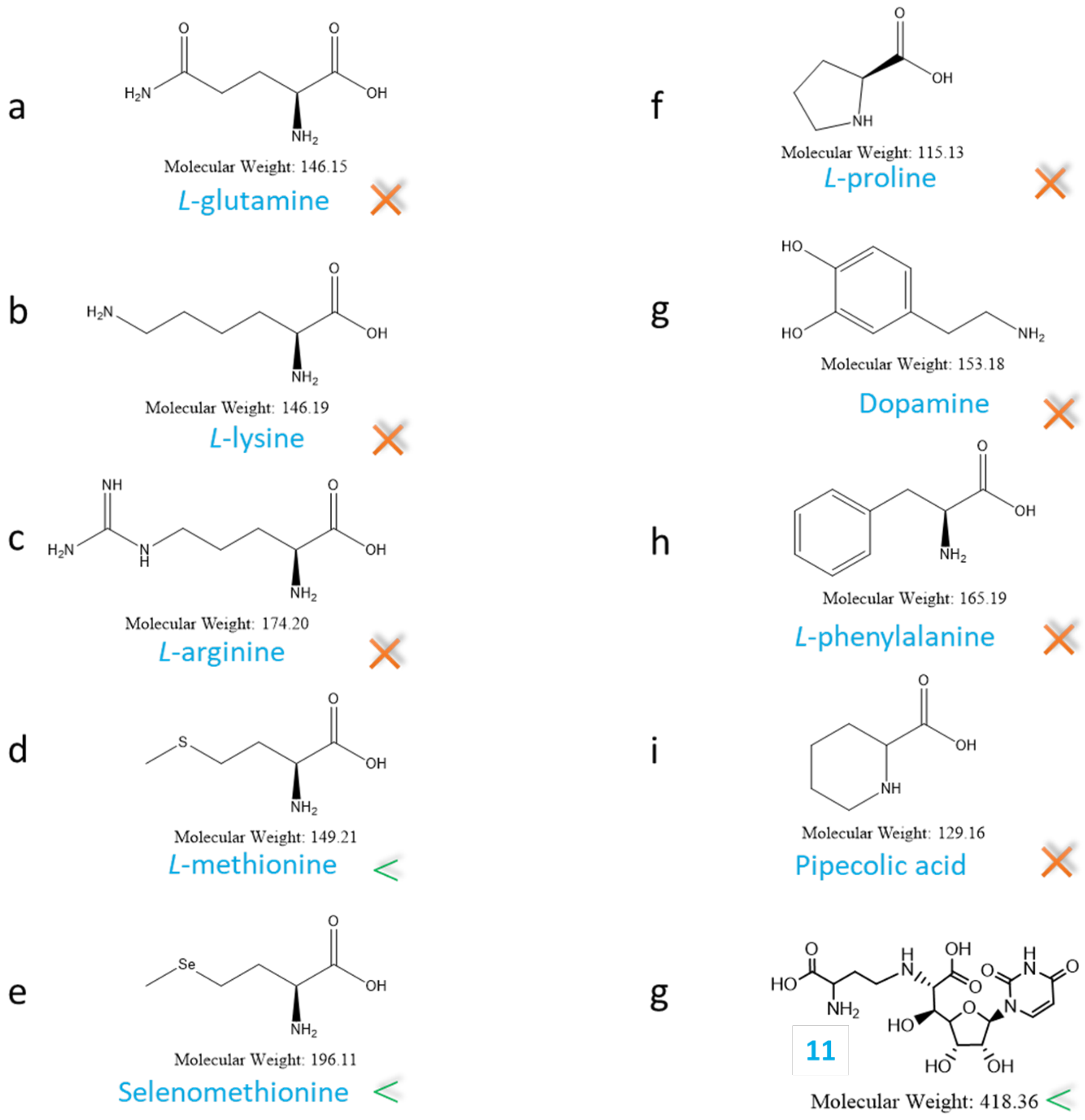

Supplementary Figure 7. Chemical structures of possible substrates for Cpz10 enzymatic reactions. Out of more than 20 compounds (above stuctures are as examples) plus SAM (*S*-adenosyl methionine), only three compounds can react with Cpz10; *L*-methionine, selenomethionine and compound **11.**

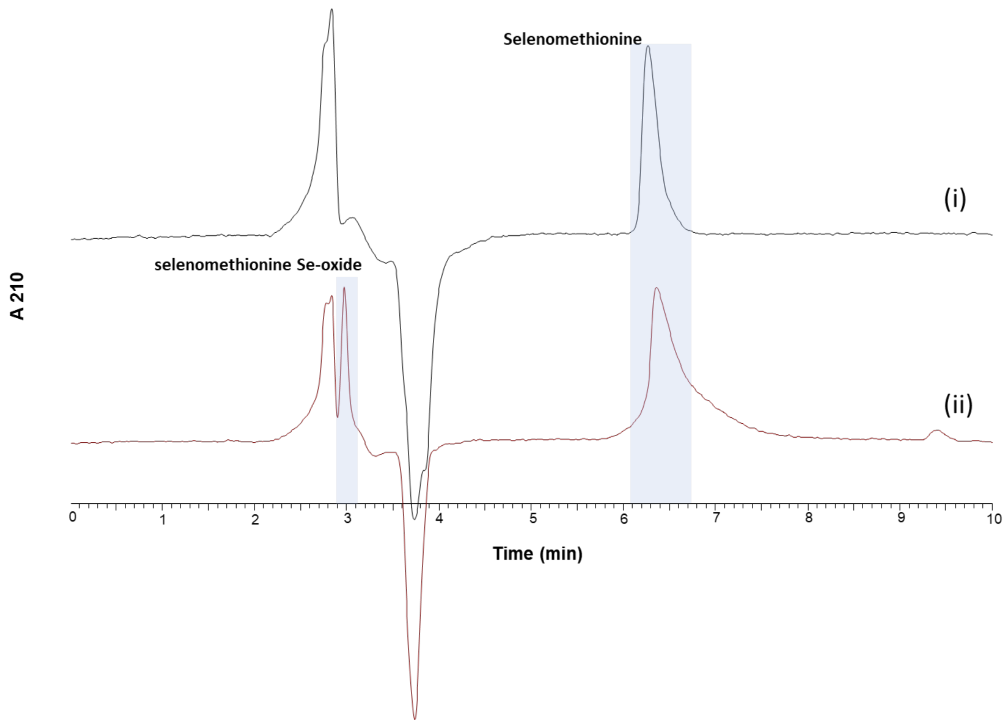

Supplementary Figure 8. The chemical reaction catalyzed by Cpz10 and selenomethionine. HPLC traces of (i) control, selenomethionine, without enzyme and cofactors and (ii) reaction catalyzed by Cpz10 starting from selenomethionine, $FeSO_4$, ascorbic acid and αKG. Reaction product is the compound **Se-oxide**. A_{210} means the UV absorbance at wavelength of 210 nm.

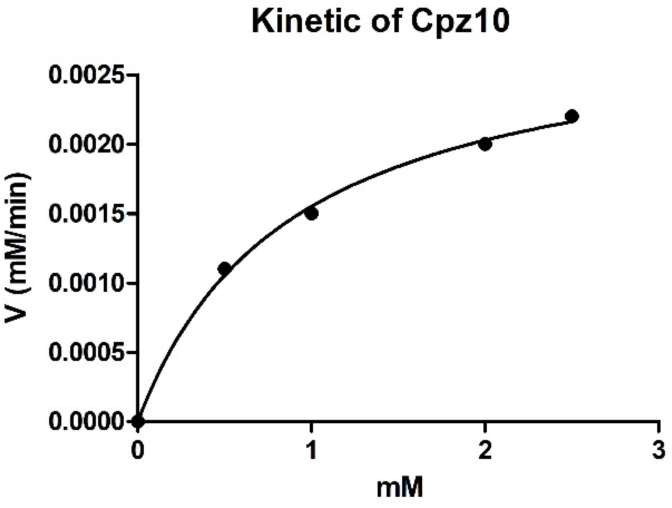

Supplementary figure 9. Steady state kinetics of Cpz10.

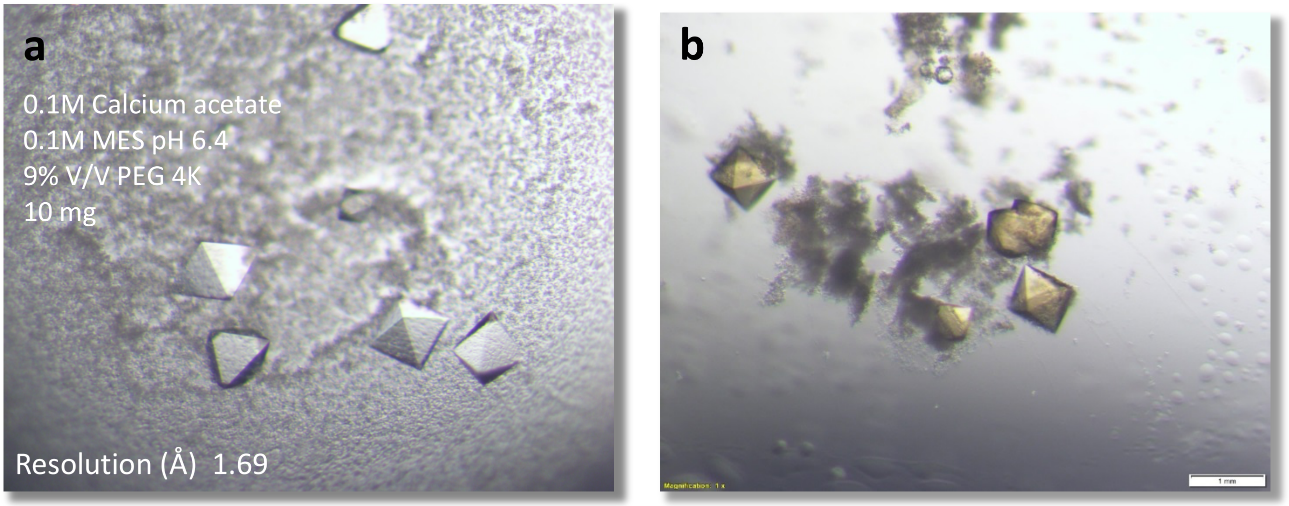

Supplementary Figure 10. The crystals of Cpz10. a, The apo form crystal shows in octahedron shape. **b,** The color of crystal was changed after soaking with compound **11**, αKG and Fe^{2+} for overnight.

a

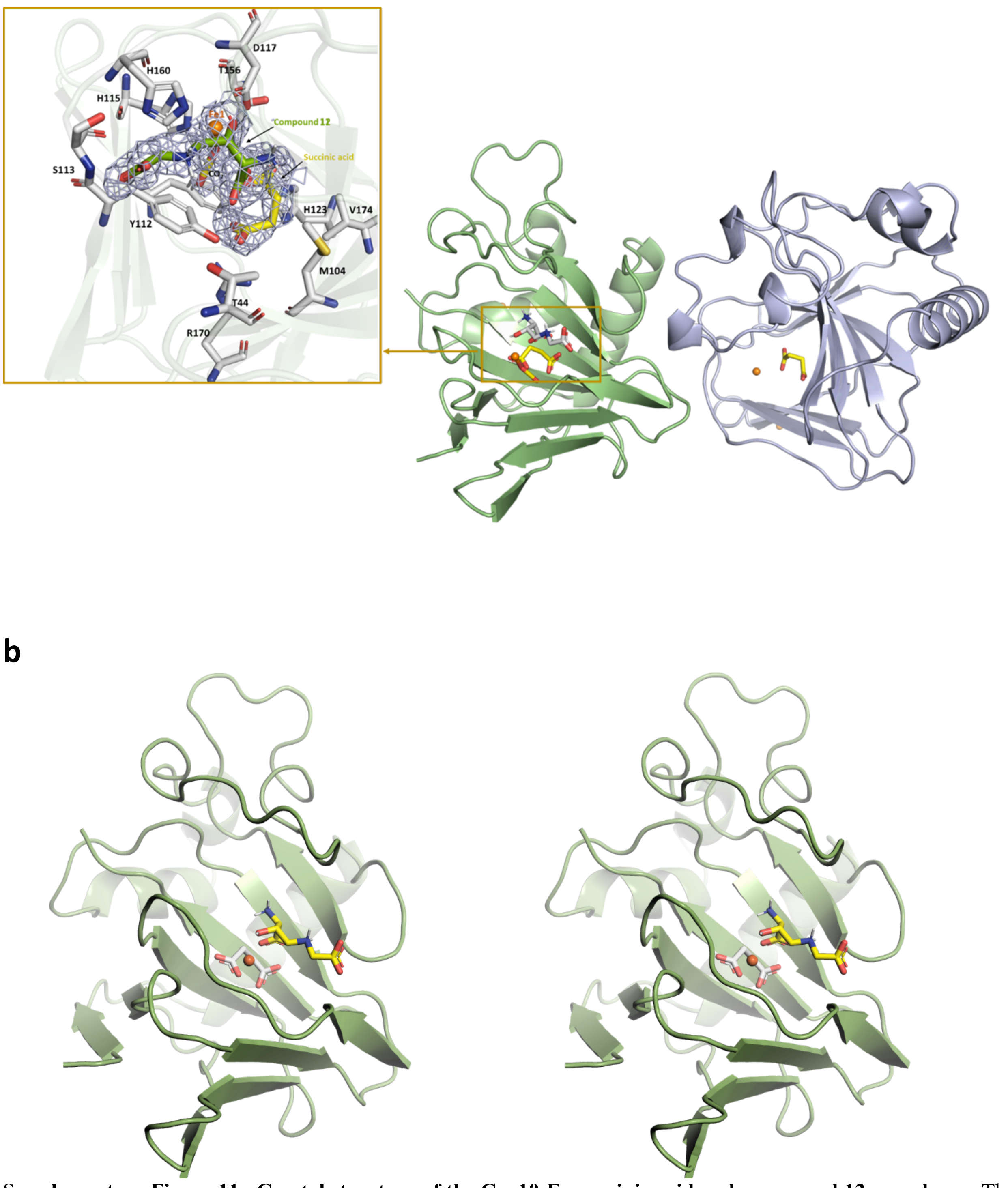

b

Supplementary Figure 11. Crystal structure of the Cpz10-Fe-succinic acid and compound 12 complex. **a**. The protein asymmetric unit containing two polypeptide chains in dimeric manner. Focal figure shows the $2f_o$-f_c electron density map (contour 1.0 s) of the 2-amino-3-hydroxybutanoic acid moiety of compound **12**, CO_2, succinic acid and Fe^{2+}. **b**. stereo view of active site. Structural elucidation was done by PyMol (64-bit) software.

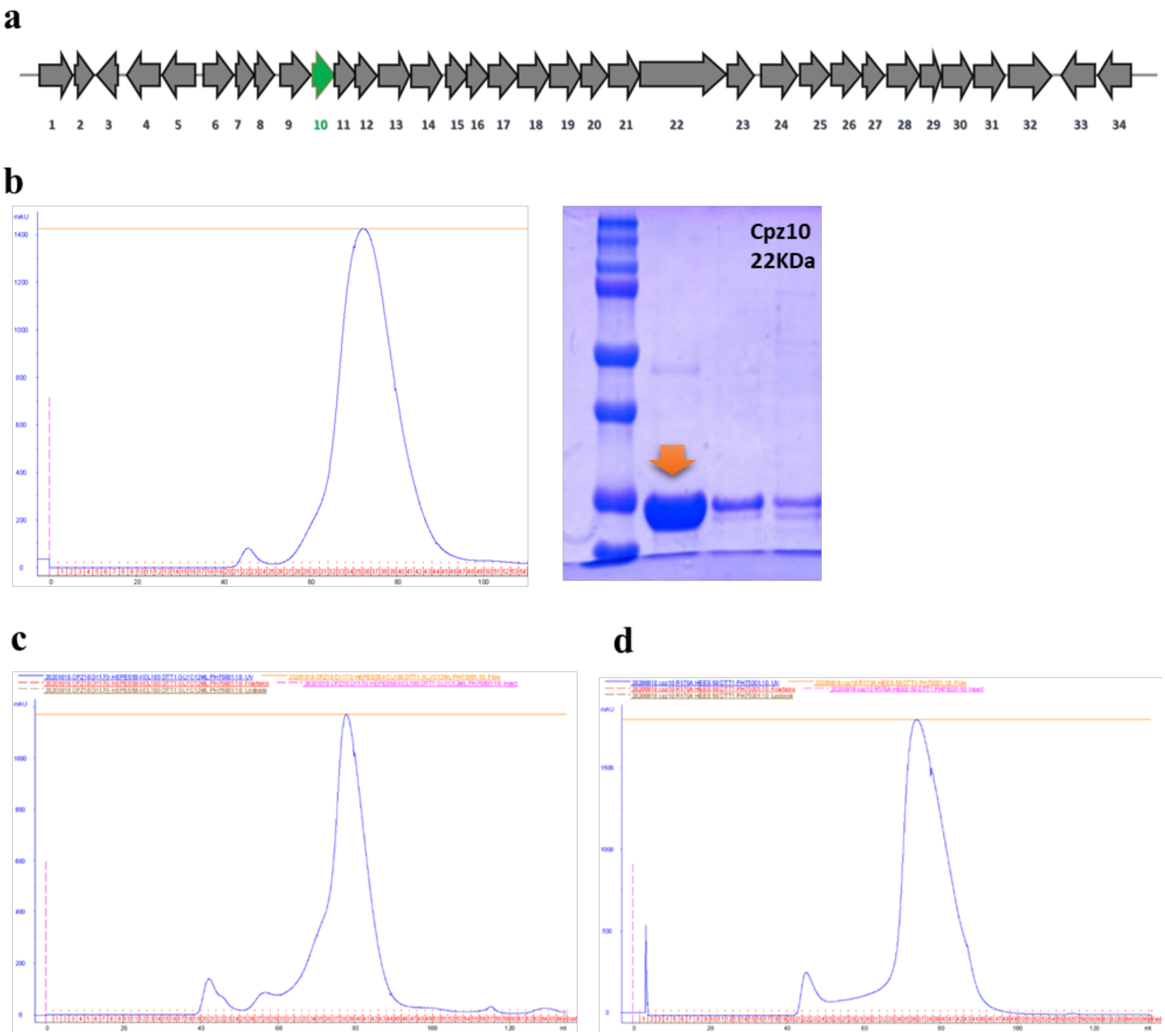

Supplementary Figure 12. SDS-PAGE, FPLC profile of Cpz10, Cpz10-D117G and Cpz10-R170A. a, The location of the Cpz10 in the *cpz* gene cluster. **b,** Fast Protein Liquid Chromatography (FPLC) profile of Cpz10 and SDS-PAGE of pure His$_6$-Cpz10. **c,** Fast Protein Liquid Chromatography (FPLC) profile of Cpz10-D117G. **d,** Fast Protein Liquid Chromatography (FPLC) profile of Cpz10-R170A.

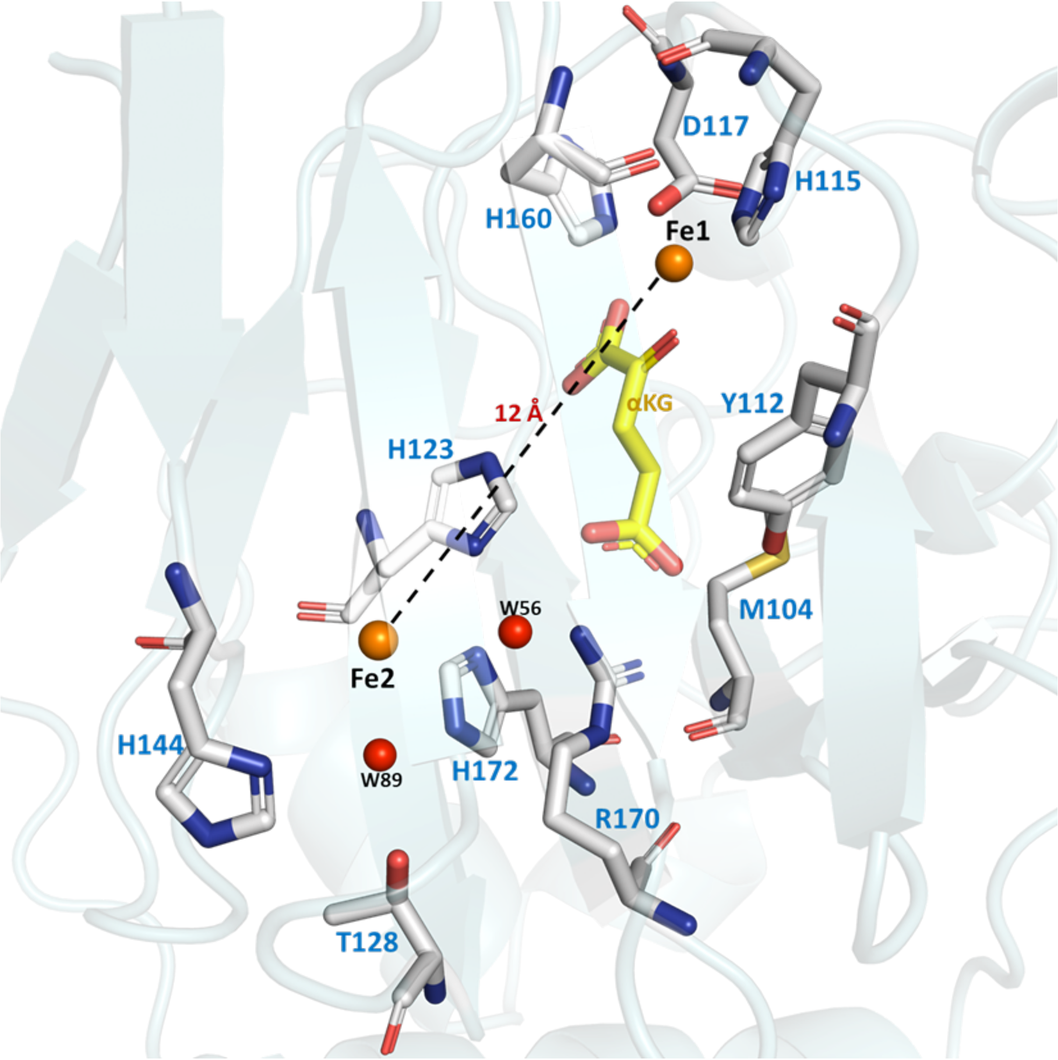

Supplementary Figure 13. The complex structure of Cpz10 with αKG and two Fe ions. Residues within 4 Å of αKG and two Fe ions are identified. The distance between two iron ions in structure is close to 12 Å. Amino acids demonstrated in this figure are conserved in Cpz10 and its homologs. Structural elucidation was done by PyMol (64-bit) software.

```
Cpz10        PGRR ---------------------------------AC -V       --      D
LipG         AQGS SPFHRVGIGPSVPVEEEHGEWRRRCAAAPGRRKAC -V       --      G
SphK         MNHV E--------------------------------VS TRP  VG V--P G  VA
LpmH         -------------------------------------------- V      --V     V
Mra18        -------------------------------------------- P     I--  N   D
4P7X         ASWS ---------------------------------PQ -EK A    ILGV Q  QR

Cpz10             VE     --------------------               E
LipG          Q   TN     --------------------
SphK            ET       --------------------     N Q     I   TE      S Y
LpmH                     --------------------                S
Mra18         C          --------------------               E A   H   W
4P7X          T D  V AKSNFSSEYSDFACGRWEFCMLRNQSGKQEEQR V HET  LAT LGQS--

Cpz10                                -       -                        ---
LipG                H                -       -            S           ---
SphK         QEKD AP DA DT NP    G  FET L A-F  QVR -A          SG T        E ---
LpmH                                 -       -              H         ---
Mra18             A        P    G    -       -                      Q ---
4P7X         -------------------LP  N  L NHFDRDS  YA    R SENA IIF   YLELE

Cpz10        ---                                     Q
LipG         ---      A                       S      E
SphK         ---A     D     L     KNE  R  P  G L     E    M   RNR     V
LpmH         ---                  P    N     N       H
Mra18        ---            H          N     N       H
4P7X         GKFI V  V D N KCS--NT ENN F  - R      ASLR   GCFSPTR    VD

Cpz10          ----------------------------------------------------------
LipG           ----------------------------------------------------------
SphK         AA----------------------------------------------------------
LpmH           ----------------------------------------------------------
Mra18          ----------------------------------------------------------
4P7X         I GTRSLEEVAINVEQPSARNATVDTRKEWTDETLESVLGFSEIISEANYREIVAILAKL

Cpz10                ----------------------  SK------------------------
LipG                 ----------------------  P ------------------------
SphK                 ------------------------ ------------------------
LpmH                 ----------------------D QS------------------------
Mra18                ----------------------P Q ------------------------
4P7X                 HFFHKVHCVDMYGWLKEICRRR  PALIEKANSLERFYLIDRAAGEVMTY
```

Supplementary Figure 14a. Sequence alignment of Cpz10 and its homologs. The conserved residues Arg170 and His115, Asp117 and His160 residues which involved in Fe1 and αKG binding site are highlighted in red color. Conserved residues of His172, His144, Thr128, His123, Tyr112 and Met104 which involved in Fe2 binding and electron transferring region are highlighted in blue, light blue, yellow and green.

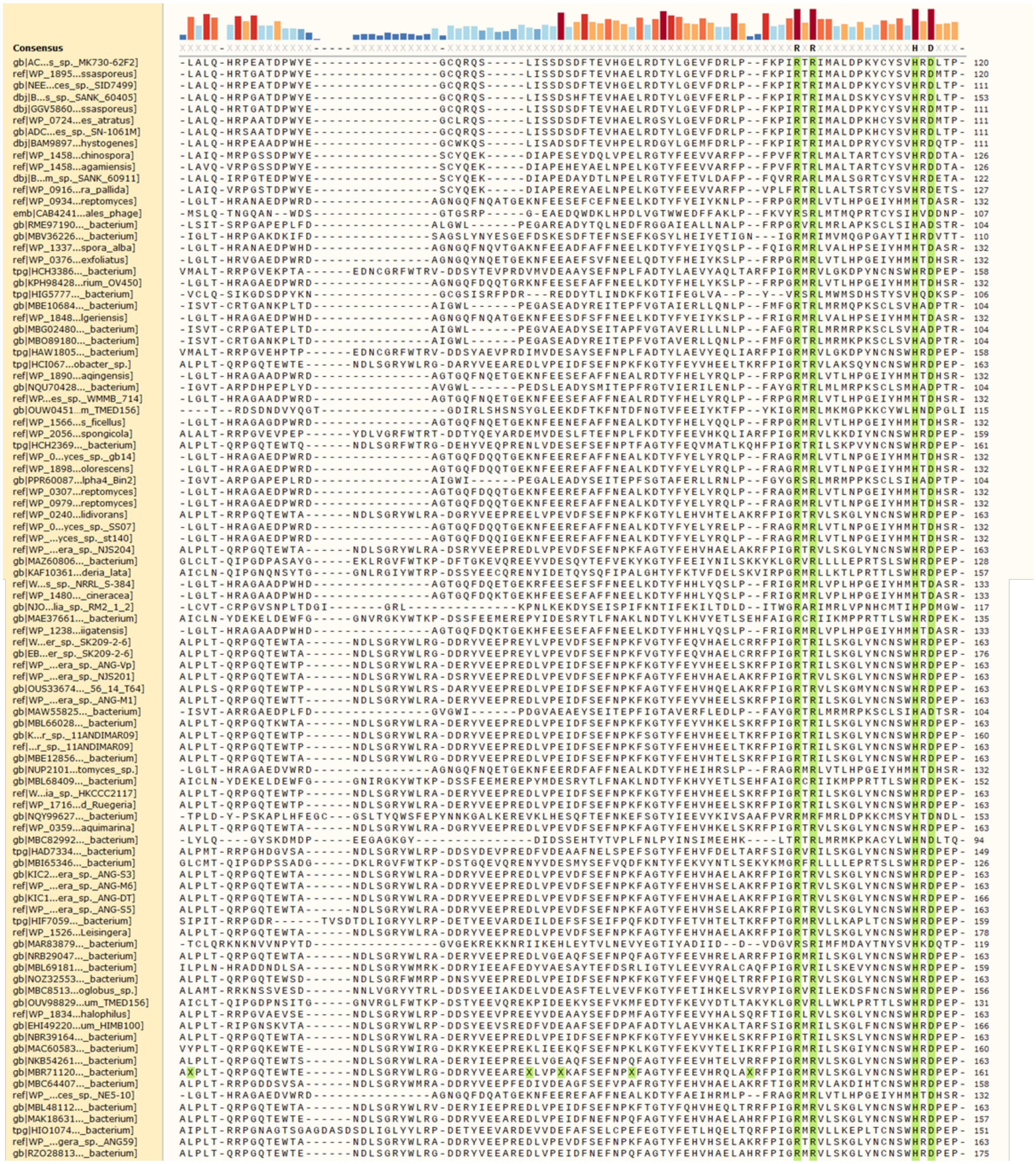

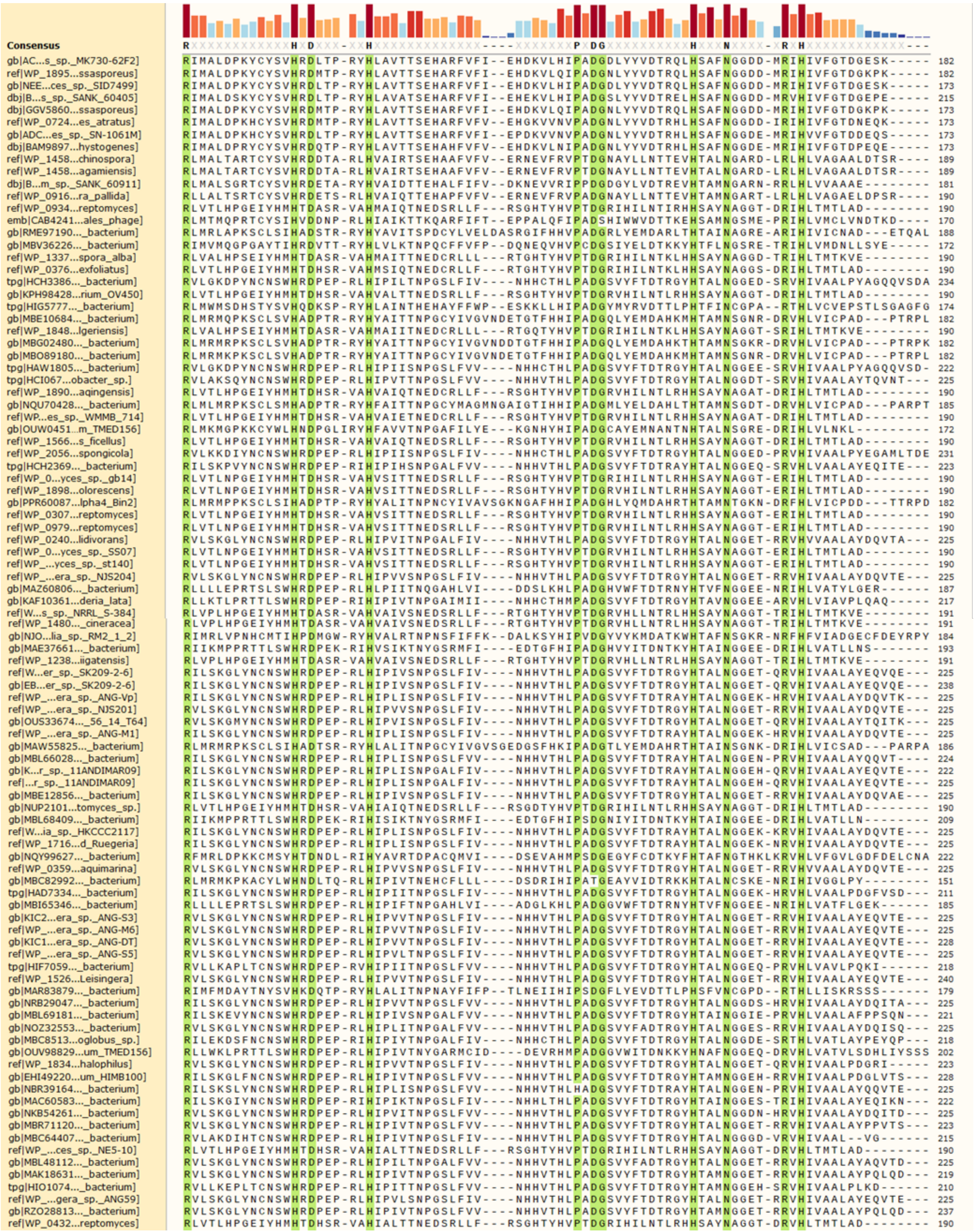

Supplementary Figure 14b. Sequence alignment of Cpz10. Conserved amino acids within 99 proteins are highlighted. Viewed by SnapGene software.

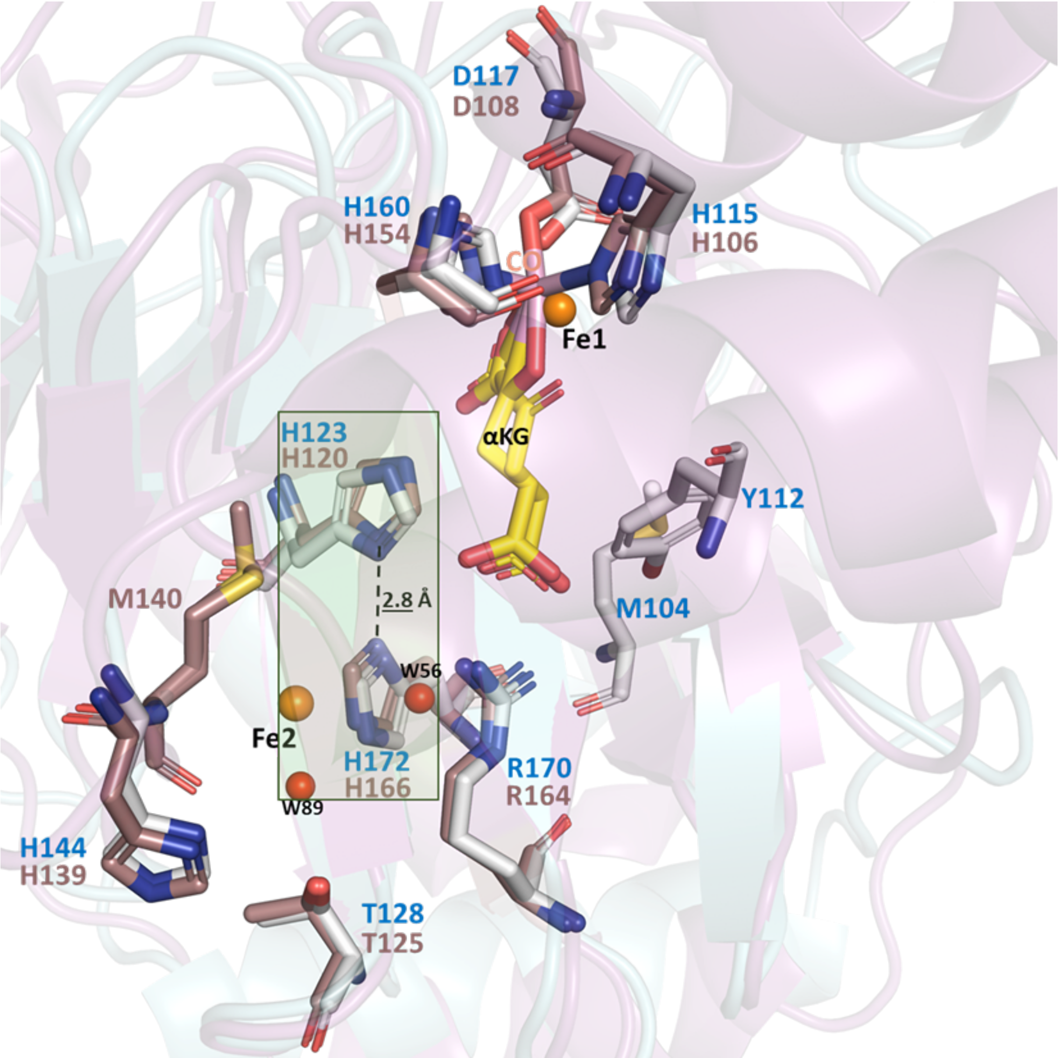

Supplementary Figure 15. Superimposition of two protein structures. Cpz10 complex structure is showed in white color and complex structure of 4P7X is showed in magenta color. Structural superposition of two protein structure shows the conserved amino acids have same position and location in both enzymes. The 4P7X might have same electron transfer function as Cpz10. Structural superposition was done by PyMol (64-bit) software.[2] RMSD: 0.710 (66 to 66 atoms)

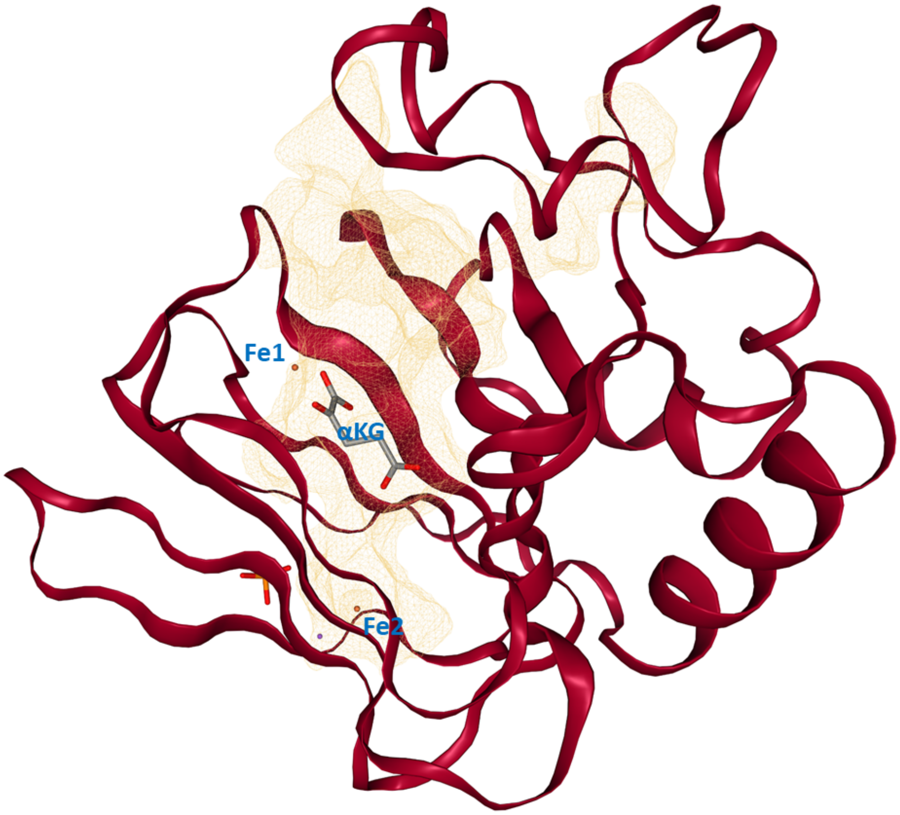

Supplementary Figure 16. Binding site and free space in the Cpz10 structure. The orange mesh space shows the area that Fe2 can move inside the protein**.** Calculated by DoGSiteScorer.[3]

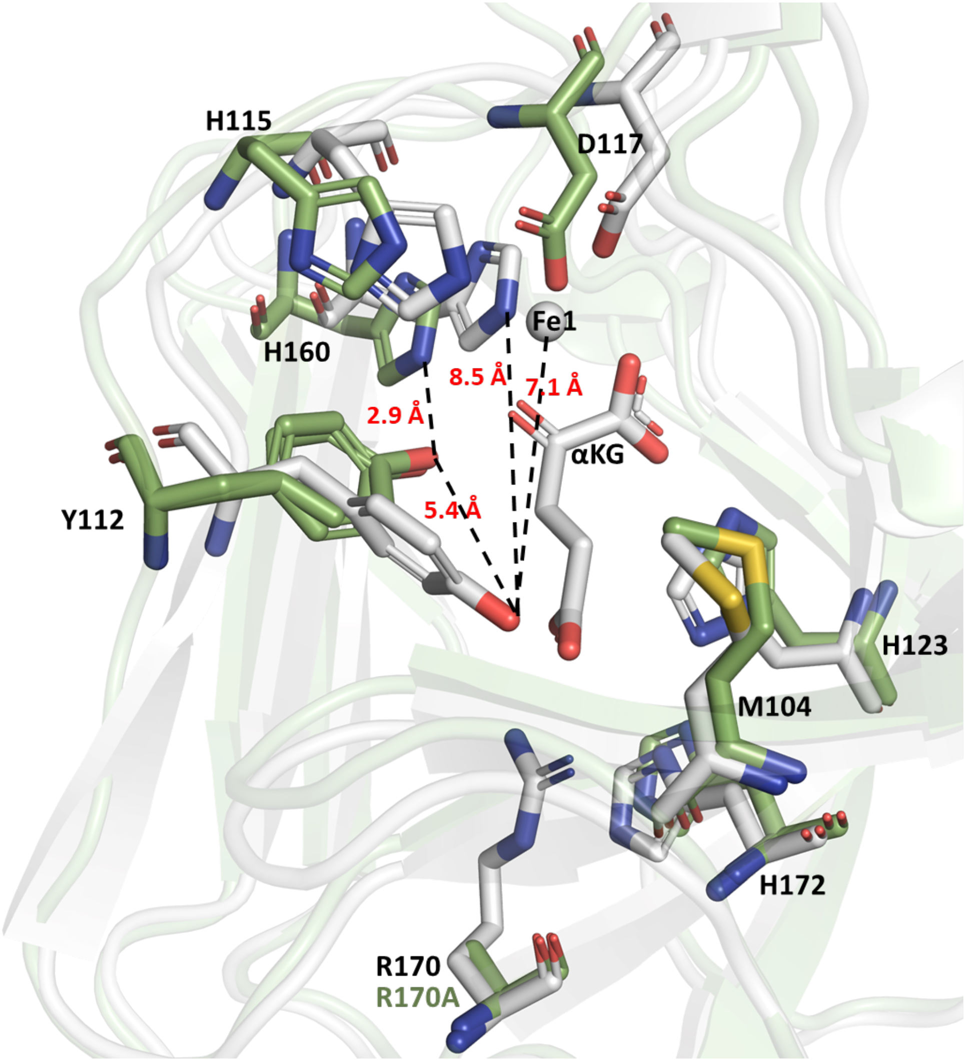

Supplementary Figure 17. Alternative conformation of Tyr112. Distance between Y112, αKG and Nε2 of His160 is 8.5 Å; however, the distance is changed to 2.9 Å in the apo structure. Lime color is the structure of the R170A mutant and the white one is the trinary structure. Structural elucidation was done by PyMol (64-bit) software.

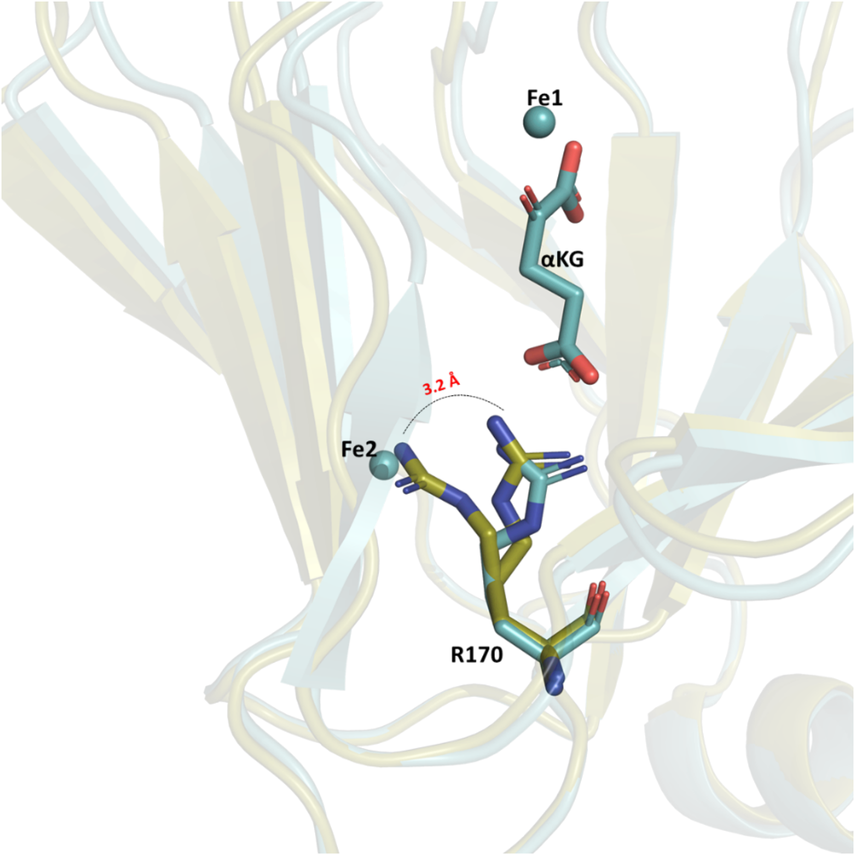

Supplementary Figure 18. Alternative conformation of Arg170. Overlay of trinary complex (light teal color) and apo-form (deep olive color) structures. R170 has alternative conformation in apo form in which Fe2 is absent. The distance is changed to 3.2 Å for two alternatives in apo form and mutant structures. Structural elucidation was done by PyMol (64-bit) software.

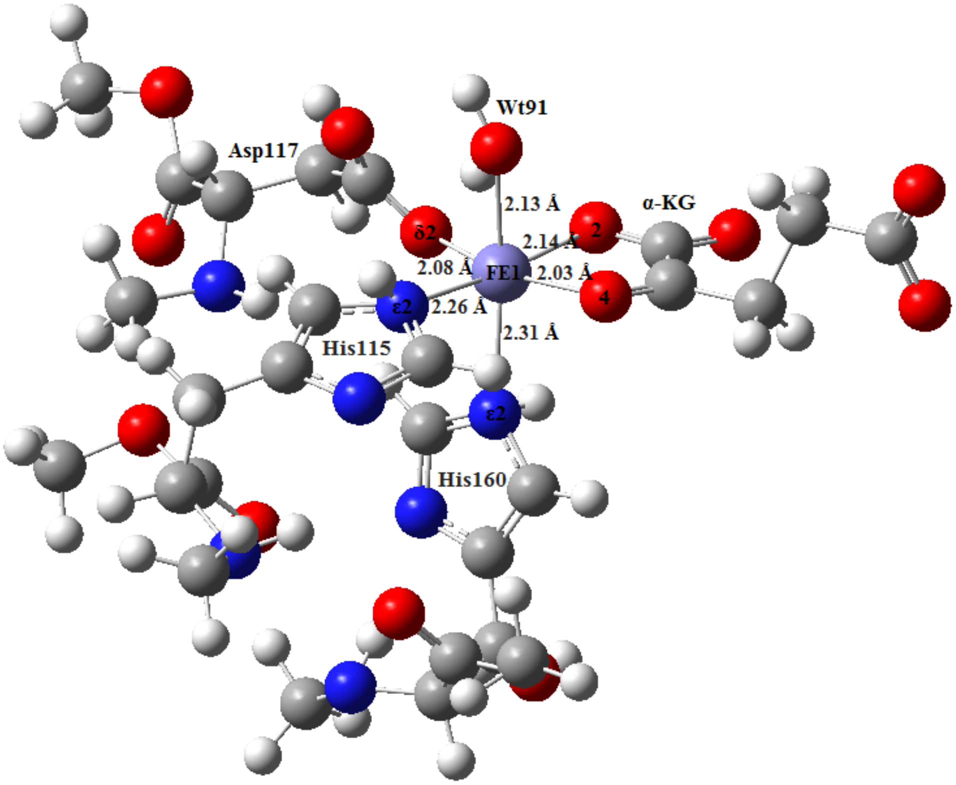

Supplementary Figure 19. NBO calculation for first iron (Fe1). The oxygen and nitrogen atoms of αKG, Wt91, Asp117, His115, and His160 donate their LP electrons to nearly empty LP* orbitals on the Fe (III) in both IS and HS states.

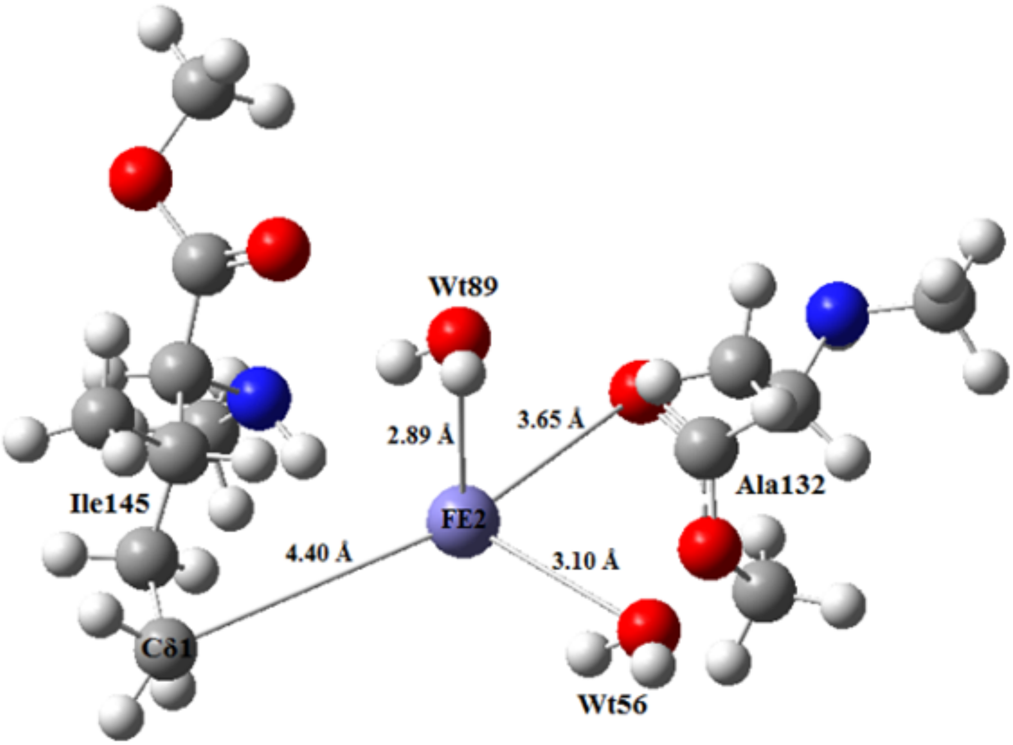

Supplementary Figure 20. NBO calculation for second iron (Fe2). The LP electrons of O and Cδ1 atoms of Ala132, Wt56, Wt89, and Ile145 are transferred into the LP* orbitals on the Fe (II) in both LS and IS states by four CT interactions.

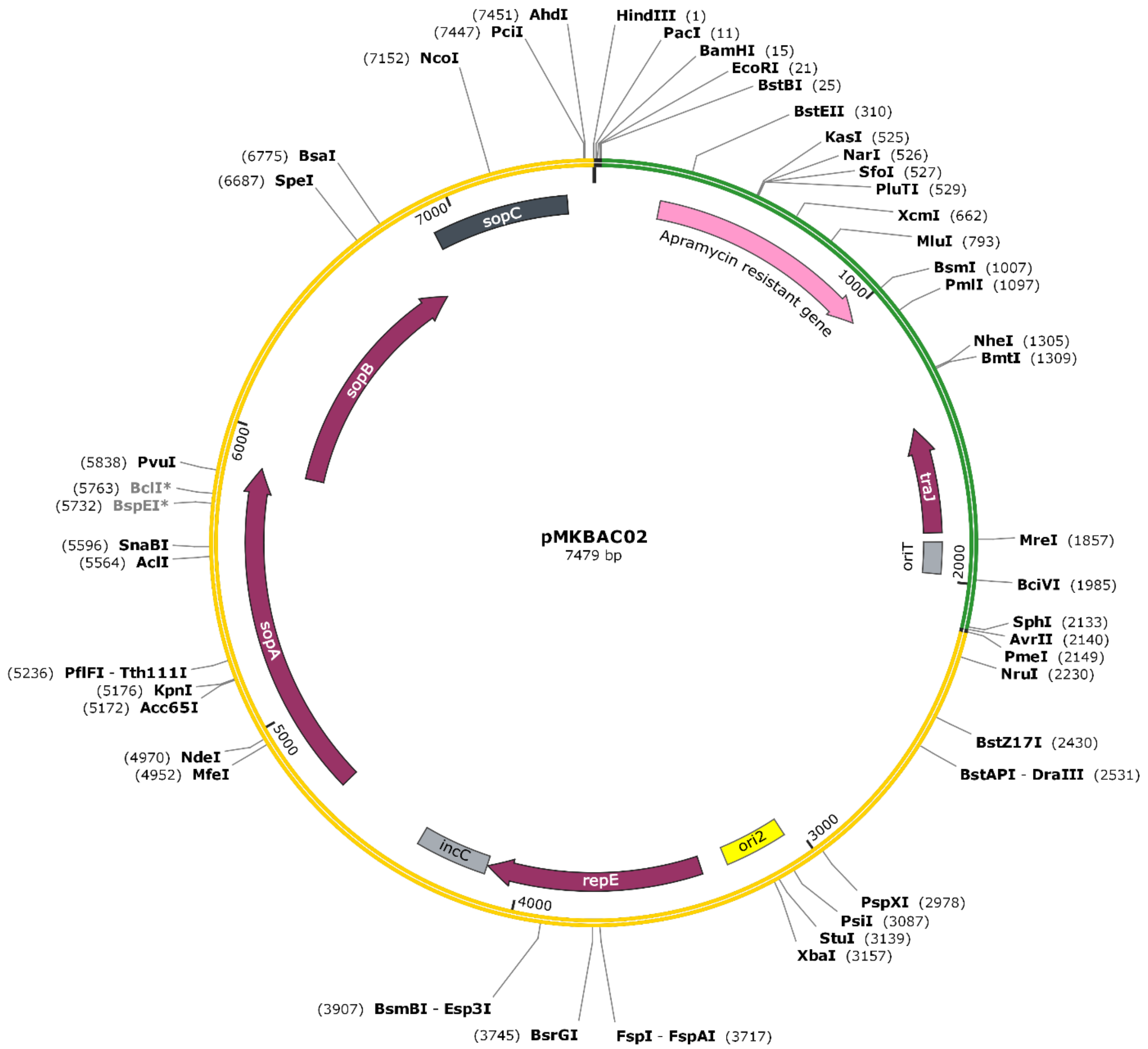

Supplementary Figure 21. The pMKBAC02-CpzH plasmid map. The origin of transfer region (*ori*T) and apramycin resistance gene, *aac*III(IV), were amplified from plasmid pGUSRolRPA3, and the backbone of the pMKBAC02 vector was amplified from plasmid pBeloBAC11.

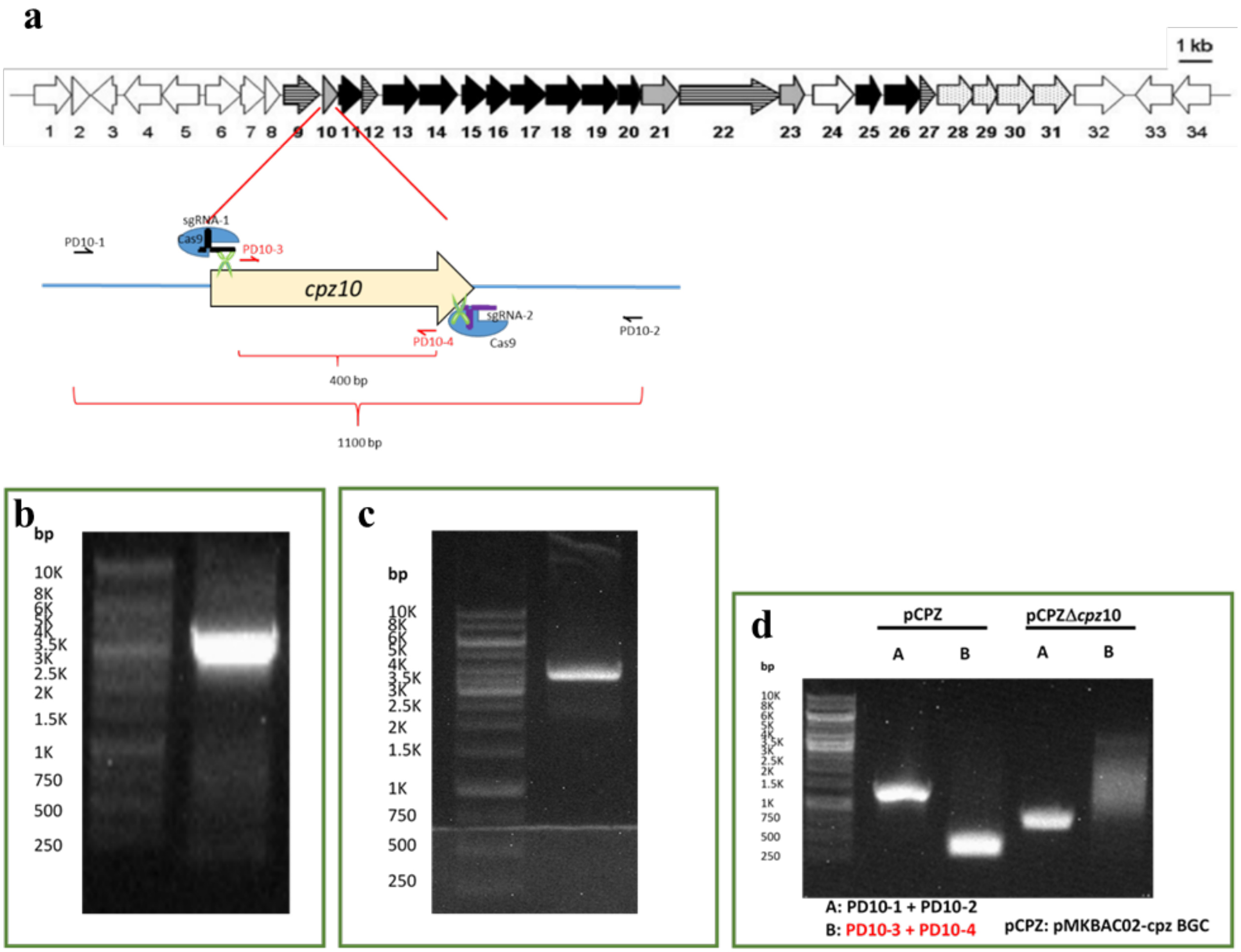

Supplementary Figure 22. In vitro CRISPR-Cas9 edition for deleting *cpz*10. a. The BAC system was constructed from *cpz*2 to *cpz*31. The figure explains how Cas9 sgRNA-1 and sgRNA-2 delete the target region. **b.** The qualified mutants were carefully chosen from apramycin containing MS agar plate, and verified by PCR within primer sets, p574-check-genome-BAC-cpz-F and p575-check-genome-BAC-cpz-R. **c.** The BAC plasmid carrying CPZ aglycone BGC was identified as pMKBAC02-CPZ. **d.** Gene-deficient mutants were selected on apramycin-adding medium agar plates, and verified by PCR using primers, 895-check-Dcpz10-F and 896-check-Dcpz10-R. The pCPZ and pCPZD*cpz*10 BGC were introduced into *Streptomyces albus* J1074 for heterologous expression of compounds individually.

a

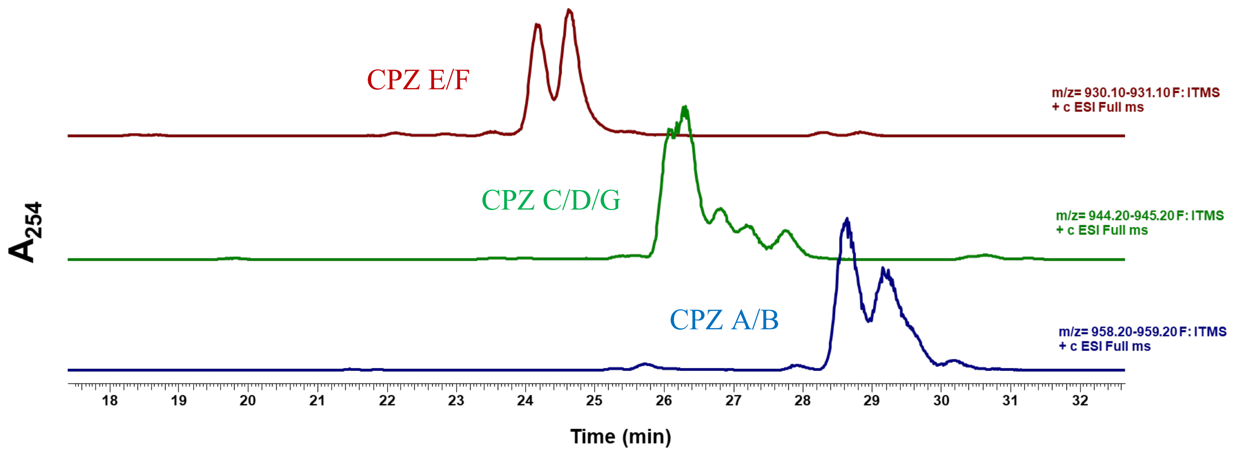
CPZ E/F
CPZ C/D/G
CPZ A/B
A254
m/z= 930.10-931.10 F: ITMS + c ESI Full ms
m/z= 944.20-945.20 F: ITMS + c ESI Full ms
m/z= 958.20-959.20 F: ITMS + c ESI Full ms
Time (min)

b

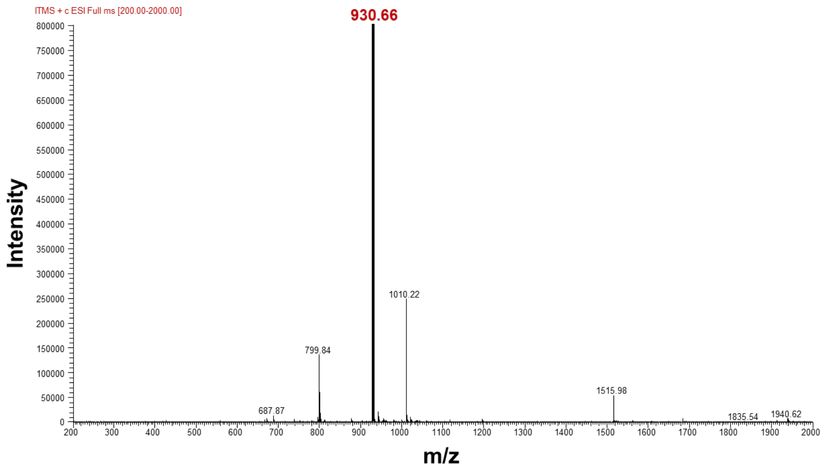
ITMS + c ESI Full ms [200.00-2000.00]
930.66
1010.22
799.84
1515.98
687.87
1835.54
1940.62
Intensity
m/z

c

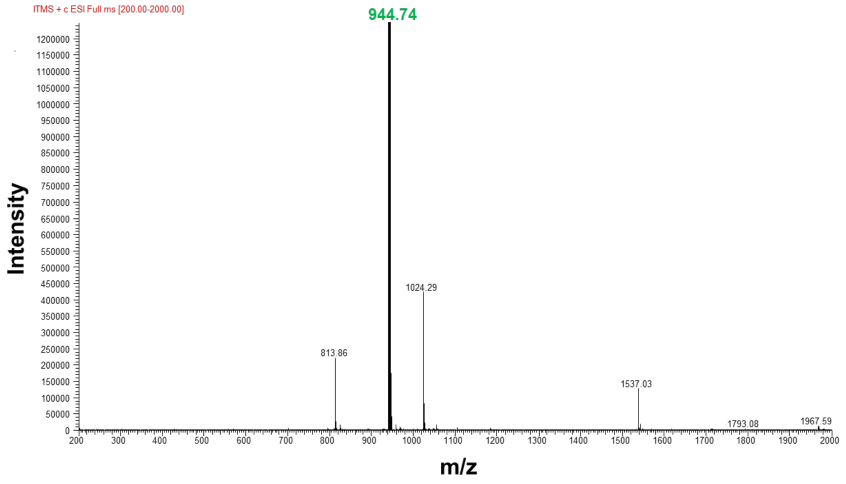
ITMS + c ESI Full ms [200.00-2000.00]
944.74
1024.29
813.86
1537.03
1793.08
1967.59
Intensity
m/z

d

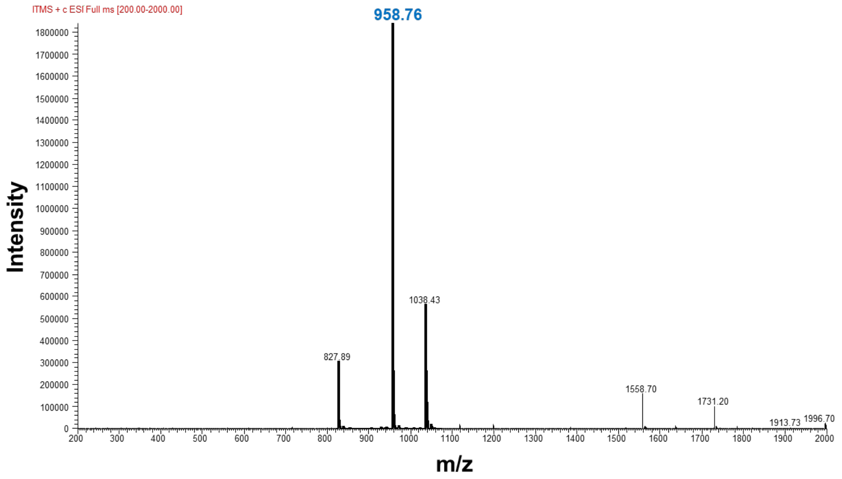

Supplementary Figure 23. Identification of caprazamycin derivatives produced by *cpz* BAC system strain. a. Selected ion monitoring chromatograms was obtained from LC-ESI-MS mass scans in positive mode. **b.** *Streptomyces albus* J1074::ErmE*-crpsc-*cpz*, m/z 930 (CPZ E/F aglycan). **c.** *Streptomyces albus* J1074::ErmE*-crpsc-*cpz*, 944 (CPZ C/D/G aglycan). and **d.** *Streptomyces* albus J1074::ErmE*-crpsc- *cpz*, 958 (CPZ A/B aglycone).

a

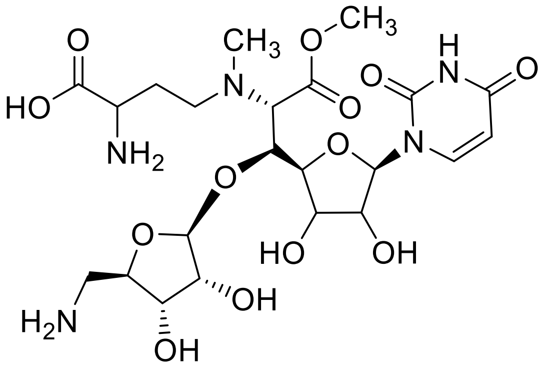

b

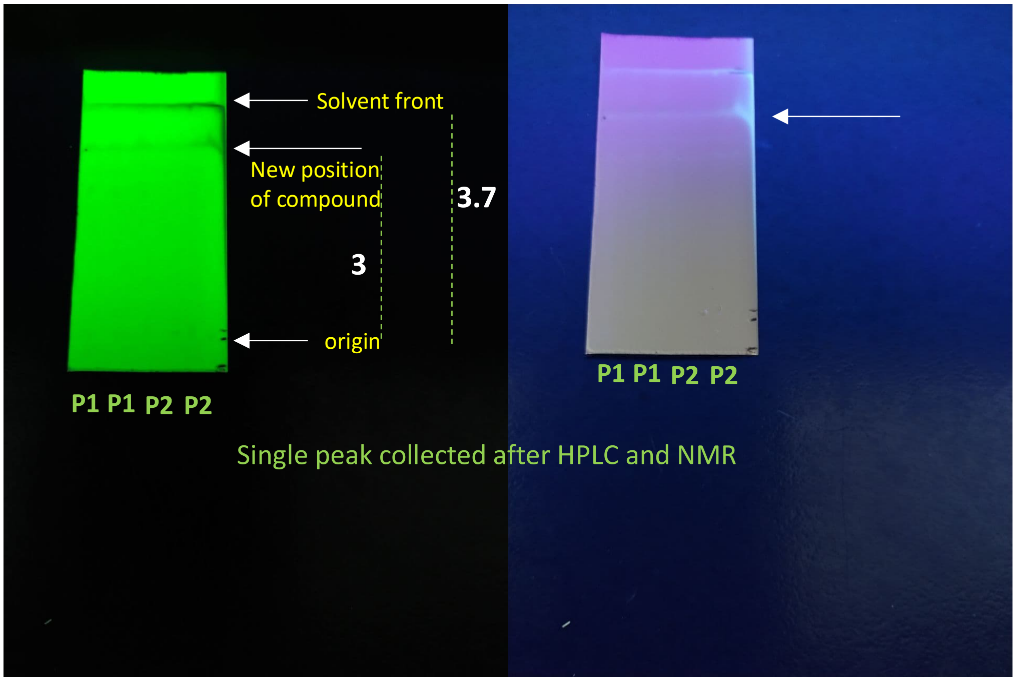

Supplementary Figure 24. The physical and chemical properties of compound 13. a. Chemical structure of compound **13.** This compound was collected from the *cpz*10 knock-out strain. The molecular formula is $C_{22}H_{35}N_5O_{13}$. **b.** Thin layer chromatograph for compound **13**: Stationary phase silica gel: (Kieselgel 60 F-254 made by Merck), developing solvent: n-butanol : ethanol : chloroform : 28% aqueous ammonia = 4:7:2:7 V/V. Calc. Rf value is 0.81. Appearance: creamy color powders. Molecular weight: SI-MS; m/z 578.23 (M^+ +1). Water soluble. UV: λ_{max} 262 nm (E_1 $cm^{1\%}$ 245) (ε 12,700), $\lambda_{0.1max}$ 0.1 N HCl 260 nm (E_1 $cm^{1\%}$ 240) (ε 12,450), λ_{max} 0.1 N NaOH 262 nm (E_1 $cm^{1\%}$ 190) (ε 9,850).

a

Protein (P)	Cpz27	LipX	LpmY	Mra6	PolQ2	Mur28
Cpz27		87/93	75/85	71/80	20/48	38/56
LipX			74/88	68/79	29/50	36/55
LpmY				74/86	17/46	38/56
Mra6					20/36	38/58
PolQ2						12/24
Mur28						

b

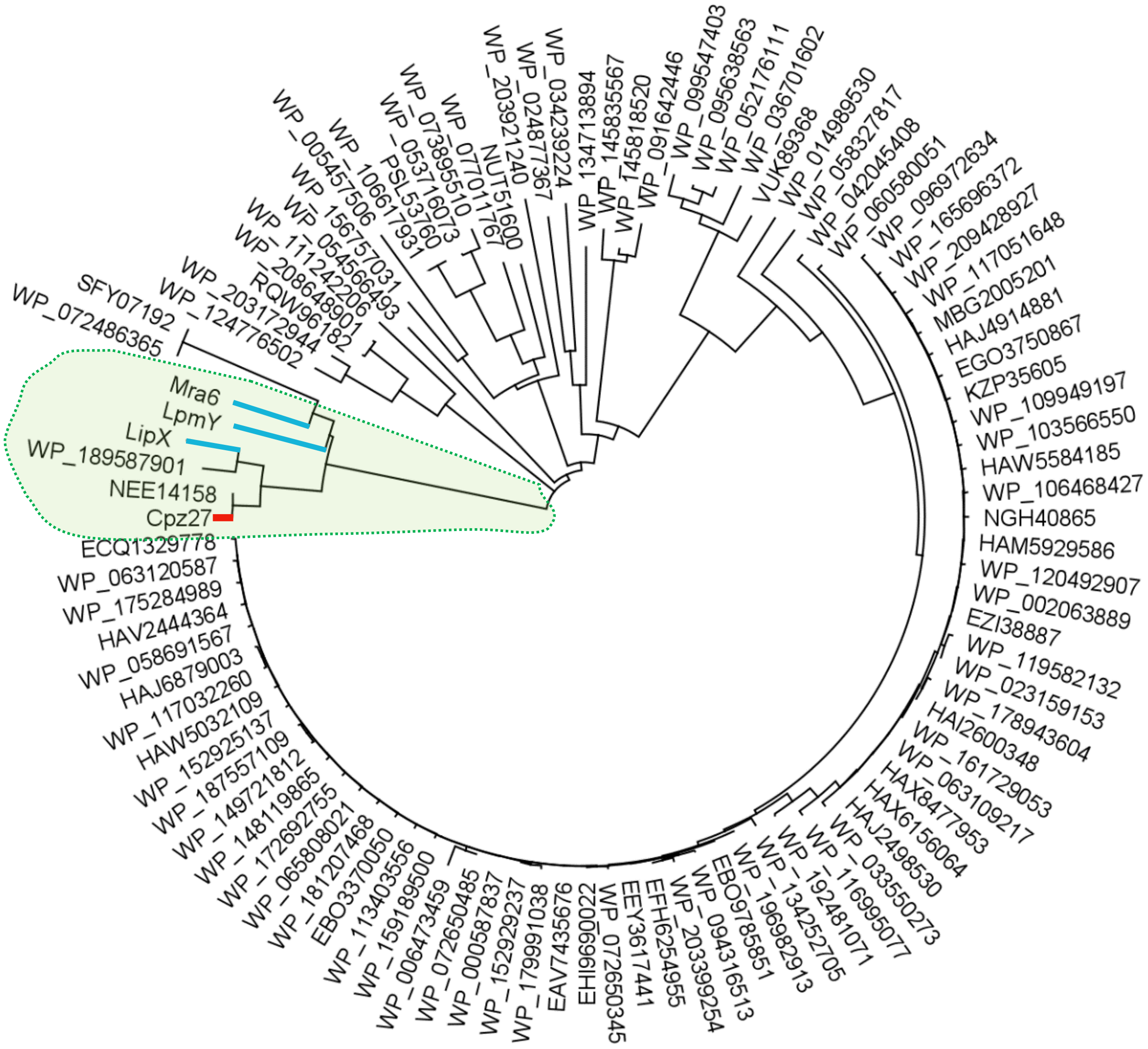

c

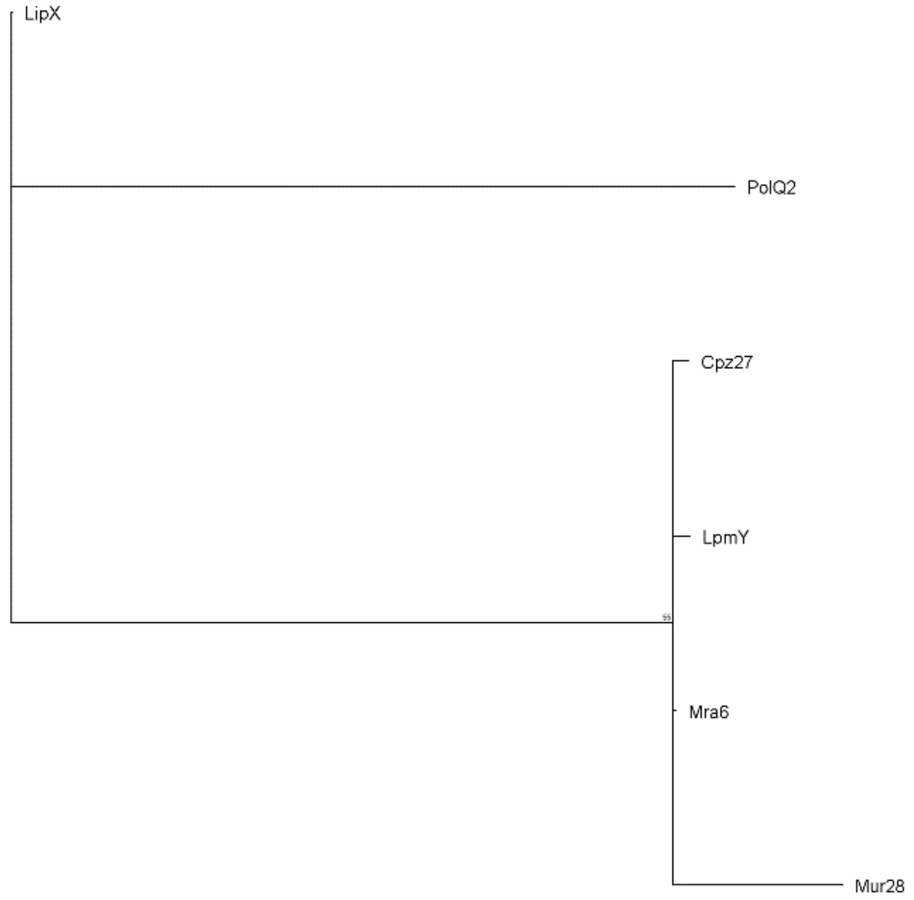

Supplementary Figure 25. Bioinformatic analysis of Cpz27. a. The sequence identity and similarity of Cpz27 and homologous proteins are showed in percentage. The Cpz27 has higher similarity and identity with LipX, LpmY and Mra6 respectively, and moreover, compared to PolQ2 and Mur28, Mur28 and PolQ2 are kinase proteins involved in the biosynthesis of Muraymycin and polyoxin. **b**. The phylogenetic analysis of Cpz27. Cpz27 was functionally assigned as a phosphotransferase in this research. The depicted tree and the table contain representative proteins from sequence alignments including: LipX, putative TmrB-like protein; LpmY, Kinase and Mra6, putative TmrB-like protein. **c,** Phylogenetic analysis of Cpz27 and selective nucleoside antibiotics homologous enzymes. Sequences were aligned with Geneious prime of Blosum45 and assembled by the Jukes-Cantor Genetic Distance model with the Neighbor-Joining Tree Build method. The phylogenetic tree was resampled using the Bootstrap method with 100 replicates.

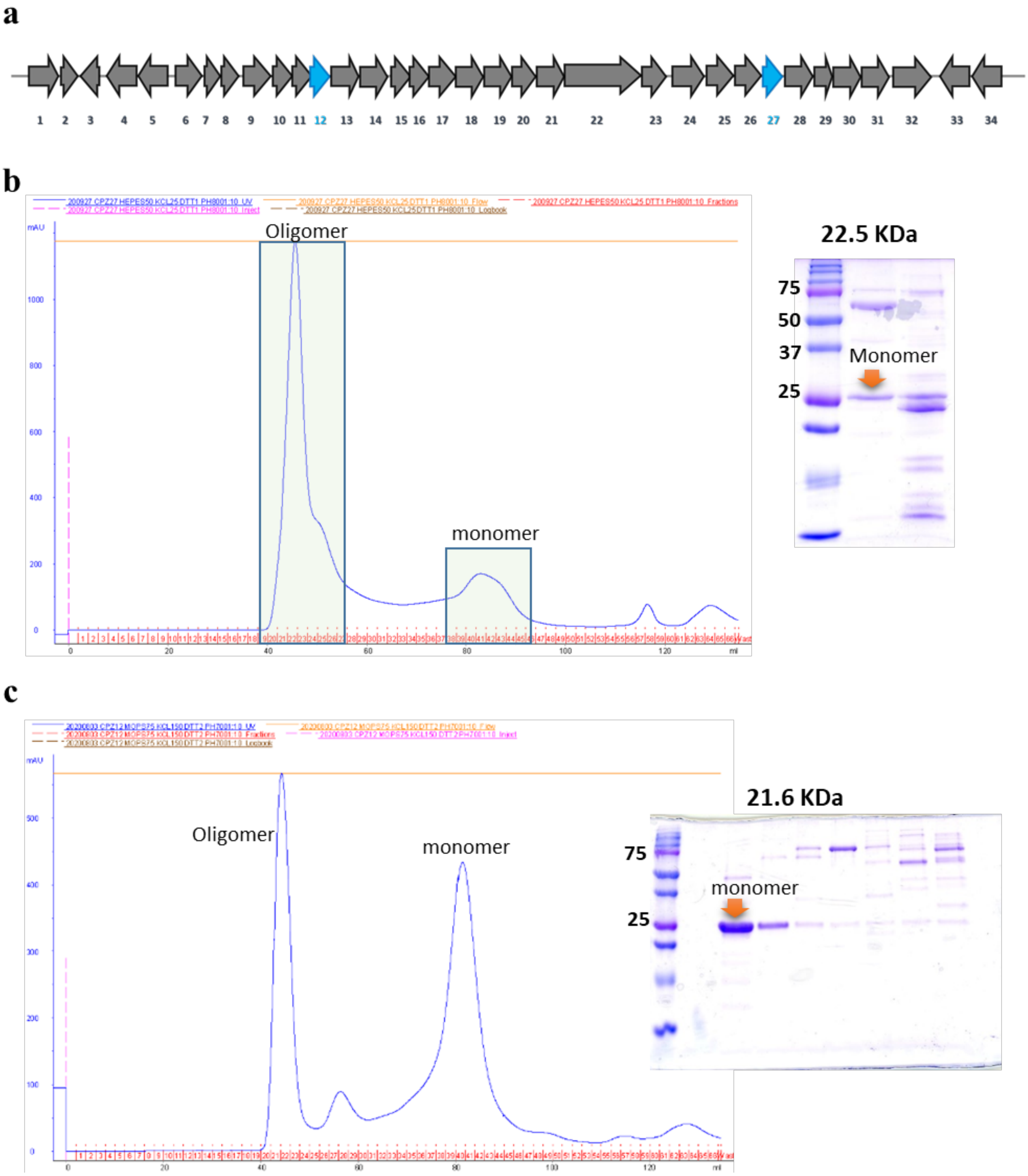

Supplementary Figure 26. SDS-PAGE, FPLC profile of Cpz27 and Cpz12. a, The location of the Cpz12 and Cpz27 in the *cpz* gene cluster. **b,** Fast Protein Liquid Chromatography (FPLC) profile for Cpz27 and SDS-PAGE of pure His$_6$-Cpz27. **c,** Fast Protein Liquid Chromatography (FPLC) profile for Cpz12.

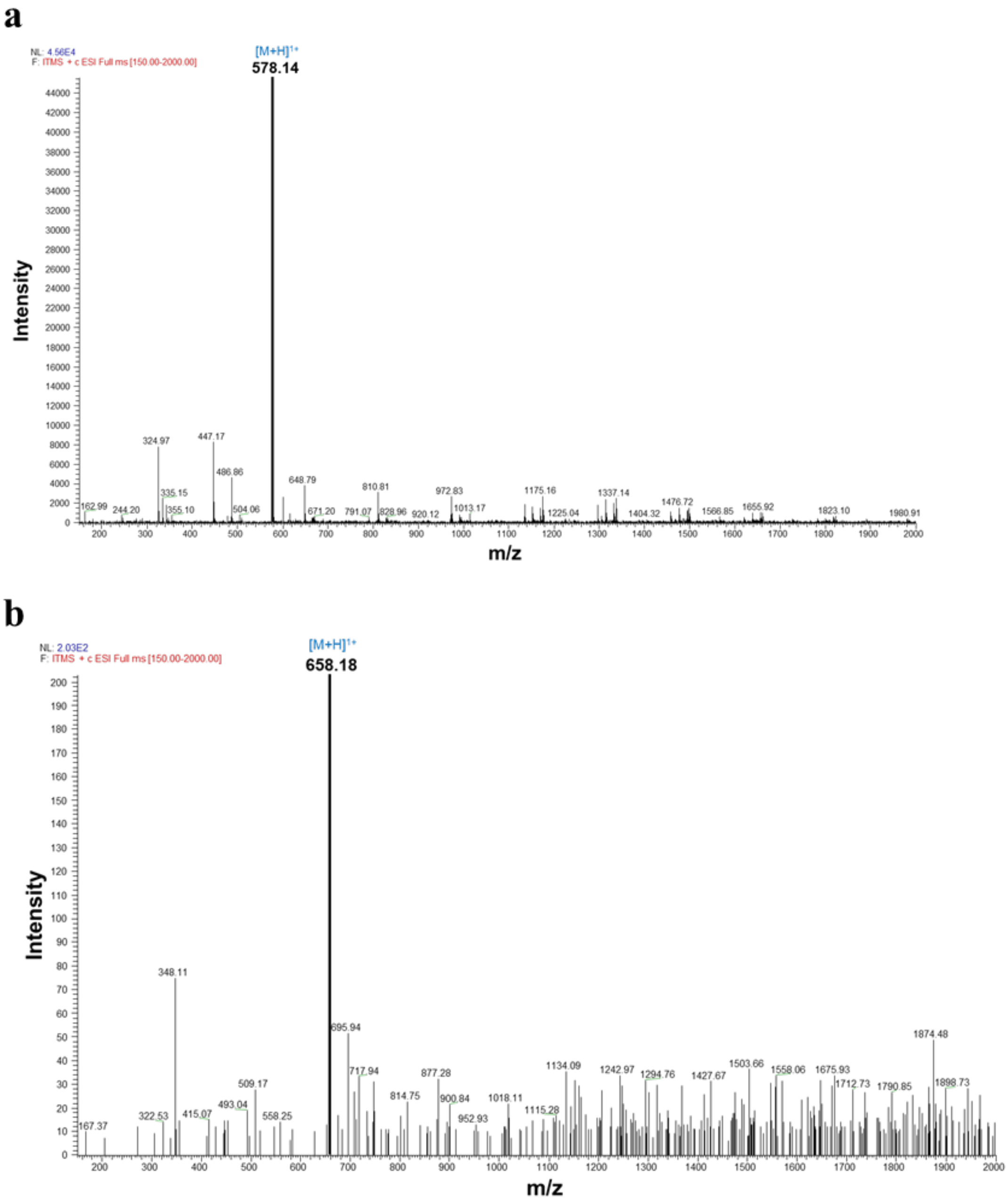

Supplementary Figure 27. Functional assignment of Cpz27. a, ESI-MS of **13**. Calc. for $C_{22}H_{35}N_5O_{13}$ expected $(M+H)^+$ ion at *m/z* = 578.23. **b,** ESI-MS of **14**. Calc. for $C_{22}H_{36}N_5O_{16}P$ expected $(M+H)^+$ ion at *m/z* = 658.19.

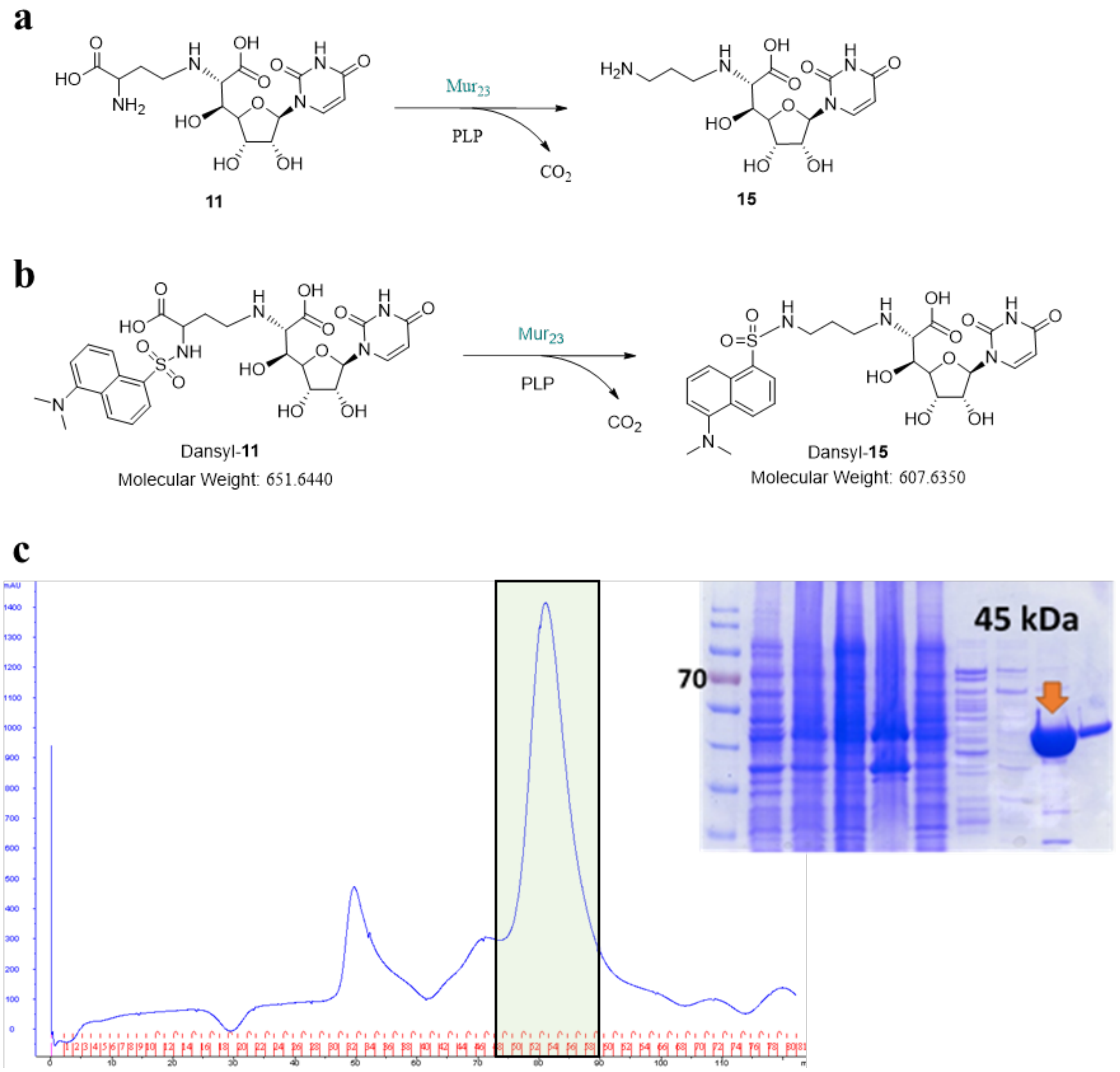

Supplementary Figure 28. a, Chemical reactions, SDS-PAGE and FPLC of Mur23. The chemical reaction catalyzed by Mur23 can produce compound **15**. **b,** Chemical reaction catalyzed by Mur23 was probed by dansyl chloride to change the retention time in HPLC profile. **c,** Fast Protein Liquid Chromatography (FPLC) profile for Mur23 and SDS-PAGE of purified His_6-Mur23.

a

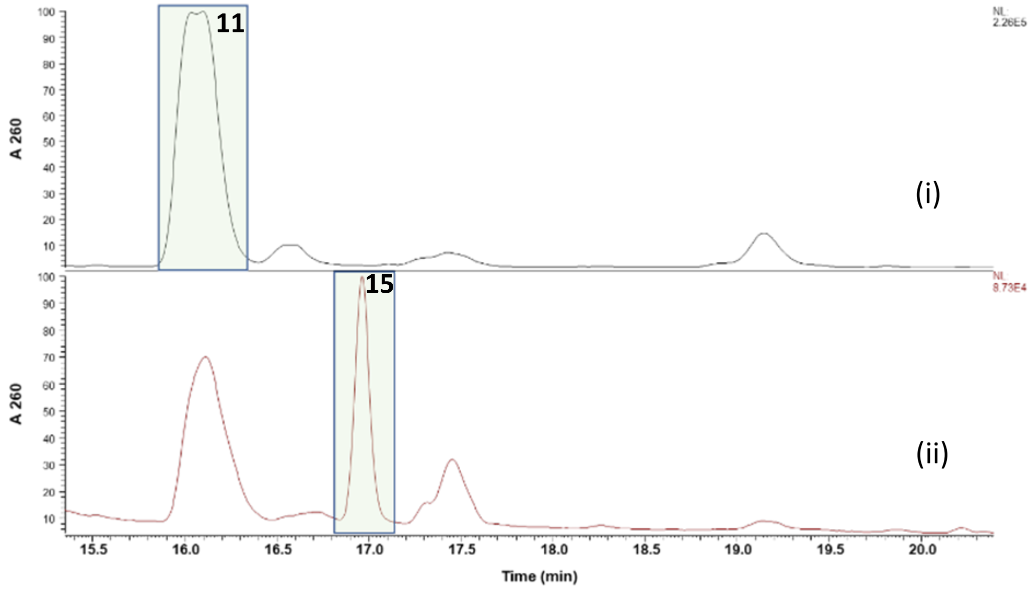

11
15
NL: 2.26E5
NL: 8.73E4
A 260
(i)
(ii)
Time (min)

b

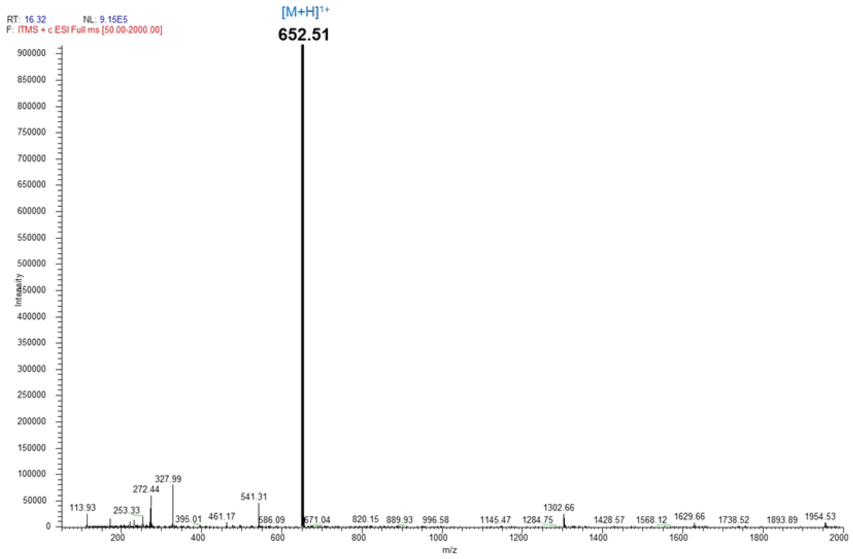

RT: 16.32
NL: 9.15E5
F: ITMS + c ESI Full ms [50.00-2000.00]
[M+H]1+
652.51
Intensity
m/z

c

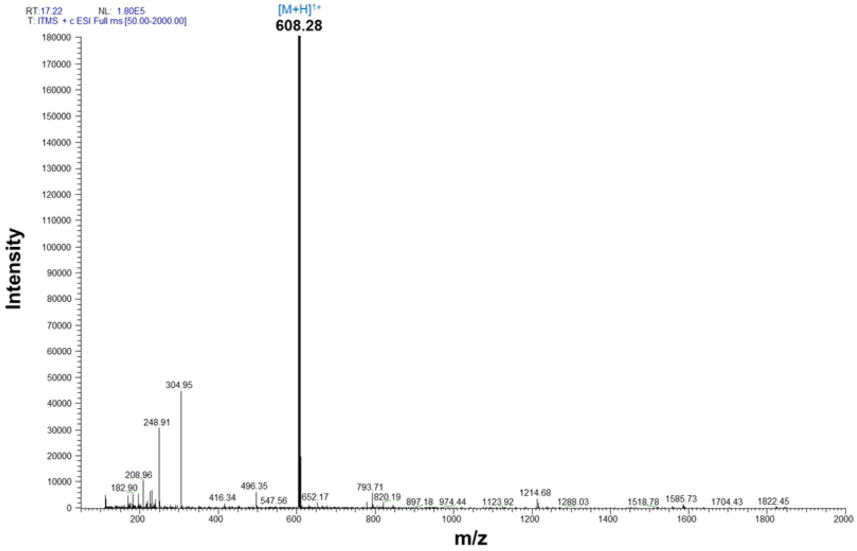

Supplementary Figure 29. Functional assignment of Mur23. a, Chemical reaction catalyzed by Mur23, (i) control, compound **11** after adding dansyl chloride to derivatize the substrate without enzyme and (ii) the reaction catalyzed by Mur23 starting from **11** and PLP after adding dansyl chloride. **b,** (+)-ESI-MS for **11** ($C_{15}H_{22}N_4O_{10}$) has an expected $(M+H)^+$ ion at *m/z* = 419.14; the derivatized substrate ($C_{27}H_{33}N_5O_{12}S$) has an expected $(M+H)^+$ ion at *m/z* = 652.19 and detected ion at *m/z* = 652.51. **c,** (+)-ESI-MS for **15** ($C_{14}H_{22}N_4O_8$) has an expected $(M+H)^+$ ion at *m/z* = 375.15; the derivatized product ($C_{26}H_{33}N_5O_{10}S$) has an expected $(M+H)^+$ ion at *m/z* = 608.20 and detected ion at *m/z* = 608.28.

a

Protein	Cpz15	Cpz10	LipG	LpmH	LipL	Mra18	LpmM	Jaw7	SphK	Mur16	Cpr19	CapA	Orf7
Cpz15		18/23	16/26	26/29	86/90	20/23	82/89	79/86	19/22	44/56	38/54	36/51	36/51
Cpz10			91/94	87/93	18/25	84/91	16/20	21/30	46/65	17/25	23/30	23/30	17/25
LipG				83/92	16/28	80/90	14/23	19/34	45/65	15/26	19/30	21/31	18/28
LpmH					29/32	83/91	23/26	27/36	46/65	23/40	20/32	26/36	20/36
LipL						20/26	80/86	77/85	22/25	45/56	39/52	36/50	37/50
Mra18							17/20	21/33	46/66	16/30	20/26	20/30	16/26
LpmM								80/85	19/22	43/56	39/53	37/51	39/52
Jaw7									16/23	44/54	37/52	37/51	36/49
SphK										22/29	16/22	18/29	18/25
Mur16											53/67	52/65	52/66
Cpr19												76/88	75/88
CapA													84/91
Orf7													

b

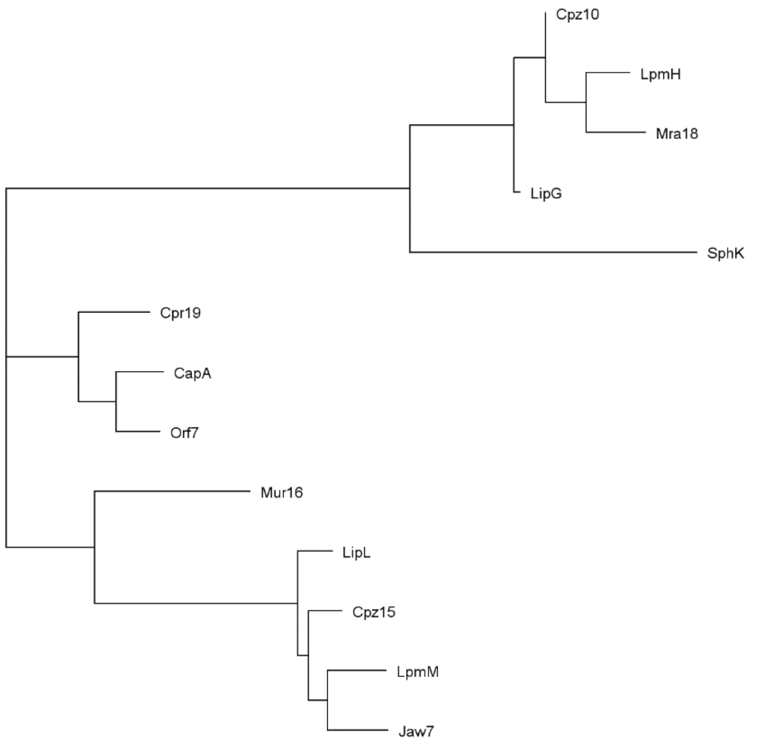

Supplementary Figure 30. Bioinformatic analysis of Cpz10 and Cpz15. a, The sequence identity and similarity of Cpz10 and Cpz15 in percentage. The table shows that Cpz10 and Cpz15 do not have higher similarity and identity to each other. **b**, Phylogenetic analysis of Cpz10, Cpz15 and other selective nucleoside antibiotics homologous enzymes together with the Hyps enzyme (PDB code. 4P7X.) The results show that they are structurally close to Cpz10 protein structure. Sequences were aligned with Geneious prime of Blosum45 and assembled by the Jukes-Cantor Genetic Distance model with the Neighbor-Joining Tree Build method. The phylogenetic tree was resampled using the Bootstrap method with 100 replicates.

Supplementary note 1

Spectroscopic assignments for methionine sulfoxide in D_2O

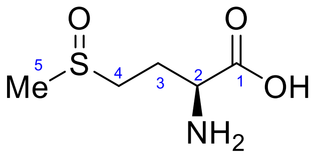

Position No.	δ_H (mult, J in Hz)[a]	δ_C (type)[b]
1		171.1 (C)
2	4.09 (dd, 6.4, 6.2)	51.8 (CH)
3	2.26 (m)	23.3 (CH_2)
	2.32 (m)	
4	2.88 (ddd, 13.6, 10.3, 5.4)	47.9 (CH_2)
	3.07 (ddd, 13.6, 10.5, 5.8)	
5	2.64 (s)	36.4 (CH_3)

[a] The δ_H values were measured in 600 MHz NMR.

[b] The δ_C values were measured in 150 MHz NMR.

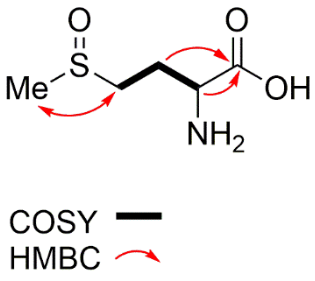

COSY and HMBC corrlections of methionine sulfoxide. The COSY correlations were presented in black lines while the key HMBC correlations were showen red arrows.

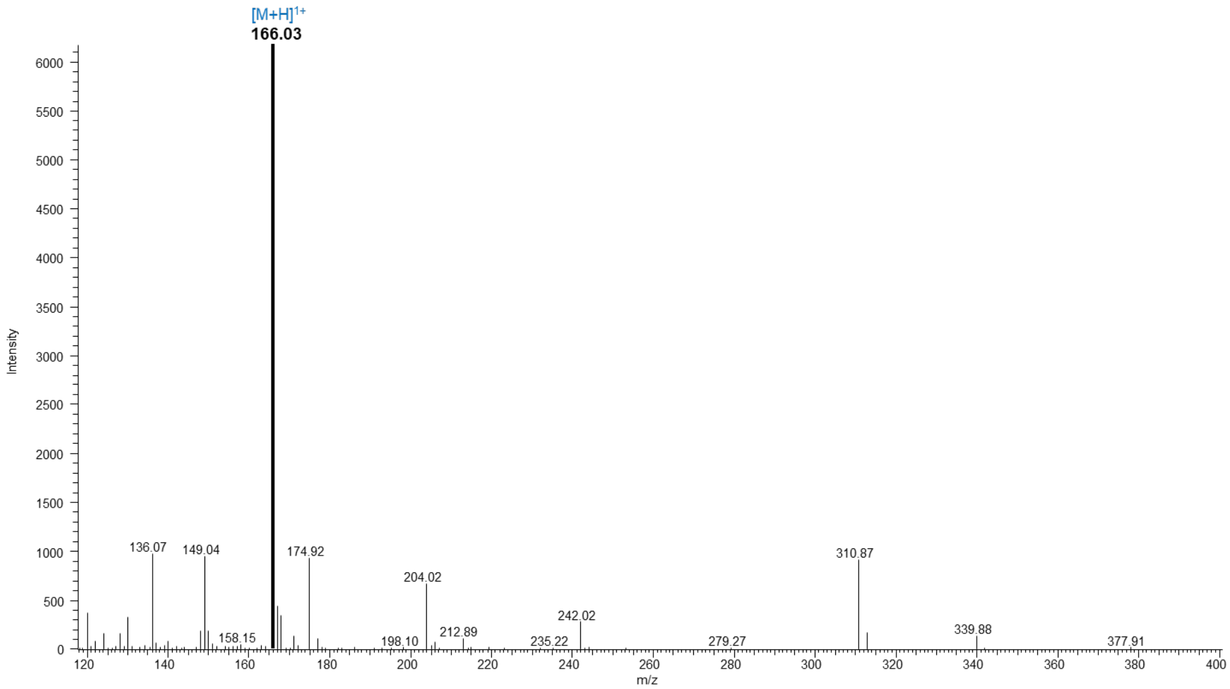

(+)-ESI-MS of MSO. Calc. for $C_5H_{11}NO_3S$, expected $(M+H)^+$ ion at m/z = 165.05, detected: 165.03.

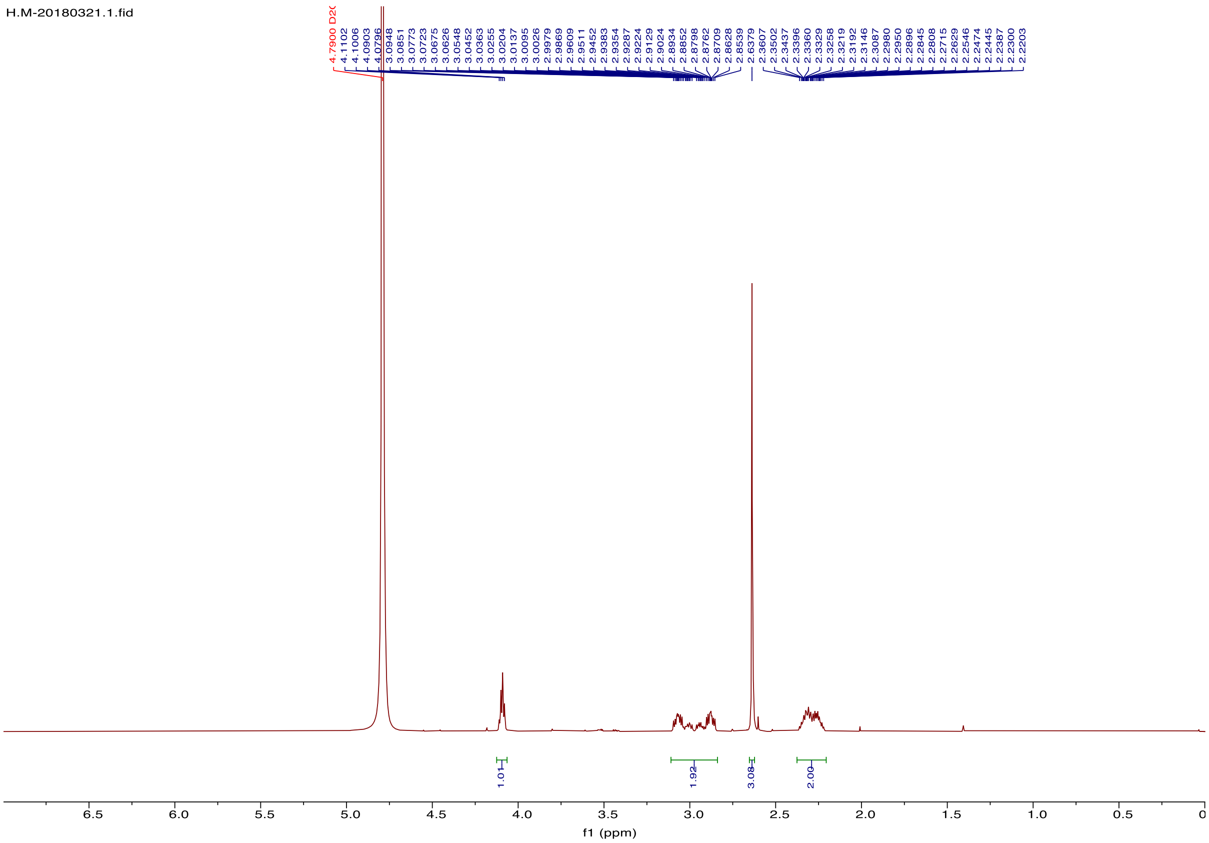

^1H NMR of methionine sulfoxide in D_2O (600 MHz).

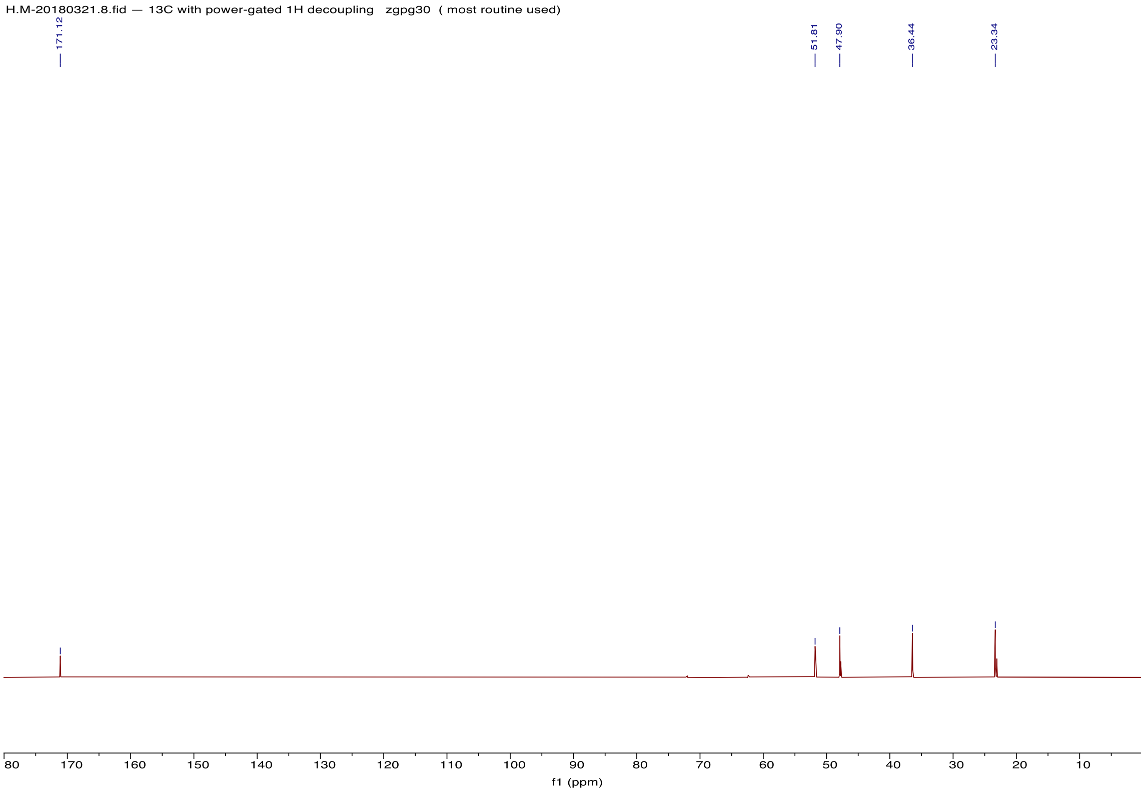

^{13}C NMR of methionine sulfoxide in D_2O (150 MHz).

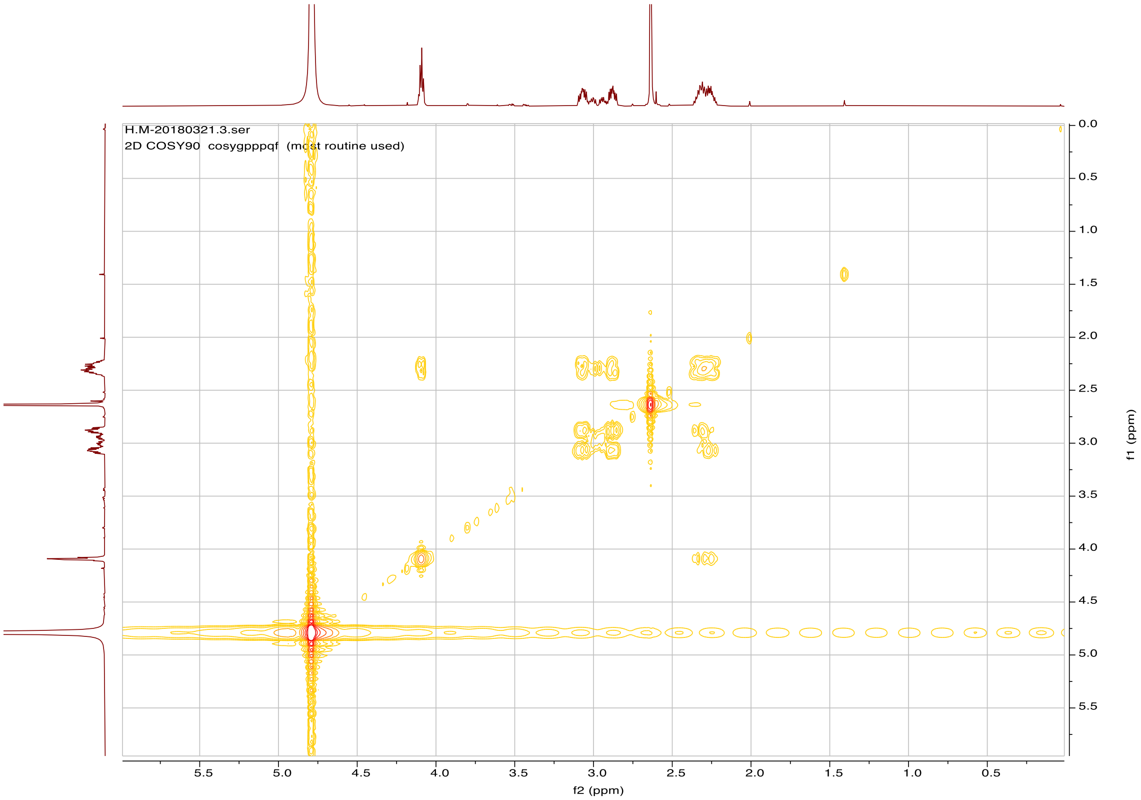

^1H–^1H COSY spectrum of methionine sulfoxide in D_2O.

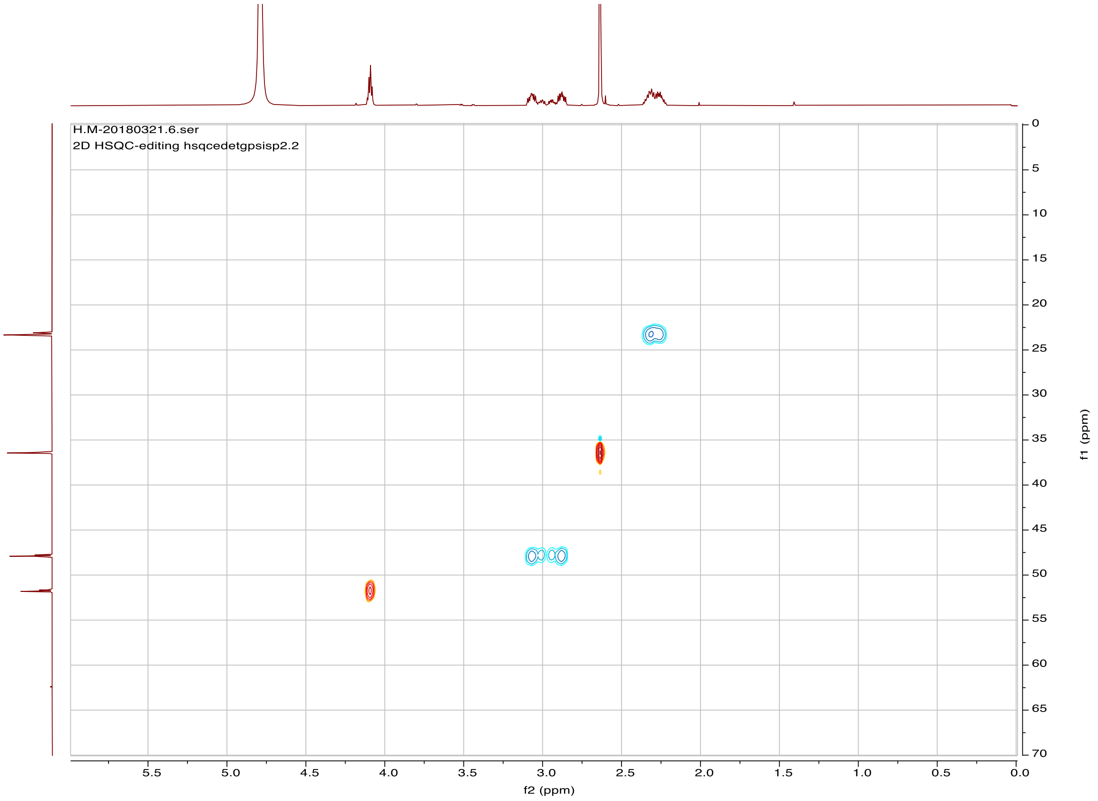

HSQC spectrum of methionine sulfoxide in D_2O.

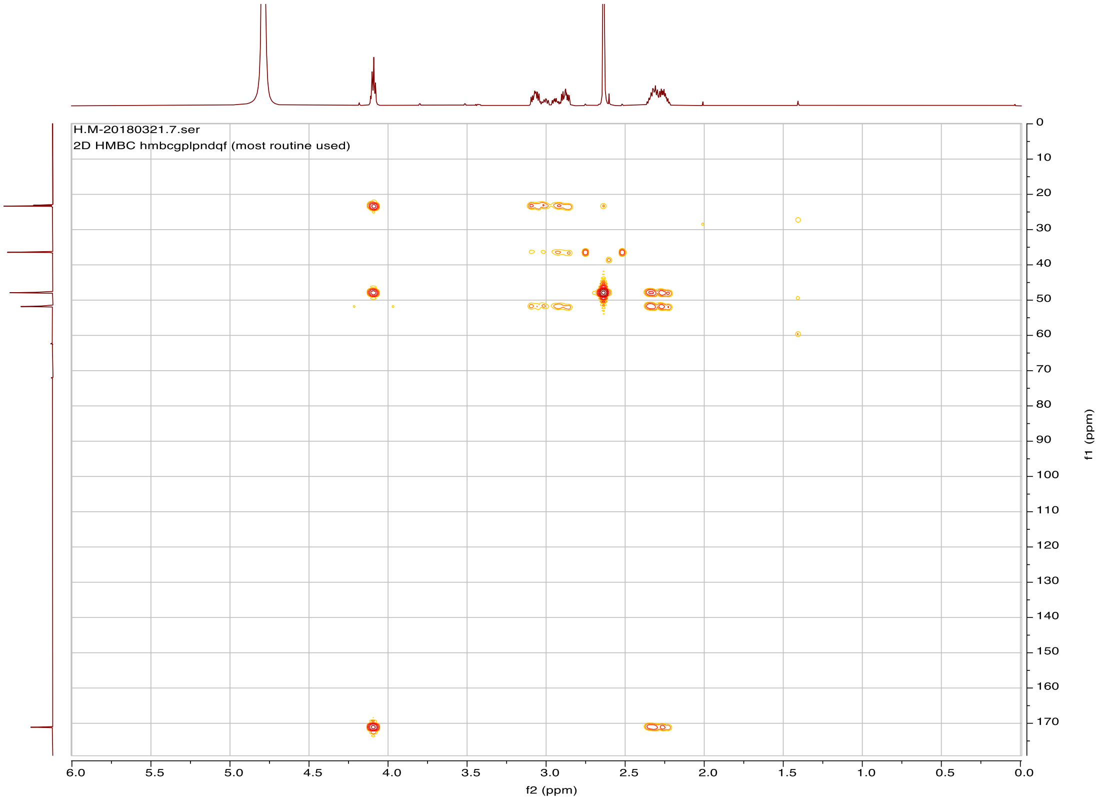

HMBC spectrum of methionine sulfoxide D_2O.

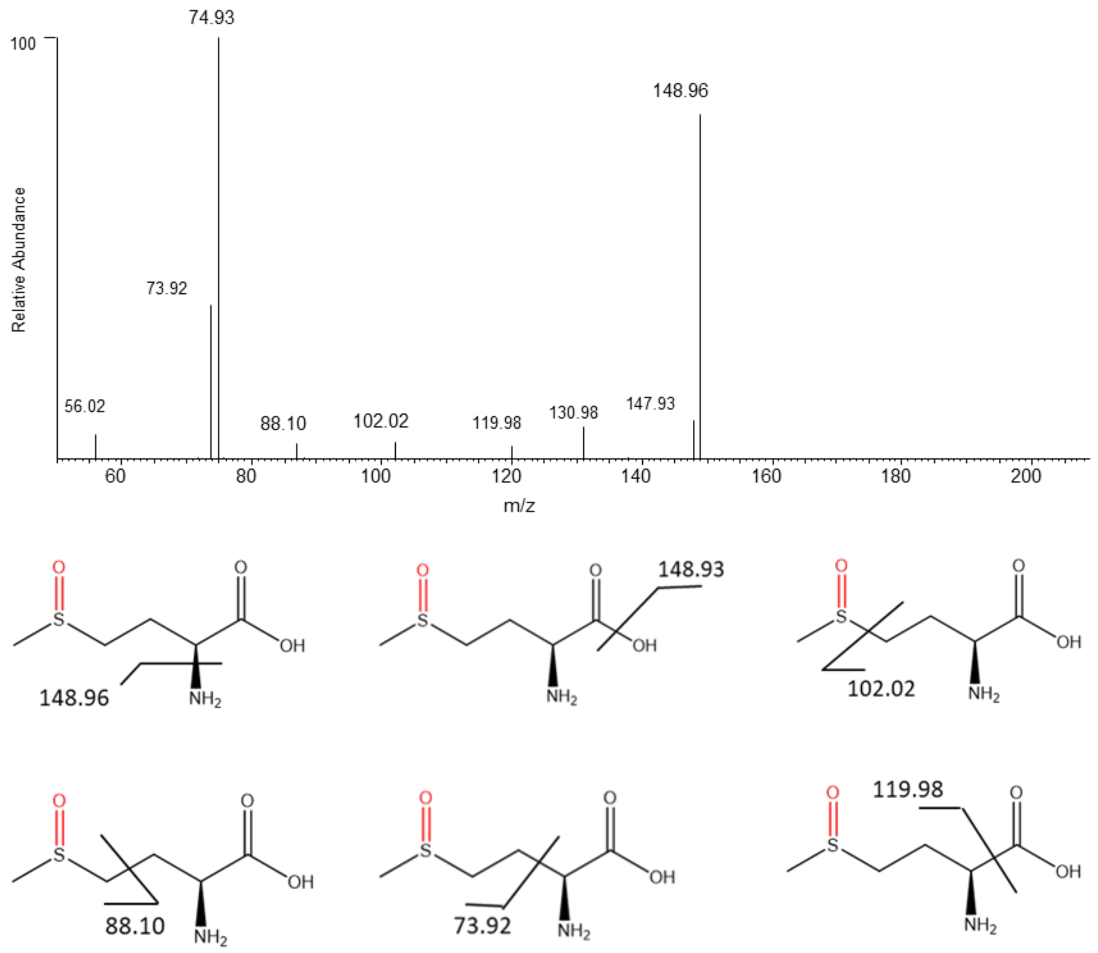

MS/MS of methionine sulfoxide.

Spectroscopic assignments for compound 13 in D_2O

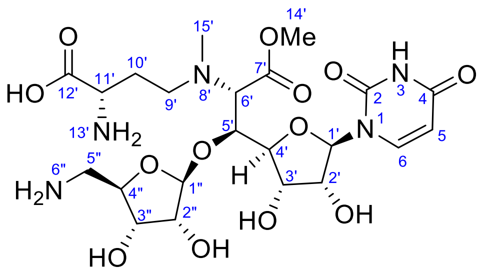

Position No.	δ_H (mult, J in Hz)[a]	δ_C (type)[b]
2		151.5 (C)
4		166.3 (CH)
5	5.89 (d, 8.0)	102.2 (C)
6	7.77 (d, 8.0)	141.9 (C)
1′	5.79 (d, 3.3)	89.3 (CH)
2′	4.37 (m)	73.6 (CH)
3′	4.31 (m)	69.4 (CH)
4′	4.30 (m)	82.2 (CH)
5′	4.53 (dd, 3.3, 6.5)	77.2 (CH)
6′	3.86 (dd, overlapping)	68.2 (CH)
7′		170.4 (C)
9′	2.87 (m)	51.8 (CH_2)
	2.99 (m)	
10′	2.00 (m)	26.8 (CH_2)
	2.21 (m)	
11′	3.86 (m)	53.7 (CH_2)
12′		174.3 (C)
14′ (*O*-Me)	3.82 (s)	52.4 (CH_3)
15′ (*N*-Me)	2.47 (s)	37.4 (CH_3)
1″	5.17 (br s)	109.8 (CH)
2″	4.09 (dd, overlapping)	74.8 (CH)
3″	4.08 (dd, overlapping)	72.0 (CH)
4″	4.10 (dd, overlapping)	78.6 (CH)
5″	3.06 (dd, 8.5, 13.4)	42.6 (CH_2)
	3.35 (dd, 3.4, 13.4)	

[a] The δ_H values were measured in 600 MHz NMR.
[b] The δ_C values were measured in 150 MHz NMR.

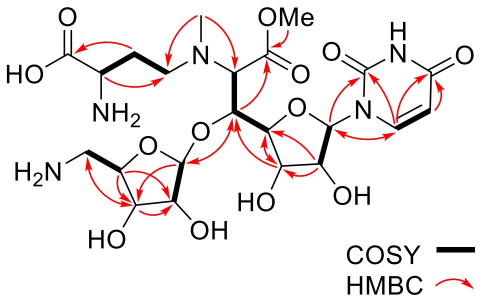

COSY and HMBC corrlections of the isolated intermediate (**13**) from Cpz10 KO. The COSY correlations were presented in black bold lines while the key HMBC correlations were showen red arrows.

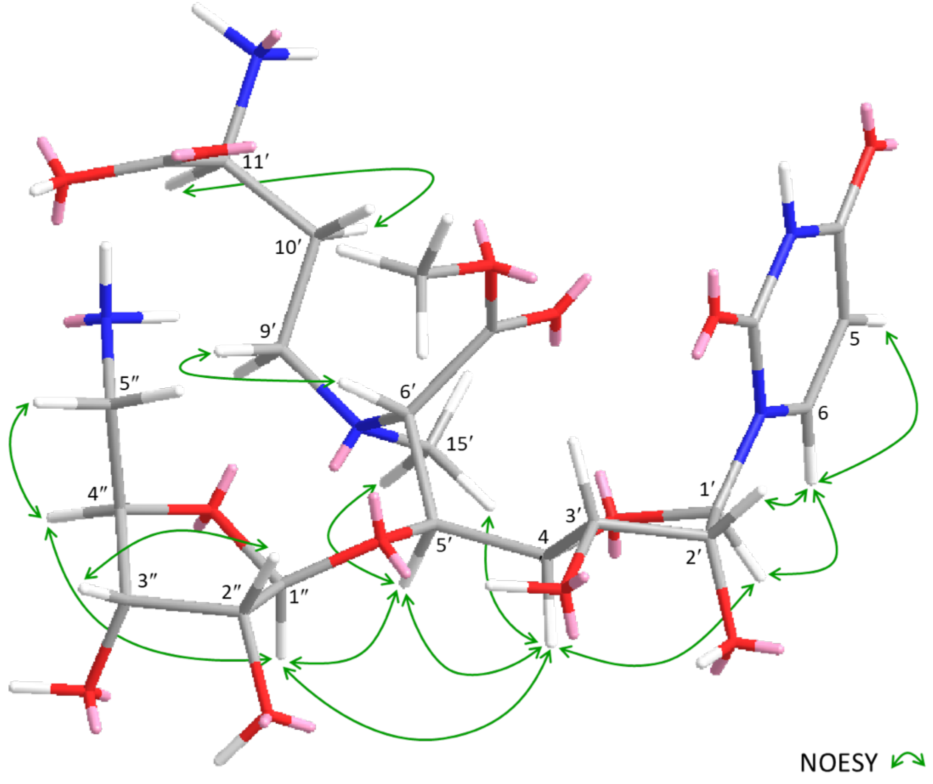

NOESY correlations of **13** (shown in green dihead arrows).

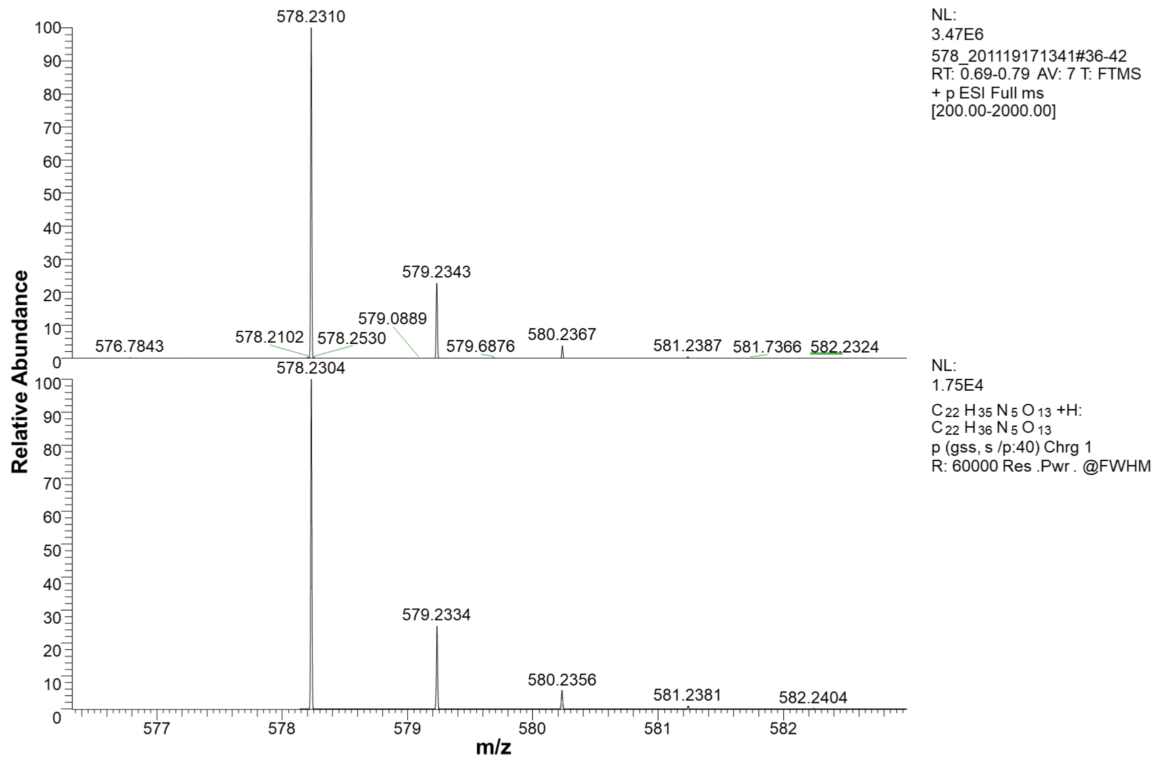

(+)-HR-ESI-MS of **13** calc. for $C_{22}H_{35}N_5O_{13}$, expected $(M+H)^+$ ion at m/z = 578.2304, detected: 578.2310.

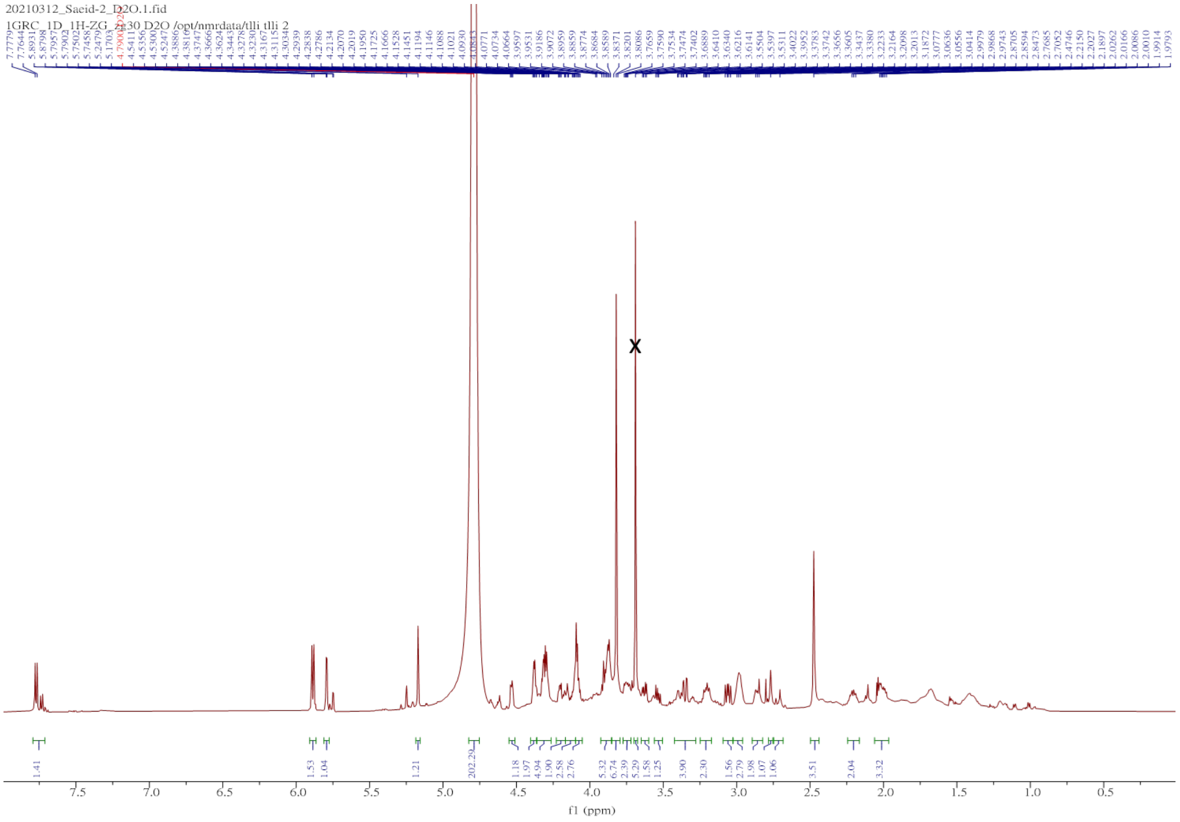

^{1}H NMR of compound **13** in D_2O (600 MHz)

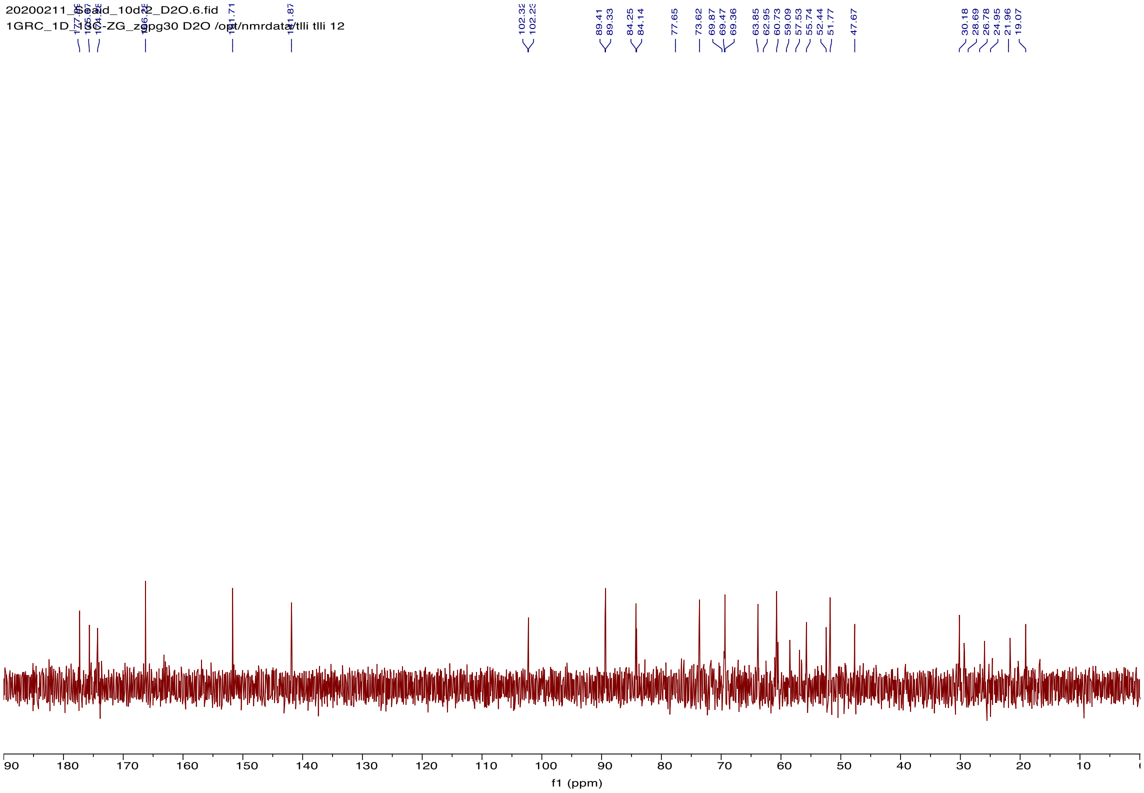

^{13}C NMR of compound **13** in D_2O (150 MHz)

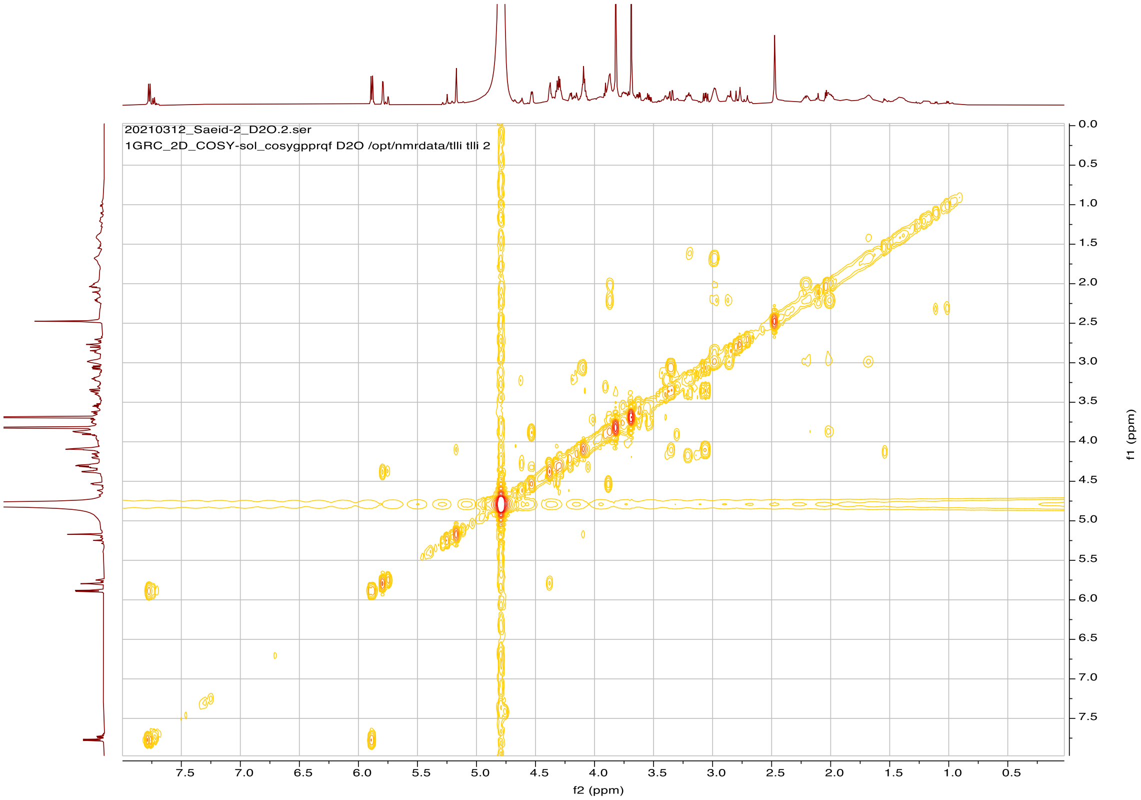

^1H–^1H COSY spectrum of compound **13** in D_2O

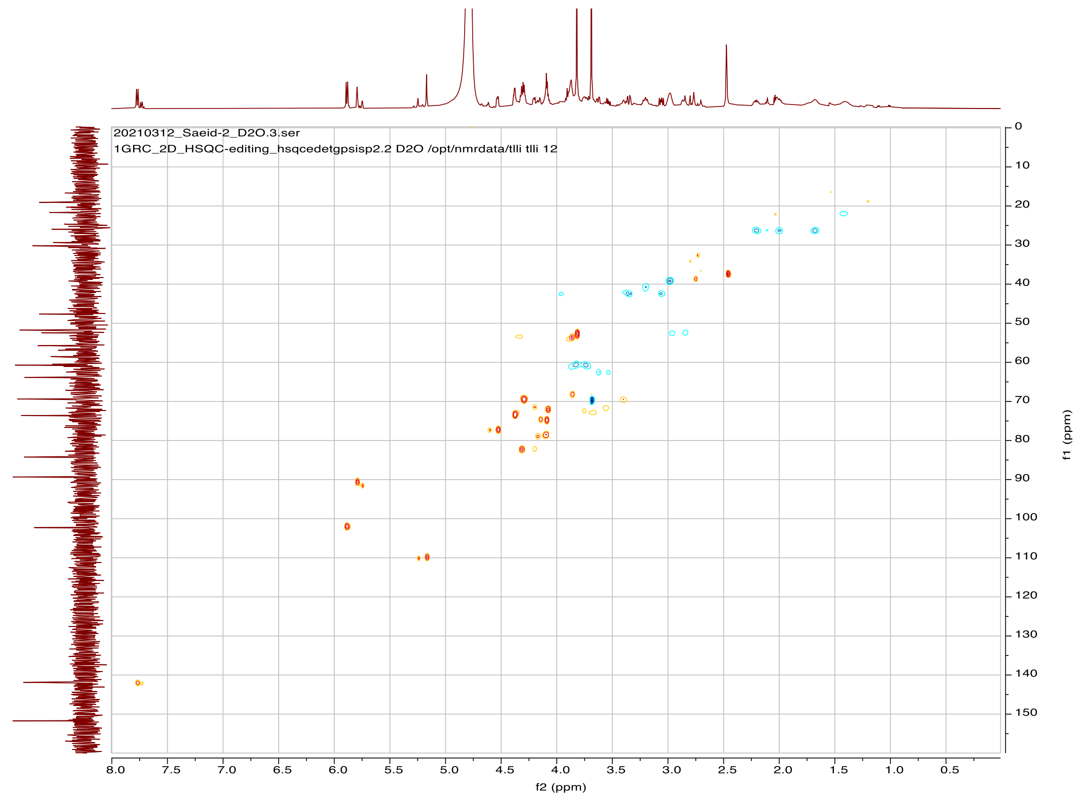

HSQC spectrum of compound **13** in D_2O

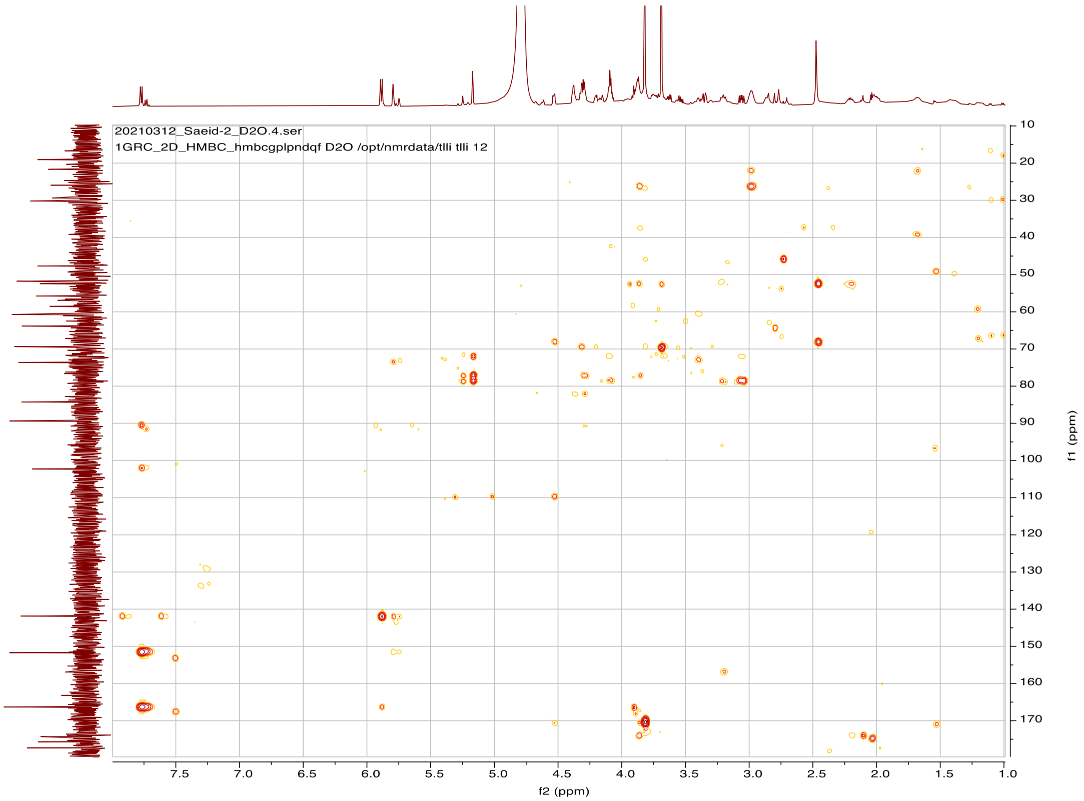

HMBC spectrum of compound **13** in D_2O

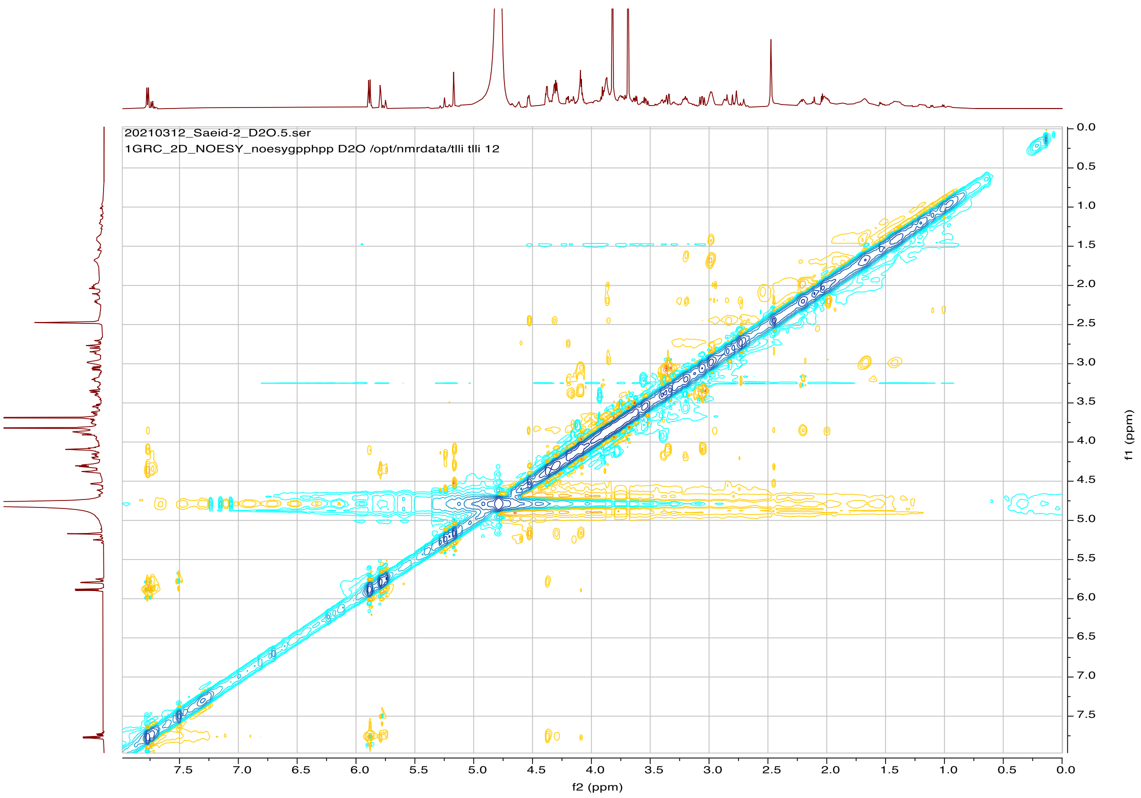

NOESY spectrum of compound **13** in D_2O

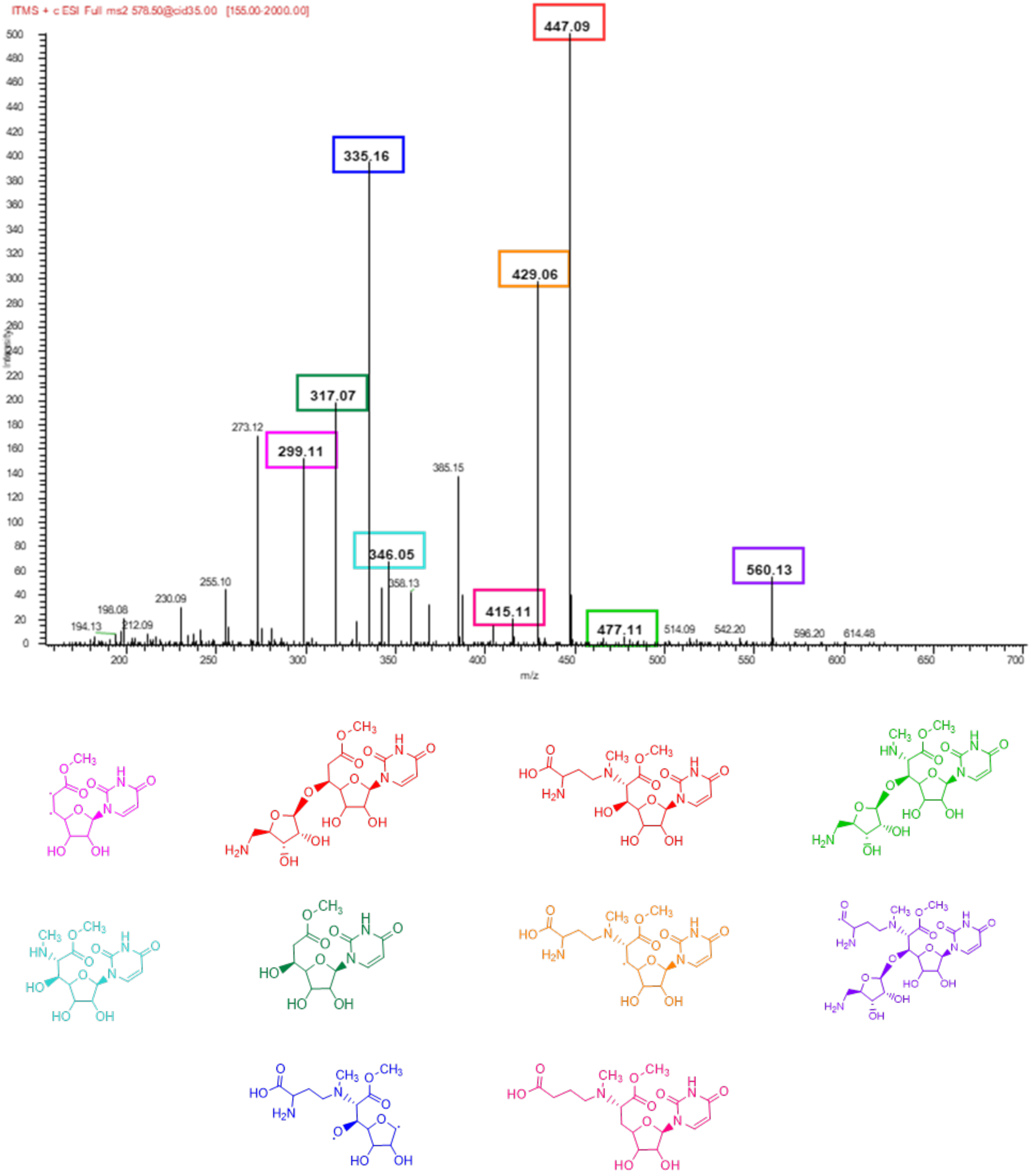

MS/MS of compound **13**

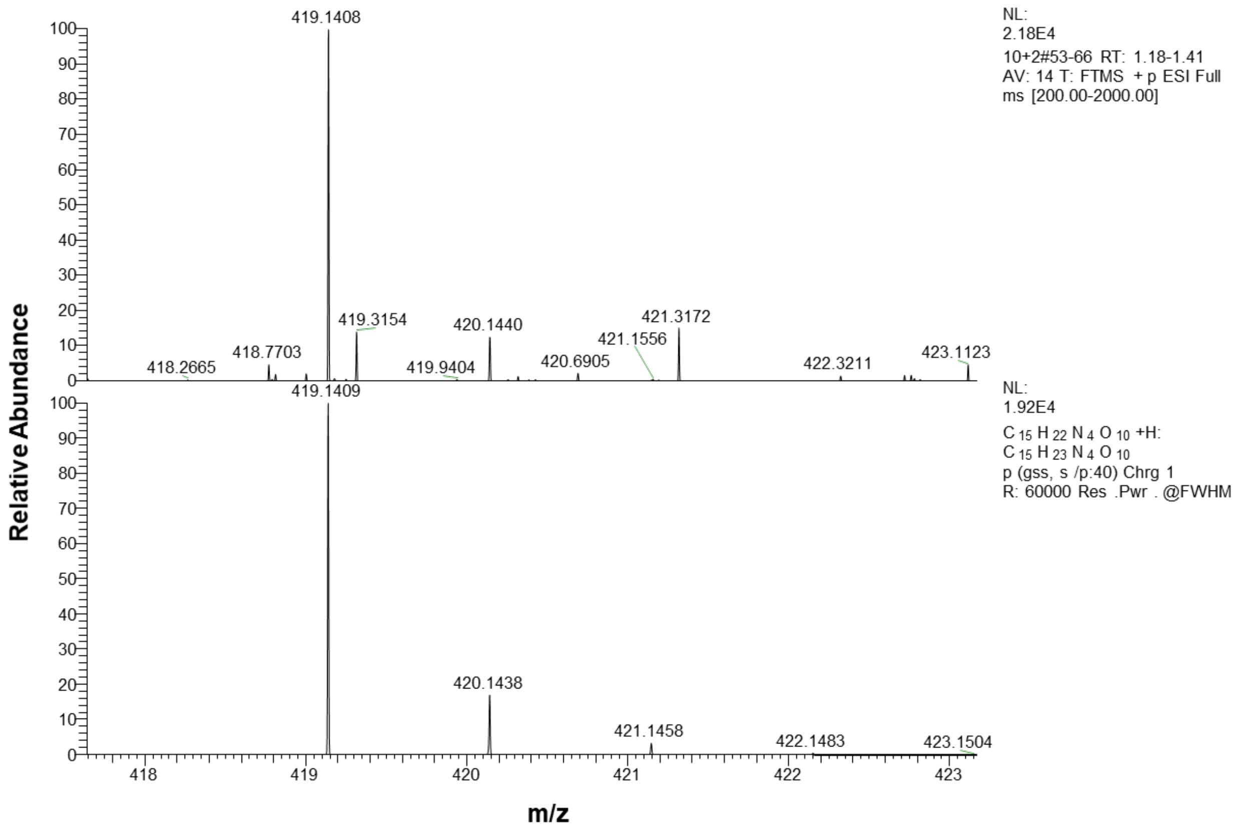

(+)-HR-ESI-MS of **11**, Calc. for $C_{15}H_{22}N_4O_{10}$, expected (M+H)+ ion at m/z = 419.1408, detected: 419.1409.

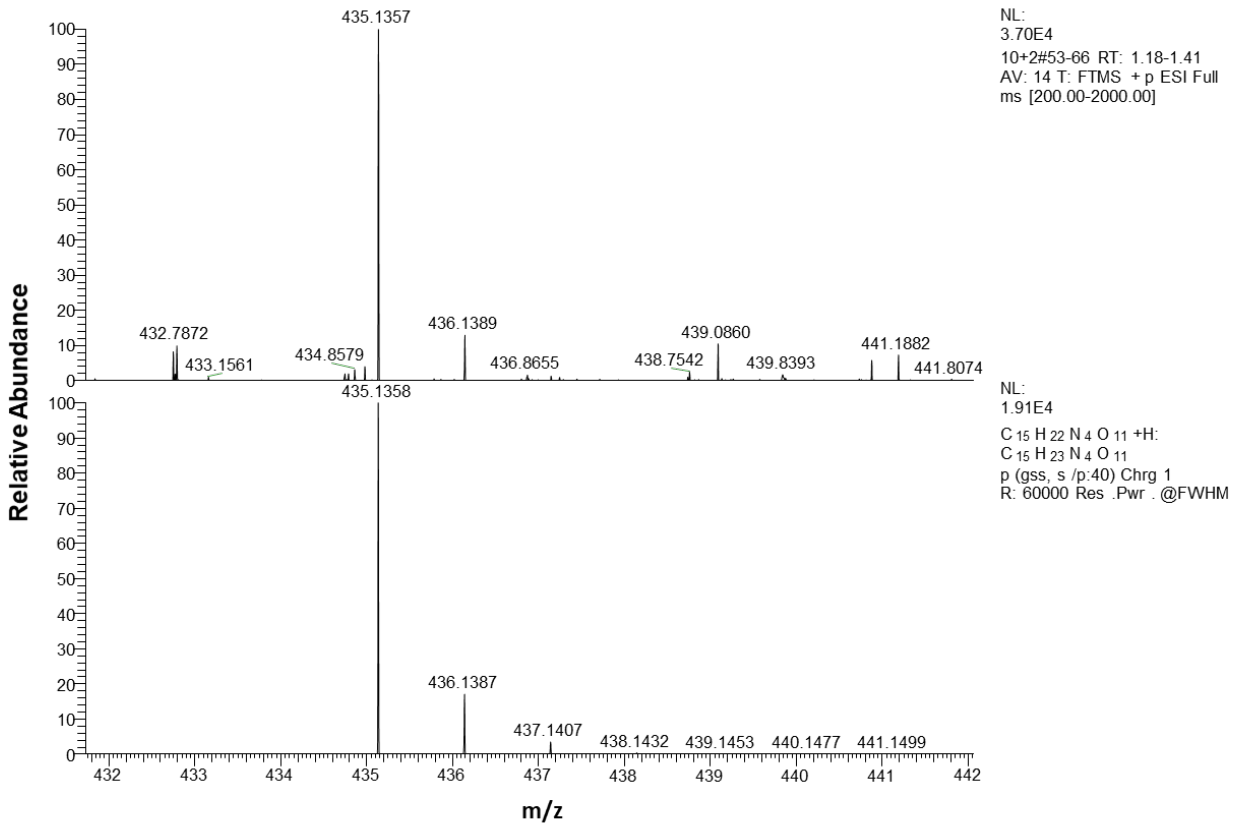

(+)-HR-ESI-MS of **12**, calc. for $C_{15}H_{22}N_4O_{11}$, expected (M+H)+ ion at m/z = 435.1358, detected: 435.1357.

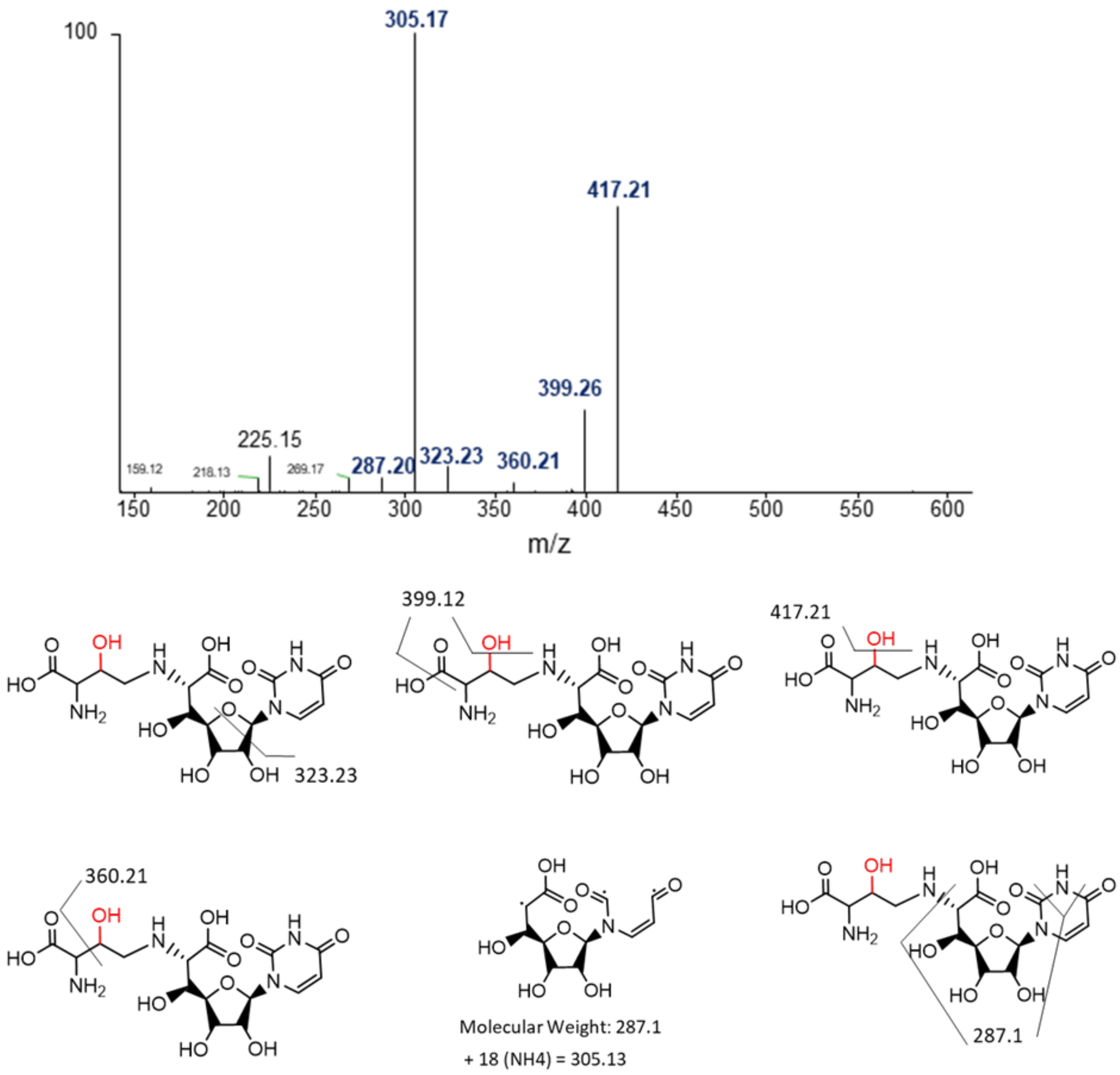

MS/MS of compound **12**.

Supplementary Table 1a. Sequences of primers applied in this study.

Primers	Sequences	Note
cpz 10-F	5'- GTG CCC GGC CGT CGA AAG GCT T-3'	CPZ10
cpz 10-R	5'-TTC GAC TCG CCG TCC GTC CCG AAA A-3'	expression
cpz 10-D72V-F	5'-CGG TGA AGT CGG AGA CCG AGG AGA TCA GC-3'	
cpz 10-D72V-R	5'-GCT GAT CTC CTC GGT CTC CGA CTT CAC CG-3'	
cpz 10-R100V-F	5'-CTT CCG TTC AAG CCC ATT GTC ACG AGG ATC ATG GCG-3'	
cpz 10-R100V-R	5'-CGC CAT GAT CCT CGT GAC AAT GGG CTT GAA CGG AAG-3'	
cpz 10-R102V-F	5'-ATC GAG CGC CAT GAT CAC CGT GCG AAT GGG CTT G-3'	
cpz 10-R102V-R	5'-CAA GCC CAT TCG CAC GGT GAT CAT GGC GCT CGA T-3'	
cpz 10-R121V-F	5'-CGG CGA GGT GGT ATA CCG GTG TCA GGT CAC-3'	
cpz 10-R121V-R	5'-GTG ACC TGA CAC CGG TAT ACC ACC TCG CCG-3'	
cpz 10-D72A-F	5'-CGG TGA AGT CGG AGG CCG AGG AGA TCA GC-3'	
cpz 10-D72A-R	5'-GCT GAT CTC CTC GGC CTC CGA CTT CAC CG-3'	
cpz 10-R100A-F	5'-GCC ATG ATC CTC GTG GCA ATG GGC TTG AAC GGA A-3'	
cpz 10-R100A-R	5'-TTC CGT TCA AGC CCA TTG CCA CGA GGA TCA TGG C-3'	
cpz 10-R102A-F	5'-CGA GCG CCA TGA TCG CCG TGC GAA TGG GCT-3'	
cpz 10-R102A-R	5'-AGC CCA TTC GCA CGG CGA TCA TGG CGC TCG-3'	
cpz 10-R121A-F	5'-CGG CGA GGT GGT ATG CCG GTG TCA GGT CAC-3'	
cpz 10-R121A-R	5'-GTG ACC TGA CAC CGG CAT ACC ACC TCG CCG-3'	
cpz 10-M104L-F	5'-GGA TCG AGC GCC AAG ATC CTC GTG CGA-3'	
cpz 10-M104L-R	5'-TCG CAC GAG GAT CTT GGC GCT CGA TCC-3'	CPZ10
cpz 10-M104IL-F	5'-GAT CGA GCG CTA TGA TCC TCG TGC GAA TGG-3'	site-directed
cpz 10-M104IL-R	5'-CCA TTC GCA CGA GGA TCA TAG CGC TCG ATC-3'	mutagenesis
cpz 10-M104A-F	5'-CTT CGG ATC GAG CGC CGC GAT CCT CGT GCG AAT G-3'	
cpz 10-M104A-R	5'-CAT TCG CAC GAG GAT CGC GGC GCT CGA TCC GAA G-3'	
cpz 10-M104V-F	5'-GGA TCG AGC GCC ACG ATC CTC GTG CGA-3'	
cpz 10-M104V-R	5'-TCG CAC GAG GAT CGT GGC GCT CGA TCC-3'	
cpz 10-R170A-F	5'-AAT GGT GGT GAC GAC ATG GCG ATC CAC ATT GTT TTC GG-3'	
cpz 10-R170A-R	5'-CCG AAA ACA ATG TGG ATC GCC ATG TCG TCA CCA CCA TT-3'	
cpz 10-R170K-F	5'-CCC GAA AAC AAT GTG GAT CTT CAT GTC GTC ACC ACC ATT G-3'	
cpz 10-R170K-R	5'-CAA TGG TGG TGA CGA CAT GAA GAT CCA CAT TGT TTT CGG G-3'	
cpz 10-R170H-F	5'-TCC CGA AAA CAA TGT GGA TAT GCA TGT CGT CAC CAC CAT T-3'	
cpz 10-R170H-R	5'-AAT GGT GGT GAC GAC ATG CAT ATC CAC ATT GTT TTC GGG A-3'	
cpz 10-R170Q-F	5'-TCC CGA AAA CAA TGT GGA TAT GCA TGT CGT CAC CAC CAT T-3'	
cpz 10-R170Q-R	5'-AAT GGT GGT GAC GAC ATG CAT ATC CAC ATT GTT TTC GGG A-3'	
cpz 10-Y112A-F	5'-GTC ACG GTG CAC CGA AGC GCA GTA CTT CGG ATC G-3'	
cpz 10-Y112A-R	5'-CGA TCC GAA GTA CTG CGC TTC GGT GCA CCG TGA C-3'	
cpz 10-H123A-F	5'-GGT GAC GGC GAG GGC GTA TCG CGG TGT C-3'	
cpz 10-H123A-R	5'-GAC ACC GCG ATA CGC CCT CGC CGT CAC C-3'	
cpz 10-H172A-F	5'-CCG TCC CGA AAA CAA TGG CGA TCC GCA TGT CGT CAC-3'	
cpz 10-H172A-R	5'-GTG ACG ACA TGC GGA TCG CCA TTG TTT TCG GGA CGG-3'	
cpz 10-D117G-F	5'-CGC GGT GTC AGG CCA CGG TGC ACC G-3'	
cpz 10-D117G-R	5'-CGG TGC ACC GTG GCC TGA CAC CGC G-3'	
cpz 10-D117A-F	5'-CGC GGT GTC AGG GCA CGG TGC ACC G-3'	
cpz 10-D117A-R	5'-CGG TGC ACC GTG CCC TGA CAC CGC G-3'	
cpz 15-F	5'-GAA TTC ATG CAA CTG ATG AAG TCG AGT TAC ACG GAT CTC CCC-3'	CPZ15
cpz 15-R	5'-AAG CTT TCA TGA CGT GTT CTT CGG GGC GTT GGT GT-3'	expression
cpz 15-R259A-F	5'-GCG CAT GAG CAG GGC GGG ACT TTG CGG G-3'	CPZ15
cpz 15-R259A-R	5'-CCC GCA AAG TCC CGC CCT GCT CAT GCG C-3'	site-directed
		mutagenesis
cpz 27-NdeI-F	5'-ATA CAA CAT ATG GTG ACC AGG ACG GAG AAG CCC TT-3'	CPZ27
cpz 27-R	5'-TCA TTA TAC AAG CTT TCA GGC CTT TCC GGT GCG C-3'	expression
cpz 12-NdeI-F	5'-ATA CAA CAT ATG GTG ATC ATC GTC GTC ACG GGC C-3'	CPZ12
cpz 12-R	5'-TCA TTA TAC AAG CTT TCA TGT GGC TGC CTC CGC C-3'	expression

Supplementary Table 1b. Activity/solubility/functions of mutants made in this study.

Primers	Activity/solubility	Functions
Cpz10-D72V	soluble - inactive	substrate recognition
Cpz10-R100V	soluble - inactive	substrate recognition
Cpz10-R102V	soluble - inactive	substrate recognition
Cpz10-R121V	soluble - inactive	substrate recognition
Cpz10-D72A	soluble - inactive	substrate recognition
Cpz10-R100A	soluble - inactive	substrate recognition
Cpz10-R102A	soluble - inactive	substrate recognition
Cpz10-R121A	soluble - inactive	substrate recognition
Cpz10-M104L	insoluble	substrate recognition
Cpz10-M104IL	insoluble	substrate recognition
Cpz10-M104A	insoluble	substrate recognition
Cpz10-M104V	insoluble	substrate recognition
Cpz10-R170A	soluble - inactive	α KG recognition/electron transfering pathway
Cpz10-R170K	soluble - inactive	α KG recognition/electron transfering pathway
Cpz10-R170H	soluble - inactive	α KG recognition/electron transfering pathway
Cpz10-R170Q	soluble - inactive	α KG recognition/electron transfering pathway
Cpz10-Y112A	soluble - ND	substrate recognition/electron transfering pathway
Cpz10-H123A	insoluble	tunel/electron transfering pathway
Cpz10-H172A	insoluble	Fe2 chelating ligand/electron transfering pathway
Cpz10-D117G	soluble - ND	Fe1 chelating ligand
Cpz10-D117A	soluble - ND	Fe1 chelating ligand

ND: not detected

Supplementary Table 2. NBO analysis for Fe1. Detection of charge-transfer interactions derived from overlapping LP orbitals of the oxygen and nitrogen atoms of α-KG, Wt91, His115, Asp117, and His160 with LP* orbitals of the Fe (III) bound to Cpz10. NBO analyses were carried out for both IS and HS states of the Fe (III) by using UM06-2X functional in conjunction with the standard basis sets of 6-31G** (for C H N O atoms) and Lanl2dz (for the Fe (III) cation).

Spin state	Donor			Acceptor			$E^{(2)}$ (kJ/mol)	$q_{n_i \to n^*_{Fe}}$ (e)
	n_i orbital	Occupancy (e)	Hybrid	n_j* orbital	Occupancy (e)	Hybrid		
IS	LP(1) Nε2 (His115)	0.8187	$sp^{99.99}d^{0.29}$	LP*(9) Fe	0.0470	$sp^{28.84}d^{0.13}$	5.19	0.0076
HS		0.8189	$sp^{99.99}d^{0.60}$				4.81	0.0072
IS	LP(1) Oδ2 (Asp117)	0.9666	$sp^{2.41}$	LP*(6) Fe	0.1109	$sp^{0.19}d^{0.01}$	22.89	0.0181
HS							22.80	
IS				LP*(7) Fe	0.0837	$sp^{25.26}d^{0.03}$	28.12	0.0302
HS						$sp^{25.68}d^{0.03}$	28.28	
IS	LP(2) Oδ2 (Asp117)	0.9092	$sp^{3.13}$	LP*(6) Fe	0.1109	$sp^{0.19}d^{0.01}$	84.27	0.0638
HS							84.01	0.0631
IS				LP*(7) Fe	0.0837	$sp^{25.26}d^{0.03}$	56.86	0.0596
HS						$sp^{25.68}d^{0.03}$	57.15	
IS	LP(1) Nε2 (His160)	0.8223	p	LP*(8) Fe	0.0593	$sp^{10.05}d^{0.07}$	5.61	0.0079
HS						$sp^{10.08}d^{0.07}$	5.52	0.0075
IS	LP(1) O2 (α-KG)	0.9686	$sp^{1.23}$	LP*(6) Fe	0.1109	$sp^{0.19}d^{0.01}$	25.19	0.0179
HS		0.9687					25.15	
IS		0.9686		LP*(8) Fe	0.0593	$sp^{10.05}d^{0.07}$	24.60	0.0196
HS		0.9687				$sp^{10.08}d^{0.07}$		
IS	LP(2) O2 (α-KG)	0.9210	$sp^{6.60}$	LP*(6) Fe	0.1109	$sp^{0.19}d^{0.01}$	73.76	0.0624
HS			$sp^{6.59}$				73.81	
IS			$sp^{6.60}$	LP*(8) Fe	0.0593	$sp^{0.19}d^{0.01}$	27.03	0.0273
HS			$sp^{6.59}$				27.11	
IS	LP(1) O4 (α-KG)	0.9718	$sp^{2.03}$	LP*(7) Fe	0.0837	$sp^{25.26}d^{0.03}$	41.38	0.0392
HS		0.9722	$sp^{2.09}$			$sp^{25.68}d^{0.03}$	39.96	0.0388
IS	LP(2) O4 (α-KG)	0.9270	$sp^{3.64}$	LP*(6) Fe	0.1109	$sp^{0.19}d^{0.01}$	25.94	0.0186
HS		0.9278	$sp^{3.51}$				26.02	0.0189
IS		0.9270	$sp^{3.64}$	LP*(7) Fe	0.0837	$sp^{25.26}d^{0.03}$	115.19	0.1104
HS		0.9278	$sp^{3.51}$			$sp^{25.68}d^{0.03}$	115.90	0.1116
IS		0.9270	$sp^{3.64}$	LP*(8) Fe	0.0593	$sp^{10.05}d^{0.07}$	5.36	0.0045
HS		0.9278	$sp^{3.51}$			$sp^{10.08}d^{0.07}$	5.40	
IS	LP(1) O Wt91	0.9875	$sp^{21.44}$	LP*(7) Fe	0.0837	$sp^{25.26}d^{0.03}$	5.27	0.0065
HS			$sp^{21.42}$			$sp^{25.68}d^{0.03}$		
IS			$sp^{21.44}$	LP*(9) Fe	0.0470	$sp^{28.84}d^{0.13}$	5.36	0.0057
HS			$sp^{21.42}$			$sp^{28.68}d^{0.13}$		
IS	LP(2) O Wt91	0.9768	$sp^{1.71}$	LP*(6) Fe	0.1109	$sp^{0.19}d^{0.01}$	7.91	0.0058
HS							7.95	
IS				LP*(7) Fe	0.0837	$sp^{25.26}d^{0.03}$	16.19	0.0156
HS						$sp^{25.68}d^{0.03}$		
IS				LP*(8) Fe	0.0593	$sp^{10.05}d^{0.07}$	9.92	0.0080
HS						$sp^{10.08}d^{0.07}$		
IS				LP*(9) Fe	0.0470	$sp^{28.84}d^{0.13}$	33.14	0.0273
HS						$sp^{28.68}d^{0.13}$	33.10	

Supplementary Table 3. NBO analysis for Fe2. Charge-transfer interactions arising from attractive interactions between LP* orbitals of the Fe (II) and LP orbitals of the oxygen and carbon atoms of Ala132, Ile145, Wt56, and Wt89 calculated for both LS and IS states of the Fe (II) by using UM06-2X functional in conjunction with the standard basis sets of 6-31G** (for C H N O atoms) and Lanl2dz (for the Fe (II) cation).

Spin state	Donor			Acceptor			$E^{(2)}$ (kJ/mol)	$q_{n_i \rightarrow n^*_{Fe}}$ (e)
	n_i orbital	Occupancy (e)	Hybrid	n_j* orbital	Occupancy (e)	Hybrid		
LS	LP(1) O (Ala132)	0.9840	$sp^{0.77}$	LP*(6) Fe	0.0246	$sp^{31.76}d^{0.14}$	8.49	0.0074
IS		0.9840	$sp^{0.77}$	LP*(5) Fe	0.0243	$sp^{37.54} d^{0.63}$	8.41	0.0076
LS	LP(1) Cδ1	0.9570	$sp^{4.19}$	LP*(6) Fe	0.0246	$sp^{31.76}d^{0.14}$	5.56	0.0114
IS	(Ile145)	0.9616	$sp^{4.17}$	LP*(5) Fe	0.0243	$sp^{37.54} d^{0.63}$	5.52	0.0116
LS	LP(2) O Wt56	0.9870	P	LP*(5) Fe	0.0580	$sp^{0.06}$	5.65	0.0121
IS		0.9894	$sp^{82.49}$	LP*(4) Fe	0.0520	$sp^{0.07}$	4.81	0.0106
LS	LP(2) O Wt89	0.9722	P	LP*(5) Fe	0.0580	$sp^{0.06}$	12.09	0.0277
IS		0.9724	P	LP*(4) Fe	0.0520	$sp^{0.07}$	13.22	0.0302

Schemes

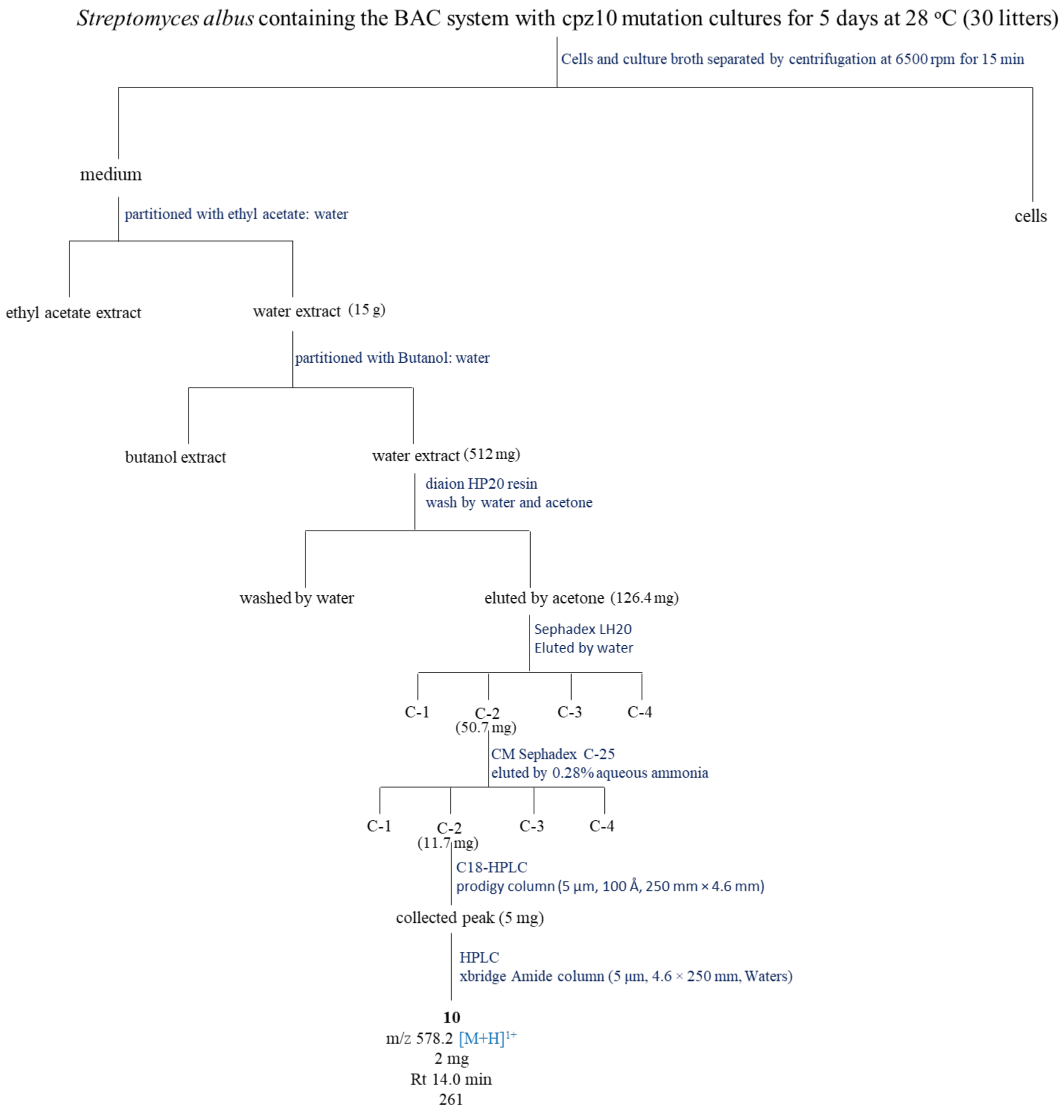

Supplementary Scheme 1. The protocol for compound **13** purification procedure.

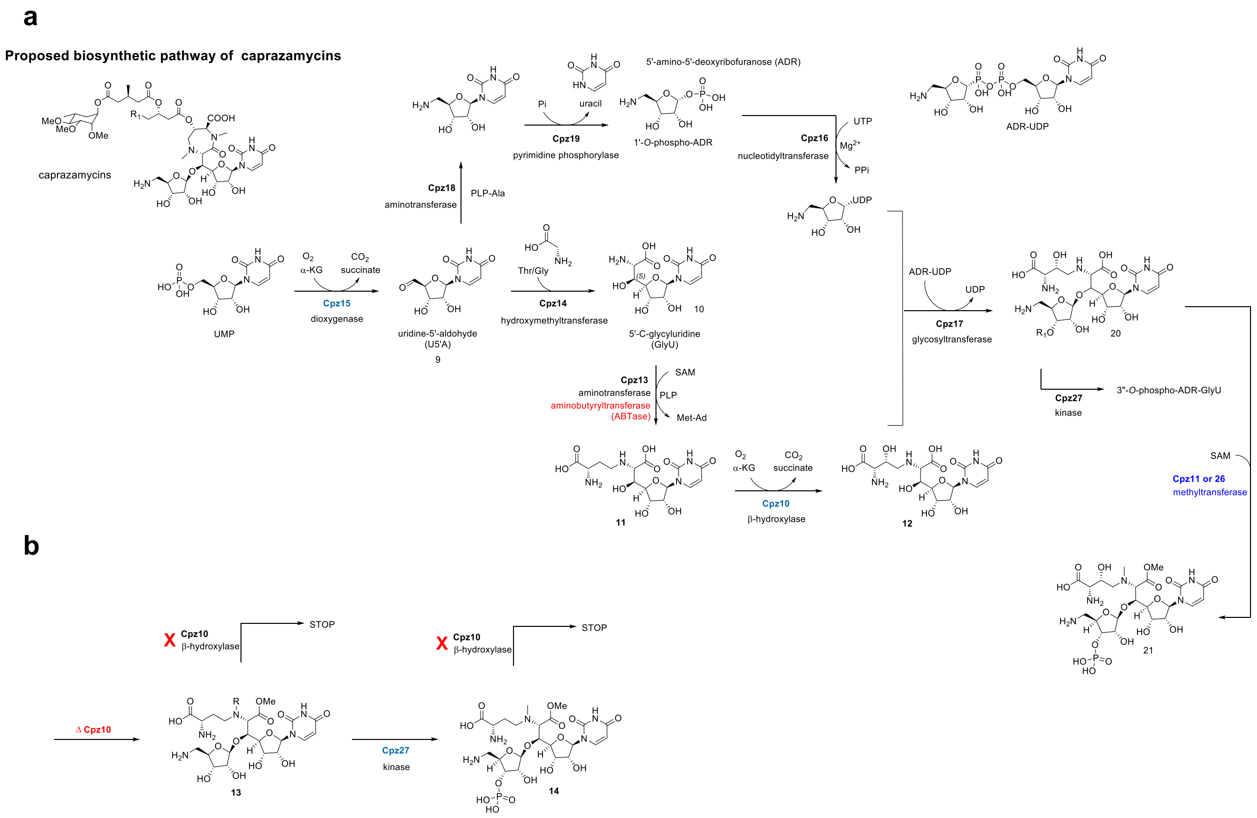

Supplementary Scheme 2. Biosynthetic pathways of caprazamycins. a, The biosynthetic pathway leading to the compound **11** of caprazamycin. **b**, The knock-out intermediate compound **13** is not accepted by Cpz10; compound **14** that is phosphorylated by Cpz27 cannot be hydroxylated by Cpz10.

a

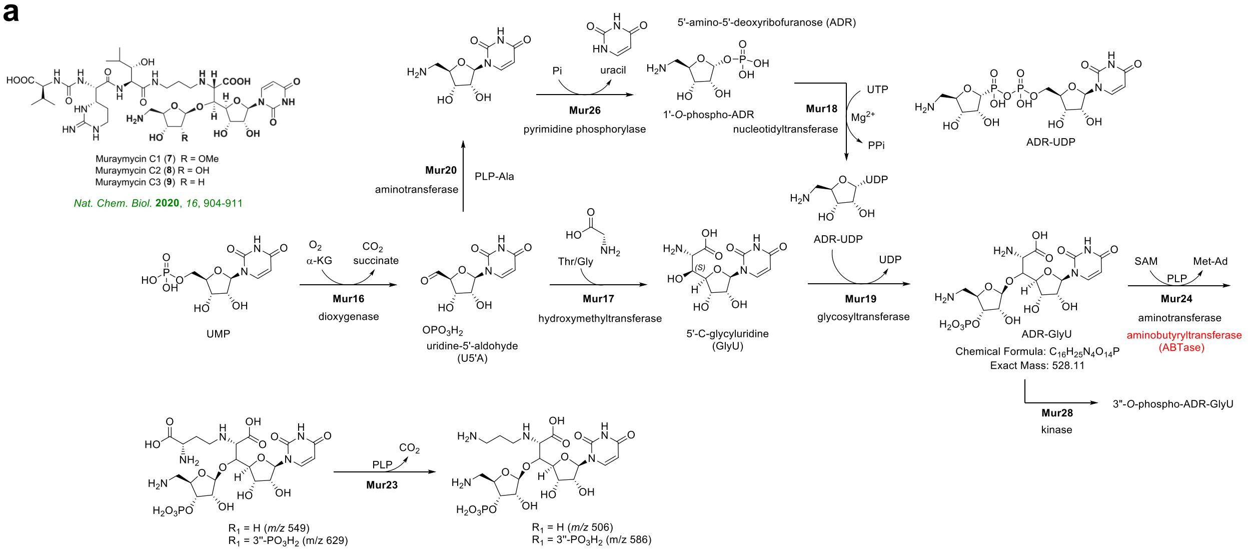

b

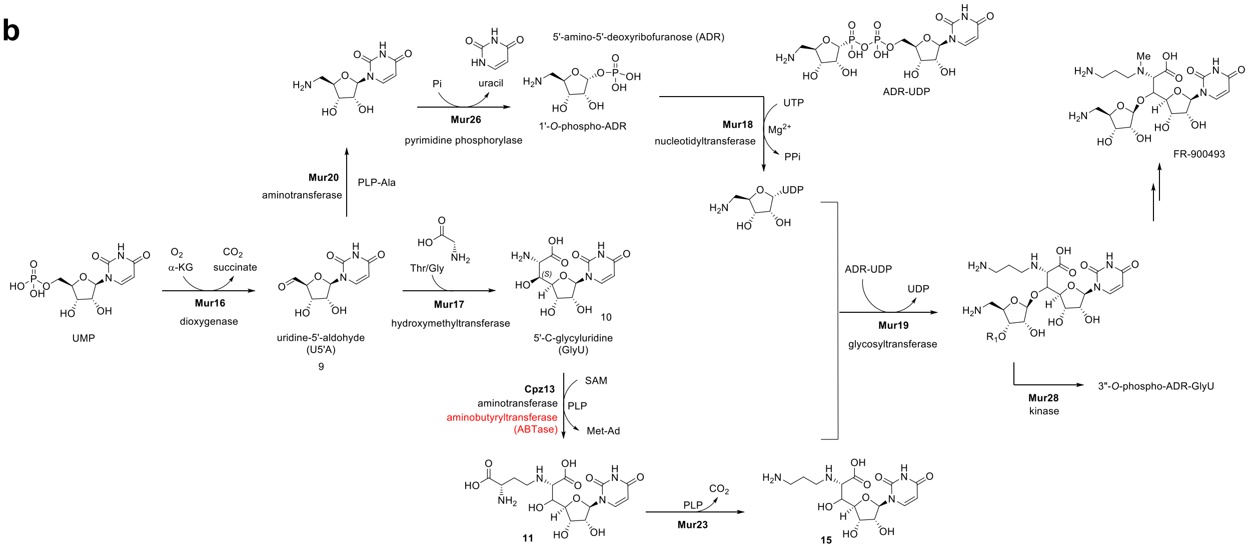

Supplementary Scheme 3. Biosynthetic pathways of 6 and 7. a, The biosynthetic steps leading to the compound **15** of muraymycins is presented.[4] **b**, The re-proposed pathway which is based on our results highlights the difference with regard to the second sugar addition.

Reference

1 Hirano, S., Ichikawa, S. & Matsuda, A. Development of a highly β-selective ribosylation reaction without using neighboring group participation: total synthesis of (+)-caprazol, a core structure of caprazamycins. *J. Org. Chem.* **72**, 9936-9946 (2007).

2 Schrödinger, L. The PyMOL Molecular Graphics System, Version 2.0 Schrödinger, LLC (2017). *Google Scholar There is no corresponding record for this reference*.

3 Volkamer, A., Kuhn, D., Grombacher, T., Rippmann, F. & Rarey, M. Combining global and local measures for structure-based druggability predictions. *J. Chem. Inf. Model.* **52**, 360-372 (2012).

4 Cui, Z. *et al.* Pyridoxal-5 ́ -phosphate-dependent alkyl transfer in nucleoside antibiotic biosynthesis. *Nature Chemical Biology*, 1-8 (2020).
