# Supplementary material for: β-Hydroxylation of α-amino-β-hydroxylbutanoyl-glycyluridine catalyzed by a nonheme hydroxylase ensures the maturation of caprazamycin
Source: Commun Chem. 2022 Jul 28;5:87. doi: 10.1038/s42004-022-00703-6 (PMC9814697; doi:10.1038/s42004-022-00703-6)
Supplement: Supplementary file 2 — Description of Additional Supplementary Files [file 42004_2022_703_MOESM2_ESM.pdf]

Description of Additional Supplementary Files

File name: Supplementary Movie 1

Description: Dynamic movement of Fe2 relative to Fe1 in the active site of Cpz10 by MD simulation. Trajectory frames (DCD file) that were generated during a MD simulation (using NAMD software) have 1000 frames equal to 10 nano seconds and 10 million steps. Each frame is a single protein conformation.

File name: Supplementary Movie 2

Description: Slow motion and zoom-in movement of Fe2 relative to Fe1 in the active site of Cpz10.

File name: Supplementary Data 1

Description: protein-ligand initial state

File name: Supplementary Data 2

Description: protein-ligand final state

File name: Supplementary Data 3

Description: DFT Coordinates
