# Supplementary material for: β-Hydroxylation of α-amino-β-hydroxylbutanoyl-glycyluridine catalyzed by a nonheme hydroxylase ensures the maturation of caprazamycin
Source: Commun Chem. 2022 Jul 28;5:87. doi: 10.1038/s42004-022-00703-6 (PMC9814697; doi:10.1038/s42004-022-00703-6)
Supplement: Supplementary file 6 — Supplementary data 3 [file 42004_2022_703_MOESM6_ESM.pdf]

DFT Coordinates

Input files for Fe1. Input file of the Cpz10 ternary structure, containing the initial atomic coordinates of the active site residues in addition with αKG and first iron (Fe1) for Gaussian [4] calculation. The number -1 signifies atoms that their coordinates were remained fixed, whereas number 0 implies hydrogen atoms that their positions were relaxed during the geometry optimization.

1 6

N	-1	10.69500000	12.44800000	-19.79400000
C	-1	12.15200000	12.55600000	-19.75600000
C	-1	12.75100000	11.46700000	-18.87300000
O	-1	11.99700000	10.71570000	-18.20170000
C	-1	12.58300000	13.92300000	-19.22400000
C	-1	12.23000000	14.12400000	-17.78600000
N	-1	10.94000000	14.39000000	-17.37100000
C	-1	12.97800000	14.03900000	-16.65900000
C	-1	10.91400000	14.47500000	-16.05200000
N	-1	12.13800000	14.27000000	-15.59600000
N	-1	15.99800000	11.08500000	-15.91300000
C	-1	16.98400000	11.73500000	-15.06600000
C	-1	18.20500000	10.83900000	-14.92700000
O	-1	18.14970000	9.63800000	-15.29850000
C	-1	16.40700000	12.05200000	-13.68800000
C	-1	15.25800000	13.04100000	-13.75600000
O	-1	15.45800000	14.21300000	-14.14300000
O	-1	14.14800000	12.63900000	-13.41300000
N	-1	15.25500000	8.78800000	-7.45700000
C	-1	14.91300000	9.79200000	-6.46100000
C	-1	13.85100000	9.23900000	-5.53500000
O	-1	13.08380000	8.32430000	-5.93270000
C	-1	14.37600000	11.07800000	-7.10800000
C	-1	15.37300000	11.76200000	-8.00500000
C	-1	14.72900000	12.79900000	-8.88400000
N	-1	15.76100000	13.55700000	-9.58200000
C	-1	15.52600000	14.64700000	-10.30300000
N	-1	16.53100000	15.26100000	-10.91000000
N	-1	14.28800000	15.12000000	-10.41600000
N	-1	2.26900000	10.16800000	-11.07300000
C	-1	3.58600000	9.96200000	-11.64100000
C	-1	3.43400000	8.83800000	-12.65600000
O	-1	2.75620000	7.81760000	-12.36810000
C	-1	4.55200000	9.62300000	-10.50100000
C	-1	5.97800000	9.53500000	-10.90000000
C	-1	6.46100000	8.39600000	-11.52900000
C	-1	6.86100000	10.57000000	-10.60200000
C	-1	7.81100000	8.30300000	-11.89700000
C	-1	8.20300000	10.48300000	-10.95900000
C	-1	8.67600000	9.34100000	-11.60500000
N	-1	9.69900000	8.90500000	-17.85700000
C	-1	8.60300000	9.24800000	-16.95800000
C	-1	8.16900000	10.69800000	-17.15600000
O	-1	8.89630000	11.48860000	-17.81140000
C	-1	9.00200000	9.03000000	-15.47900000
C	-1	10.05500000	9.97500000	-15.00000000
N	-1	11.39500000	9.79000000	-15.27200000

C	-1	9.96800000	11.13200000	-14.29700000
C	-1	12.08800000	10.78500000	-14.74600000
N	-1	11.24600000	11.60900000	-14.14500000
C	-1	11.40800000	13.33200000	-10.82000000
C	-1	10.33800000	14.04500000	-11.64500000
C	-1	9.04300000	14.37600000	-10.91000000
C	-1	9.33800000	15.55800000	-9.97500000
C	-1	8.06000000	15.90500000	-9.24700000
O	-1	11.22000000	13.03700000	-9.60500000
O	-1	12.49600000	13.08400000	-11.36900000
O	-1	7.25700000	14.97400000	-9.08500000
O	-1	10.51800000	14.34600000	-12.82600000
O	-1	7.86400000	17.10100000	-8.98100000
Fe	-1	12.28200000	13.55400000	-13.45200000
O	-1	13.16100000	15.47000000	-13.10100000
H	0	12.01540000	14.64600000	-19.77180000
H	0	13.65190000	13.93500000	-19.27070000
H	0	14.02630000	13.83100000	-16.60660000
H	0	12.40880000	14.28120000	-14.63340000
H	0	10.04910000	14.67570000	-15.45480000
H	0	12.51000000	12.43830000	-20.75740000
H	0	10.31380000	12.77630000	-18.92980000
O	0	14.16960000	11.29930000	-18.80770000
H	0	17.18800000	12.54010000	-13.14340000
H	0	15.99070000	11.13680000	-13.32190000
H	0	17.26670000	12.65960000	-15.52440000
H	0	15.11650000	11.54660000	-15.81310000
O	0	19.41590000	11.36130000	-14.37410000
H	0	14.19270000	11.75730000	-6.30180000
H	0	13.57130000	10.77240000	-7.74350000
H	0	15.75060000	11.00670000	-8.66210000
H	0	16.04130000	12.29190000	-7.35890000
H	0	14.22790000	13.48590000	-8.23440000
H	0	14.17630000	12.26950000	-9.63170000
H	0	16.70540000	13.23550000	-9.51330000
H	0	17.46080000	14.90320000	-10.82390000
H	0	16.35970000	16.08230000	-11.45430000
H	0	13.53110000	14.65450000	-9.95730000
H	0	14.11410000	15.94110000	-10.95970000
H	0	15.80530000	10.03080000	-5.92100000
H	0	14.47670000	8.64670000	-8.06880000
O	0	13.71960000	9.75600000	-4.20820000
H	0	4.28200000	8.63960000	-10.17710000
H	0	4.49360000	10.44570000	-9.81930000
H	0	5.79770000	7.58200000	-11.73490000
H	0	6.50370000	11.44090000	-10.09330000
H	0	8.17050000	7.43110000	-12.40240000
H	0	8.87020000	11.28990000	-10.73860000
H	0	9.70890000	9.26830000	-11.87470000
H	0	3.98450000	10.82330000	-12.13530000
H	0	2.03360000	11.13870000	-11.12050000
O	0	4.06150000	8.94150000	-13.93680000
H	0	9.44060000	8.05510000	-15.43430000
H	0	8.12700000	9.24990000	-14.90380000
H	0	9.07180000	11.58700000	-13.93010000
H	0	11.49960000	12.44490000	-13.65820000

H	0	13.15010000	10.90370000	-14.79770000
H	0	7.78480000	8.60040000	-17.19470000
H	0	10.20660000	8.99650000	-17.00030000
O	0	6.93590000	11.15800000	-16.59670000
H	0	8.80490000	13.53510000	-10.29270000
H	0	8.34150000	14.71810000	-11.64190000
H	0	9.58490000	16.39510000	-10.59410000
H	0	10.03480000	15.21200000	-9.24040000
H	0	13.91210000	16.06790000	-13.10100000
H	0	13.47430000	14.56260000	-13.10100000
C	0	12.95146967	9.04254755	-3.23564594
H	0	13.03748304	9.52893926	-2.28647535
H	0	11.92449238	9.02463554	-3.53547389
H	0	13.31767900	8.04022599	-3.15727361
C	0	15.58546304	7.52333205	-6.78446067
H	0	14.71871434	6.89648920	-6.75772635
H	0	16.36755400	7.02785999	-7.32087578
H	0	15.91066182	7.72510675	-5.78524461
C	0	20.53665795	10.75140671	-15.01972511
H	0	21.43084874	11.27413981	-14.75126104
H	0	20.61409483	9.73001770	-14.71042267
H	0	20.40363902	10.79370913	-16.08058159
C	0	16.43281855	11.15732276	-17.31535596
H	0	16.82053877	10.20707523	-17.61801529
H	0	15.59924314	11.41597491	-17.93434798
H	0	17.19517420	11.90156124	-17.41446669
C	0	10.17199377	13.26176124	-20.90086816
H	0	9.33175252	12.76690651	-21.34133771
H	0	9.86791088	14.21755068	-20.52817507
H	0	10.93562650	13.39315589	-21.63877093
C	0	14.64185651	10.74964819	-20.04048412
H	0	14.78596826	11.53732692	-20.75019816
H	0	15.57080270	10.24555883	-19.87359225
H	0	13.92216514	10.05478104	-20.42009442
C	0	10.55082233	7.71024384	-17.94563656
H	0	10.37604437	7.21599987	-18.87841497
H	0	11.57897413	7.99976899	-17.88255666
H	0	10.31748216	7.04531059	-17.14045576
C	0	6.52479214	12.35252245	-17.26677942
H	0	6.51002002	12.18192366	-18.32298860
H	0	5.54506191	12.62775156	-16.93620775
H	0	7.21168246	13.14169530	-17.04252978
C	0	2.26694205	9.73141982	-9.66932856
H	0	2.89537732	10.37839382	-9.09366047
H	0	1.26889684	9.76942319	-9.28545160
H	0	2.63505403	8.72865968	-9.60715402
C	0	3.22155117	8.34523441	-14.92869379
H	0	3.60751230	8.56538550	-15.90207322
H	0	3.19806235	7.28509091	-14.78571113
H	0	2.23058589	8.73907025	-14.84048298

Input files for Fe2. Input file of the Cpz10 ternary structure, containing the initial atomic coordinates of the active site residues in addition with water molecules and second iron (Fe2) for Gaussian calculation. The number -1 signifies atoms that their coordinates were remained fixed, whereas number 0 implies hydrogen atoms that their positions were relaxed during the geometry optimization.

```
2 1
 N         -1   15.25500000    8.78800000   -7.45700000
 C         -1   14.91300000    9.79200000   -6.46100000
 C         -1   13.85100000    9.23900000   -5.53500000
 O         -1   13.08380000    8.32430000   -5.93270000
 C         -1   14.37600000   11.07800000   -7.10800000
 C         -1   15.37300000   11.76200000   -8.00500000
 C         -1   14.72900000   12.79900000   -8.88400000
 N         -1   15.76100000   13.55700000   -9.58200000
 C         -1   15.52600000   14.64700000  -10.30300000
 N         -1   16.53100000   15.26100000  -10.91000000
 N         -1   14.28800000   15.12000000  -10.41600000
 N         -1   -3.05700000   14.18800000   -8.93200000
 C         -1   -1.60800000   14.13600000   -9.12200000
 C         -1   -1.18500000   12.69500000   -9.35700000
 O         -1   -1.57100000   11.80100000   -8.59400000
 C         -1   -0.86500000   14.69800000   -7.90200000
 N         -1   -0.40400000   12.46900000  -10.42000000
 C         -1    0.12400000   11.14400000  -10.71400000
 C         -1    1.54200000   11.25300000  -11.25000000
 O         -1    1.95200000   12.26800000  -11.80600000
 C         -1   -0.72000000   10.36700000  -11.74200000
 C         -1   -2.20100000   10.25300000  -11.39200000
 C         -1   -2.46000000    9.30200000  -10.21300000
 N         -1   -3.78200000    9.58600000   -9.63600000
 C         -1   -4.61400000    8.66500000   -9.17400000
 N         -1   -4.28000000    7.38500000   -9.19700000
 N         -1   -5.79100000    9.03400000   -8.68200000
 N         -1    2.26900000   10.16800000  -11.07300000
 C         -1    3.58600000    9.96200000  -11.64100000
 C         -1    3.43400000    8.83800000  -12.65600000
 O         -1    2.75620000    7.81760000  -12.36810000
 C         -1    4.55200000    9.62300000  -10.50100000
 C         -1    5.97800000    9.53500000  -10.90000000
 C         -1    6.46100000    8.39600000  -11.52900000
 C         -1    6.86100000   10.57000000  -10.60200000
 C         -1    7.81100000    8.30300000  -11.89700000
 C         -1    8.20300000   10.48300000  -10.95900000
 C         -1    8.67600000    9.34100000  -11.60500000
 N         -1    1.52300000    9.25800000   -5.47200000
 C         -1    2.34400000    9.31900000   -4.26700000
 C         -1    1.53800000   10.04100000   -3.18800000
 O         -1    1.38650000   11.28890000   -3.24510000
 C         -1    3.68600000   10.00700000   -4.53000000
 C         -1    4.48500000    9.16900000   -5.53600000
 C         -1    4.47100000   10.19200000   -3.20200000
 C         -1    5.67100000    9.87600000   -6.13900000
 C         -1   11.40800000   13.33200000  -10.82000000
 C         -1   10.33800000   14.04500000  -11.64500000
 C         -1    9.04300000   14.37600000  -10.91000000
 C         -1    9.33800000   15.55800000   -9.97500000
```

C	-1	8.06000000	15.90500000	-9.24700000
O	-1	11.22000000	13.03700000	-9.60500000
O	-1	12.49600000	13.08400000	-11.36900000
O	-1	7.25700000	14.97400000	-9.08500000
O	-1	10.51800000	14.34600000	-12.82600000
O	-1	7.86400000	17.10100000	-8.98100000
Fe	-1	1.94300000	11.70100000	-7.60300000
O	-1	2.50900000	14.19300000	-9.36100000
O	-1	1.30700000	13.11400000	-5.15300000
H	0	14.19270000	11.75730000	-6.30180000
H	0	13.57130000	10.77240000	-7.74350000
H	0	15.75060000	11.00670000	-8.66210000
H	0	16.04130000	12.29190000	-7.35890000
H	0	14.22790000	13.48590000	-8.23440000
H	0	14.17630000	12.26950000	-9.63170000
H	0	16.70540000	13.23550000	-9.51330000
H	0	13.53110000	14.65450000	-9.95730000
H	0	14.11410000	15.94110000	-10.95970000
H	0	17.46080000	14.90320000	-10.82390000
H	0	16.35970000	16.08230000	-11.45430000
H	0	15.80530000	10.03080000	-5.92100000
H	0	14.47670000	8.64670000	-8.06880000
O	0	13.71960000	9.75600000	-4.20820000
H	0	-0.25950000	15.52690000	-8.20390000
H	0	-1.57510000	15.02270000	-7.17040000
H	0	-0.24260000	13.93610000	-7.48130000
H	0	-1.35620000	14.73770000	-9.97020000
H	0	-3.49070000	13.47140000	-9.47820000
H	0	-0.34200000	9.36610000	-11.72880000
H	0	-0.67950000	10.94240000	-12.64320000
H	0	-2.67310000	9.80670000	-12.24220000
H	0	-2.50310000	11.22470000	-11.06110000
H	0	-1.73710000	9.53890000	-9.46050000
H	0	-2.50590000	8.31280000	-10.61830000
H	0	-4.07340000	10.54150000	-9.58910000
H	0	-3.39310000	7.10620000	-9.56530000
H	0	-4.91450000	6.69630000	-8.84600000
H	0	-6.04360000	10.00140000	-8.66220000
H	0	-6.42470000	8.34450000	-8.33130000
H	0	0.09860000	10.60510000	-9.79000000
H	0	0.41040000	13.02260000	-10.24590000
H	0	4.28200000	8.63960000	-10.17710000
H	0	4.49360000	10.44570000	-9.81930000
H	0	5.79770000	7.58200000	-11.73490000
H	0	6.50370000	11.44090000	-10.09330000
H	0	8.17050000	7.43110000	-12.40240000
H	0	8.87020000	11.28990000	-10.73860000
H	0	9.70890000	9.26830000	-11.87470000
H	0	3.98450000	10.82330000	-12.13530000
H	0	1.71830000	9.44730000	-11.49410000
O	0	4.06150000	8.94150000	-13.93680000
H	0	3.52000000	10.98060000	-4.94160000
H	0	5.16780000	10.99700000	-3.30870000
H	0	3.78600000	10.41570000	-2.41100000
H	0	4.99980000	9.29070000	-2.97170000
H	0	3.81670000	8.97990000	-6.34990000

H	0	4.88960000	8.35250000	-4.97520000
H	0	6.65350000	9.53120000	-6.38540000
H	0	6.05040000	9.69560000	-5.15490000
H	0	5.43190000	9.20960000	-6.94120000
H	0	2.58400000	8.32910000	-3.93920000
H	0	1.93810000	9.82060000	-6.18700000
O	0	0.95590000	9.29850000	-2.11340000
H	0	8.80490000	13.53510000	-10.29270000
H	0	8.34150000	14.71810000	-11.64190000
H	0	9.58490000	16.39510000	-10.59410000
H	0	10.03480000	15.21200000	-9.24040000
H	0	3.26010000	14.79090000	-9.36100000
H	0	2.82230000	13.28560000	-9.36100000
H	0	2.05810000	13.71190000	-5.15300000
H	0	1.62030000	12.20660000	-5.15300000
C	0	-0.30122176	9.88017019	-1.75814702
H	0	-0.82906359	9.21722923	-1.10485362
H	0	-0.13486001	10.81306638	-1.26122805
H	0	-0.88038484	10.04545053	-2.64254090
C	0	1.43391341	7.86539729	-5.93419180
H	0	0.41002133	7.55468147	-5.93501568
H	0	1.82950743	7.79267880	-6.92571457
H	0	1.99736620	7.23516880	-5.27827030
C	0	3.22155117	8.34523441	-14.92869379
H	0	3.67621519	8.45101266	-15.89149781
H	0	3.09118796	7.30638686	-14.70801030
H	0	2.26875739	8.83214714	-14.92875922
C	0	-3.56031974	15.50157045	-9.35873400
H	0	-4.62817392	15.51321802	-9.29201221
H	0	-3.15501122	16.26206071	-8.72447888
H	0	-3.26413591	15.68556853	-10.37032673
C	0	13.67657452	8.67213997	-3.27637585
H	0	13.79673210	9.05091620	-2.28290178
H	0	12.73453115	8.17094650	-3.35549223
H	0	14.46626647	7.98355738	-3.49349479
C	0	15.58546304	7.52333205	-6.78446067
H	0	14.71126816	6.90863778	-6.73122290
H	0	16.34671705	7.01367554	-7.33731018
H	0	15.93894496	7.72714262	-5.79531367
