# Supplementary material for: β-Hydroxylation of α-amino-β-hydroxylbutanoyl-glycyluridine catalyzed by a nonheme hydroxylase ensures the maturation of caprazamycin
Source: Commun Chem. 2022 Jul 28;5:87. doi: 10.1038/s42004-022-00703-6 (PMC9814697; doi:10.1038/s42004-022-00703-6)
Supplement: Supplementary file 3 — Li_PR File [file 42004_2022_703_MOESM3_ESM.pdf]

Peer Review File

β-Hydroxylation for α-amino-β-hydroxylbutanoyl-glycyluridine catalyzed by a nonheme hydroxylase ensures the maturation of caprazamycin

Open Access This file is licensed under a Creative Commons Attribution 4.0 International License, which permits use, sharing, adaptation, distribution and reproduction in any medium or format, as long as you give appropriate credit to the original author(s) and the source, provide a link to the Creative Commons license, and indicate if changes were made. In the cases where the authors are anonymous, such as is the case for the reports of anonymous peer reviewers, author attribution should be to 'Anonymous Referee' followed by a clear attribution to the source work. The images or other third party material in this file are included in the article's Creative Commons license, unless indicated otherwise in a credit line to the material. If material is not included in the article's Creative Commons license and your intended use is not permitted by statutory regulation or exceeds the permitted use, you will need to obtain permission directly from the copyright holder. To view a copy of this license, visit http://creativecommons.org/licenses/by/4.0/.

Reviewers' comments:

Reviewer #1 (Remarks to the Author):

This manuscript reports a biochemical study on two nonheme αKG-dependent enzymes Cpz10 and Cpdz 15 involved in the biosynthesis of caprazamycin. Unlike the previous assignment ofβ-hydroxylase, Cpdz 15 was demonstrated to be the starter enzyme converting uridine mono phosphate to uridine 5'aldehyde, while Cpz10 was found to be the actual β-hydroxylase. In particular, crystal structures together with EPR experiments and NBO calculations tend to support that Cpz10 is an unusual αKG-dependent enzyme with two irons (Fe(III) and Fe(II)). While the active site for O2 activation was proposed to be Fe(III), the Fe(II) was suggested to be an electron donor for the reduction of Fe(III) site. Such finding seems quite uncommon in αKG-dependent non-hem enzymes. I think the work could be publishable, but the following main issues need to be addressed.
1. In Fig.4c, it is seen that Fe2 is only coordinated by two waters. I think such coordination environment is unusual in iron-containing proteins. Could it be an unnatural Fe site during crystallographic characterization/analysis?
2. According to caption of Figure 6B, EPR was performed with ascorbic acid under an aerobic condition. In such condition, the potential Fe(II) in the resting state of αKG-dependent enzyme could be oxidized to be Fe(III) via the formation of FeIIIOO•— species, which might disturb the EPR results. So why not do the experiment in the anaerobic condition like reference 63?
3. For the NBO calculation, Fig S19 has some contradiction with the DFT optimized coordinates in terms of protonation states of His115 and His160. In addition, it looks that neither the structure shown in Fig S19 nor the structure of the DFT coordinates is correct.
4. Page 6, the fifth line in the second paragraph of Discussion. 'Nonheme αKG-dependent enzymes that contain two irons are not uncommon but often ignored63, 64, 65'. Indeed, the reference 63, 64 and 65 did not mention the second iron in the enzymes.

Other small issues:
(1) Page 3, the third line from the bottom in the paragraph before EPR for Cpz10 'This pair of irons may be spatially connected via a hidden electron/charge transfer conduit en route pass points of Wt89, His172, His123, and αKG to Fe1'. Wt89 here should be Wt56 according to Fig. 6A.
(2) Page 3, the fourth line in the paragraph Investigation of the nature of FeIII and FeII EPR signals by NBO analysis 'the valance orbitals containing a total of 6.96 electrons as opposed to the last two orbitals that are Rydberg orbitals with 0.03 electrons'. Here 6.96 should be 6.93.
(3) Page 6, the second line from the bottom in the second paragraph of Discussion. 'On top of that, EPR spectra reflect signal changes for both Fe2 (2.02→1.97 g) and Fe1 (4.75→4.25 g) in the presence or absence of substrates in the Cpz10-mediated reaction'. Values 2.02 and 4.75 in Fig 6B. represent the absence of substrates. So it should be changed to "in the absence or presence of substrates"

Reviewer #2 (Remarks to the Author):

The Manuscript "β-Hydroxylation for α-amino-β-hydroxylbutanoyl-glycyluridine catalysed by a nonheme hydroxylase warrants reaction fidelity toward maturation of caprazamycin" describes the putative function of several enzymes in caprazamycin biosynthesis. The authors mainly focus on the non-heme α-ketoglutarate-dependent ß-hydroxylase Cpz10 to demonstrate, that this enzyme is responsible for ß-hydroxylation of the attached 3-amino-3-carboxypropyl group of a caprazamycin biosynthetic intermediate. These findings were supported by X-ray crystal structures of Cpz10. ß-hydroxylation prior to diazepanone ring formation has been an open question for a long time in liponucleoside research and therefore is of great interest for the natural product community.

However, there are some major issues, which needs to be addressed before publication:

In general, this manuscript is lacking a focus on the biochemical characterization of Cpz10. Instead, preliminary work on other enzymes such as phosphotransferases and the PLP-dependent decarboxylase Mur23 are included, deflecting from the major story and conflicting even with previous results.

In the first section of the results chapter, the authors claimed that a controversy has to be resolved since Cpz10 and Cpz15 both could catalyse the first step in liponucleoside biosynthesis, that is the oxidative dephosphorylation of UMP. However, the van Lanen group has solved this controversy ten years ago by studying the corresponding enzyme LipL from liposidomycin biosynthesis.

The same occurs for Cpz14. Formation of (5'S,6'S)-5'-C-glycyluridine has been shown to be catalyzed by LipK. The new aspect of this manuscript is that Mur17 seems to do the same reaction but does not only rely on threonine as the only aldol donor, but also accepts glycine. Unfortunately, the corresponding ESI-MS data are not presented. Supplemental figure 5 indicates in the legend for c, (i) control (ii) Mur17 + PLP + threonine and (iii) Mur17 + PLP + glycine but c does neither show (i), (ii) or (iii). Only the ESI-MS of compound 10 is shown. Fig 3B only shows the corresponding LC traces.

In the second chapter of the results (Cpz10 is the ß-hydroxylase), the authors claimed that "it has been proposed that the four-carbon sidechain is transferred from SAM to compound 10 by Cpz13…" This statement is not entirely true. Compound 10 lacks the aminoribose (ADR), the actual proposal is that prior to the attachment of C4N, the disaccharide core structure (ADR-GlyU) is formed by the action of Cpz17 (as it has been demonstrated for LipN). With the synthetic compound 11 in hand (reference 14 for the synthesis is wrong), the authors can clearly demonstrate, that the hydroxylated compound 11 is formed, generating compound 12. This is an interesting finding since compound 12 lacks the ADR moiety. This opens the general question, at which time point ADR is attached. Interestingly, a cpz10 deletion mutant only accumulated the ADR-GlyU disaccharide (compound 13). This can only be explained if the ribosyltransferase Cpz17 can transfer ADR onto both, compound 11 and 12. Otherwise, the presented results are not only in disagreement with the biochemical studies on the Cpz17 homologue, LipN, but also with their own findings of the cpz10 knock-out mutants. To clarify the situation, one should consider a cpz17 knock-out. If compound 11 and 12 are accumulated in this mutant, this would be a strong indication for the proposed pathway in Supplementary Scheme 3. In addition, the authors do not explain, why the C7' carboxyl group is methylated. Methylation of the C6' amine could be explained by the action of one of the N-methyltransferases (Cpz11 or 26) prior ß-hydroxylation. In Figure 2, compound 13 and 14 contain a hydroxyl-group at C5" of the ADR moiety (Fig 2B) but they contain the amino group in Fig 2D. I assume that the structure in Fig 2B is wrong.

The last chapter on phosphotransferase activity offers some preliminary results again contradicting previous findings. First, Cpz12 is mentioned in the abstract as an enzyme, which was characterized in this study. Besides the successful expression and purification of the phosphotransferase, no data are presented, thereby Cpz12 should be deleted from this manuscript.

Secondly, the authors could demonstrate, that Cpz27 can phosphorylate compound 13, but not caprazol or caprazamycins. This is not an indication, that phosphorylation occurs before diazepanone ring formation as stated by the authors since aglycones and caprazamycins lacking the 3-methylglutaryl-moiety were not tested. In addition, the statement of the authors, that Cpz27 is controlling the reaction order is not supported by experimental data. Cpz12 and Cpz27 may still be the major resistance mechanism and phosphorylation may occur at different biosynthetic steps directed by the MraY inhibitory activity of the biosynthetic intermediates.

In muraymycin biosynthesis, the transfer of 3-amino-3-carboxypropyl (C4N) onto 3''-O-phospho-ADR-GlyU was recently published. This transfer was shown to be dependent on phosphorylation of the aminoribose. This is in conflict with the data shown in this manuscript, as indicated by the authors. To solve this puzzle, a cpz12 and/or cpz27 knock-out should be presented, otherwise, the conclusions are highly speculative at this time.

Finally, the same is true for Mur23. In this manuscript, plausible data are presented that Mur23 can catalyse a decarboxylation step of compound 11 to generate compound 15 in vitro. Was compound 13 or 14 used with Mur23 as a control? I totally disagree with the statement, that decarboxylation of compound 11 is independent from the presence of the ADR moiety just because of the presence of muraymycin D4 in culture extracts. Muraymycins D4, C4 and A5 could be hydrolysis products as the majority of muraymycins contain the ADR moiety. As long as the exact time point of ADR transfer is not fully established, the presented in vitro data remains speculative at this time, especially because they can differ from in vivo data.

For acceptance of this manuscript for publication, I would recommend submission of a revised manuscript containing:

1. A stronger focus on the nice work on Cpz10, the structural work and the hypothesis, that ADR transfer might occur at a later step in caprazamycin biosynthesis

2. An elimination of the repetitive experiments regarding Cpz14, Cpz15 and Mur17

3. Either provide data of a cpz17 knock-out or be more speculative on the time point of ADR transfer

4. A removal of Cpz12 from the manuscript

5. Provision of data of a cpz27 knock-out or be more speculative in your interpretations

6. Provision of data of Mur23 with compound 13 and 14 or be more speculative in your interpretations

Dear Editor

First of all, I like to thank you for your letter dated Jan. 24, 2022 allowing an extension for revising this manuscript given unexpected personnel change and influences of the pandemics. We are pleased to see generally positive comments from two reviewers. We thank them for their invaluable suggestions as well as insightful points of concern. Now, the manuscript has been revised following your instructions and reviewers' suggestions/comments, where all the concerns are addressed (except some mutants suggested that we were not able to obtain and that the suggested seems a way over the scope of the study) and highlighted in yellow background.

Detailed, point-by-point responses are provided below:

Reviewers' comments:

Reviewer #1: This manuscript reports a biochemical study on two nonheme αKG-dependent enzymes Cpz10 and Cpz15 involved in the biosynthesis of caprazamycin. Unlike the previous assignment of β-hydroxylase, Cpz15 was demonstrated to be the starter enzyme converting uridine mono phosphate to uridine 5'aldehyde, while Cpz10 was found to be the actual β-hydroxylase. In particular, crystal structures together with EPR experiments and NBO calculations tend to support that Cpz10 is an unusual αKG-dependent enzyme with two irons (Fe(III) and Fe(II)). While the active site for O_2 activation was proposed to be Fe(III), the Fe(II) was suggested to be an electron donor for the reduction of Fe(III) site. Such finding seems quite uncommon in αKG-dependent non-hem enzymes. I think the work could be publishable, but the following main issues need to be addressed.

Q 1. In Fig. 4c, it is seen that Fe2 is only coordinated by two waters. I think such coordination environment is unusual in iron-containing proteins. Could it be an unnatural Fe site during crystallographic characterization/analysis?

***R*: As explained in the last paragraph in the section of "Investigation of the nature of Fe^{III} and Fe^{II} EPR signals by NBO analysis", Fe^{II} participates in four CT interactions with Ala132, Ile145, Wt56, and Wt89, resulting in the formation of a 4-coordinate complex inside its binding site as displayed in Figure S20. Therefore, the coordination number of Fe^{II} is four, which corresponds to a tetrahedral molecular geometry. Since we showed only CT interactions of Fe^{II} with Wt56 and Wt89 in Figure 4c, inaccuracy to display CT interactions among Fe^{II}, Ala132, and Ile145 in this figure causes ambiguity in the coordination number of Fe^{II}. We thank reviewer #1 for pointing out this indistinctness; we redrew Figure 4c in which four atoms involved in the CT interactions with Fe^{II} were shown.**

Q 2. According to caption of Figure 6B, EPR was performed with ascorbic acid under an aerobic condition. In such condition, the potential Fe^{II} in the resting state of αKG-dependent enzyme could be oxidized to be Fe^{III} via the formation of $Fe^{III}OO$•— species,

which might disturb the EPR results. So why not do the experiment in the anaerobic condition like reference 63?

***R*: In the reference 63, the author mentioned that they applied nitric oxide (NO) as a prob to detect the signal for Fe^{II} iron in the protein to change the s=2 to s=3/2 then they can detect the iron in the system in anaerobic condition. For example, in isopenicillin N synthase[1], in non-heme Fe complex[2], and in catechol 2,3-dioxygenase and protocatechuate 4,5-dioxygenase[3 and 4]. In this paper (reference 63), not only they ran EPR test in an anaerobic condition but also they ran the test after adding O_2. In this context, if we run the EPR in an anaerobic condition probably we will not see potential Fe^{II}.**

Q 3. For the NBO calculation, Fig S19 has some contradiction with the DFT optimized coordinates in terms of protonation states of His115 and His160. In addition, it looks that neither the structure shown in Fig S19 nor the structure of the DFT coordinates is correct.

***R*: Thank you for raising this very important issue. According to the local electrostatic environment, the imidazole rings of His115 and His160 were protonated at the positions of their Nε2. NBO analysis detects that the Nε2 atoms of both histidines are involved in the CT interactions with Fe1. In Figure S19, we displayed these two CT interactions as lines starting from Fe1 and terminating at the Nε2 atoms in His115 and His160. In fact, we removed the Hε2 atoms bound to these nitrogens in order to increase the clarity of this interaction figure and facilitate finding the involving Nε2 atoms in these CT interactions. Since the lack of Hε2 atoms causes ambiguity in the protonation states of His115 and His160, we maintained the Hε2 atoms attached to the Nε2 atoms by redrawing Figure S19. Therefore, the structure shown in new Figure S19 corresponds to the structure of the DFT coordinates.**

Q 4. Page 6, the fifth line in the second paragraph of Discussion. 'Nonheme αKG-dependent enzymes that contain two irons are not uncommon but often ignored[63, 64, 65]. Indeed, the reference 63, 64 and 65 did not mention the second iron in the enzymes.

***R*: We thank reviewer #1 for pointing out this inconsistence. Reference 63 was an incorrect citation in this manuscript, so we have replaced it with reference 42. In this paper (42), authors clearly mentioned that they can see an extra signal in g-value 2 but cannot explain its phenomenon[6].**

For reference 64, we actually downloaded the pdb and mtz files from the pdb website and refined it. It is clearly that a strong and positive signal shown on the $2F_o$-F_c density map in the active site, which is by no means a water molecule but instead a metal ion likely iron. The authors somehow fitted the density with a water molecule perhaps to save trouble. It is very clear that the atom for the density map is nothing but an iron ion and the distance between two iron ions is 6.04 Å (PDB code: 1uo9)[5] (Figs 1 and 2).

For reference 65, the authors mentioned in the paper that they can observe the second iron in 7.8 Å away from the first iron, whereas they did not provide a clue at the time.

Other small issues:
(1) Page 3, the third line from the bottom in the paragraph before EPR for Cpz10 'This pair of irons may be spatially connected via a hidden electron/charge transfer conduit en route pass points of Wt89, His172, His123, and αKG to Fe1'. Wt89 here should be Wt56 according to Fig. 6A.

***R*. We replaced Wt89 with Wt56 and corrected the pertinent sentence both in the main text and in the caption of Figure 6A as follows:**
"This pair of irons may be spatially connected via a hidden electron/charge transfer conduit en route pass points of Wt56, His172, His123, and αKG to Fe1 or alternatively via Wt89, Arg170, αKG to Fe1 (Figure 6A)."

"A. The hidden CT conduit is schematically presented, where CT routes are proposed from Fe2 en route pass points of Wt56, His172, His123, and αKG to Fe1 or alternatively through Wt89, Arg170, αKG to Fe1."

(2) Page 3, the fourth line in the paragraph Investigation of the nature of Fe^{III} and Fe^{II} EPR signals by NBO analysis 'the valance orbitals containing a total of 6.96 electrons as opposed to the last two orbitals that are Rydberg orbitals with 0.03 electrons'. Here 6.96 should be 6.93.

***R*: The valance orbitals contain a total of 6.93 electrons. Therefore, 6.96 electrons were changed to 6.93 electrons in the revised sentence: "the valance orbitals containing a total of 6.93 electrons as opposed to the last two orbitals that are Rydberg orbitals with 0.03 electrons."**

(3) Page 6, the second line from the bottom in the second paragraph of Discussion. 'On top of that, EPR spectra reflect signal changes for both Fe2 (2.02→1.97 g) and Fe1 (4.75→4.25 g) in the presence or absence of substrates in the Cpz10-mediated reaction'. Values 2.02 and 4.75 in Fig 6B. represent the absence of substrates. So it should be changed to "in the absence or presence of substrates"

***R*: We modified this sentence following reviewer #1 correction: "On top of that, EPR spectra reflect signal changes for both Fe2 (2.02 → 1.97 g) and Fe1 (4.75 → 4.25 g) in the absence or presence of substrates in the Cpz10-mediated reaction."**

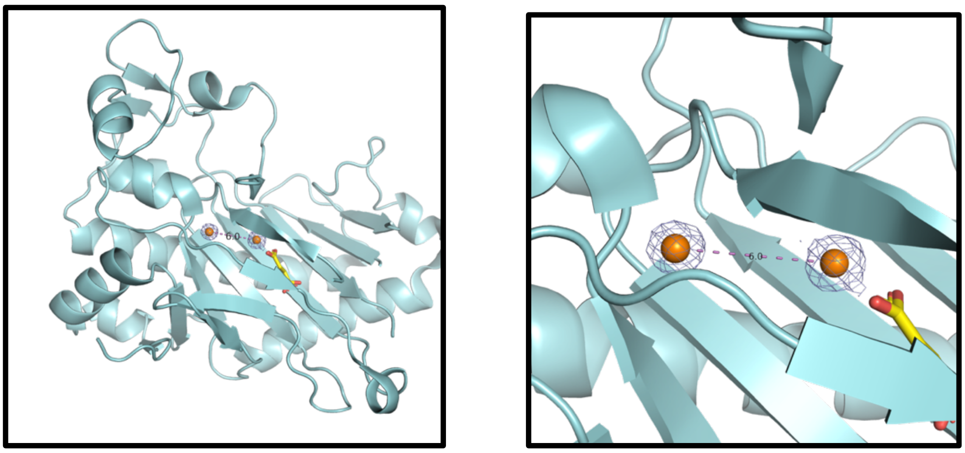

Figure 1. The density map and distance measurements of two iron ions after refinement.

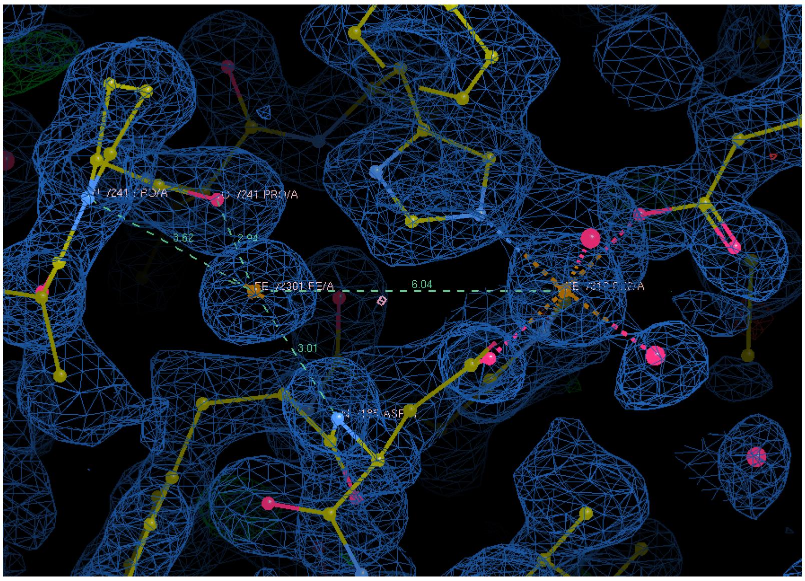

Figure 2. Illustration of the 1uo9 structure by pymol and the density map of two irons in 6 Å distance.

References

1. Chen, V. J., et al. "Spectroscopic studies of isopenicillin N synthase: a mononuclear nonheme Fe2+ oxidase with metal coordination sites for small molecules and substrate." *Journal of Biological Chemistry* 264.36 (1989): 21677-21681.
2. Chiou, Yu-Min, and Lawrence Que Jr. "Model Studies of. alpha.-Keto Acid-Dependent Nonheme Iron Enzymes: Nitric Oxide Adducts of [FeII (L)(O2CCOPh)](ClO4) Complexes." *Inorganic Chemistry* 34.12 (1995): 3270-3278.
3. Arciero, David M., and J. D. Lipscomb. "Binding of 17O-labeled substrate and inhibitors to protocatechuate 4, 5-dioxygenase-nitrosyl complex. Evidence for direct substrate binding to the active site Fe2+ of extradiol dioxygenases." *Journal of Biological Chemistry* 261.5 (1986): 2170-2178.
4. Arciero, D. M., A. M. Orville, and J. D. Lipscomb. "[17O] Water and nitric oxide binding by protocatechuate 4, 5-dioxygenase and catechol 2, 3-dioxygenase. Evidence for binding of

exogenous ligands to the active site Fe2+ of extradiol dioxygenases." *Journal of Biological Chemistry* 260.26 (1985): 14035-14044.
5. Valegård, Karin, et al. "The structural basis of cephalosporin formation in a mononuclear ferrous enzyme." *Nature structural & molecular biology* 11.1 (2004): 95-101
6. Wolfe, M. D. & Lipscomb, J. D. Hydrogen peroxide-coupled cis-diol formation catalyzed by naphthalene 1, 2-dioxygenase. *J. Biol. Chem.* **278**, 829-835 (2003).

Reviewer #2

The Manuscript "ß-Hydroxylation for α-amino-β-hydroxylbutanoyl-glycyluridine catalysed by a nonheme hydroxylase warrants reaction fidelity toward maturation of caprazamycin" reported the functions of selected enzymes in caprazamycin biosynthesis. The manuscript focuses on the non-heme α-ketoglutarate-dependent ß-hydroxylase Cpz10 which is responsible for ß-hydroxylation of the 3-amino-3-carboxypropyl side chain of a caprazamycin intermediate. Reviewer highlighted that the ß-hydroxylation prior to diazepanone ring formation has been an open question for a long time in liponucleoside research and therefore is of great interest for the natural product community.

Q 1. *In general, this manuscript is lacking a focus on the biochemical characterization of* Cpz10. *Instead, preliminary work on other enzymes such as phosphotransferases and the PLP-dependent decarboxylase* Mur23 *are included, deflecting from the major story and conflicting even with previous results.*

R. **We thank reviewer #2 for raising these two concerns, deflecting from the major story and conflicting even with previous results. While that is one way to think about it, we beg to differ. The facts and conclusions that we added and put forward in this manuscript are faithful to experimental data regardless of previous results (particularly the synthetic compound 11 that can be decarboxylated by Mur23 to yield compound 15). As we pointed out in the main text, the inconsistence is worth having comprehensive investigation for those who are interested/concerned in the field in the future. Based on new findings, we come up with a scheme that compound 11 stands at a watershed, where its β-hydroxylation permits the seven-membered diazepanone ring cyclization and acylation toward a fully-active caprazamycin; it, otherwise, undergoes decarboxylation by Mur23 exemplified here toward the formation of muraymycin. We hightlight that the role of Mur23 is part of a united whole for the story integrity but not a hodge-podge of ideas.**

Q 2. *In the first section of the results chapter, the authors claimed that a controversy has to be resolved since* Cpz10 *and* Cpz15 *both could catalyse the first step in liponucleoside biosynthesis, that is the oxidative dephosphorylation of* UMP. *However, the van Lanen group has solved this controversy ten years ago by studying the corresponding enzyme* LipL *from liposidomycin biosynthesis.*

R. **We thank reviewer #2 for lending a view on this subject from historical**

perspective. Despite high similarity between liposidomycin and caprazamycin, our study was focused mainly on Cpz10 that nonetheless can be mistaken for Cpz15 at sequence level (both code for a αKG dependent enzyme) concerning the caprazamycin biosynthesis. As a matter of fact, Cpz15 per se was not biochemically characterized. In the biosynthesis of α-amino-β-hydroxylbutanoyl-glycyluridine, the "open question" of Cpz10 was characterized as being the β-hydroxylase, whereas Cpz15 is the enzyme committing the initial step a critical sideline of the main story. On top of that, both the structure of compound 13 (purified from the culture of ΔCpz10) and the result of Cpz10 versus compound 11 (chemically synthesized) are in vivo and in vitro finale to conclude the story.

Q 3. *The same occurs for* Cpz14. *Formation of (5'S,6'S)-5'-C-glycyluridine has been shown to be catalyzed by* LipK. *The new aspect of this manuscript is that* Mur17 *seems to do the same reaction but does not only rely on threonine as the only aldol donor, but also accepts glycine. Unfortunately, the corresponding ESI-MS data are not presented. Supplemental figure 5 indicates in the legend for c, (i) control (ii)* Mur17 + *PLP + threonine and (iii)* Mur17 + *PLP + glycine but c does neither show (i), (ii) or (iii). Only the ESI-MS of compound 10 is shown. Fig 3B only shows the corresponding LC traces.*

R. **We thank reviewer #2 for indicating some insufficiency. We agree that this study did not cover everything for Cpz14. As we mentioned in the manuscript, given that Cpz14 is insoluble we were unable to perform biochemical analysis. For the study to proceed, we borrowed Mur17 from the muraymycin biosynthesis, as Mur17 that executes the same reaction was known. Following the reviewer's comment, the extra legend was removed from Supplemental figure 5. The LC traces for reactions of (i) control (ii) Mur17 + threonine and (iii) Mur17 + glycine are shown in Fig 3B in the main text.**

Q 4. *In the second chapter of the results (*Cpz10 *is the ß-hydroxylase), the authors claimed that "it has been proposed that the four-carbon sidechain is transferred from* SAM *to compound 10 by* Cpz13*…" This statement is not entirely true. Compound 10 lacks the aminoribose (*ADR*), the actual proposal is that prior to the attachment of* C4N*, the disaccharide core structure (ADR-*GlyU*) is formed by the action of* Cpz17 *(as it has been demonstrated for* LipN*). With the synthetic compound 11 in hand (reference 14 for the synthesis is wrong), the authors can clearly demonstrate, that the hydroxylated compound 11 is formed, generating compound 12. <u>This is an interesting finding since compound 12 lacks the ADR moiety</u>. This opens the general question, at which time point* ADR *is attached. Interestingly, a cpz10 deletion mutant only accumulated the* ADR-GlyU *disaccharide (compound 13). This can only be explained if the ribosyltransferase* Cpz17 *can transfer* ADR *onto both, compound 11 and 12. Otherwise, the presented results are not only in disagreement with the biochemical studies on the* Cpz17 *homologue,* LipN*, but also with their own findings of the cpz10 knock-out mutants. To clarify the situation, one should consider a cpz17 knock-out. If compound 11 and 12 are accumulated in this mutant, this would be a strong indication for the proposed pathway in Supplementary Scheme 3. In addition, the authors do not explain, why the C7' carboxyl group is methylated. Methylation of the C6' amine could*

*be explained by the action of one of the N-methyltransferases (*Cpz11 *or* 26*) prior ß-hydroxylation. In Figure 2, compound 13 and 14 contain a hydroxyl-group at C5'' of the* ADR *moiety (Fig 2B) but they contain the amino group in Fig 2D. I assume that the structure in Fig 2B is wrong.*

***R.* To make the reply simple, we follow the order of last in first out.**

We thank reviewer #2 for pointing out two mistakes in Fig 2B, which have now been fixed, that is, OH at C5" is changed to NH_2.

*"why the C7' carboxyl group is methylated. Methylation of the C6' amine could be explained by the action of one of the N-methyltransferases (*Cpz11 *or* 26*) prior ß-hydroxylation.":* **Because we were afraid to deflect from the major story, we did not elaborate the methylation at the C7′ carboxyl group. Our reasoning is that both the C6' amine and the C7' aminde group of the diazepanone ring is methylated by the action of the same N-methyltransferase (Cpz11 or 26) while the methylation at the C7' amide group takes place after the ring closure (envisioning that both secondary a-amines have a similar spatial geometry and the ring is rotatable at C5' for molecular recognition by the same enzyme). The C7' carboxyl group, on the other hand, is methylated by the action of the O-methyltransferase (the remaining Cpz11 or 26), of which the resulting methoxyl group turns out to be a good leaving group for the subsequent ring cyclization to take place. This process differs from the canonical amino acid adenylation by reacting with ATP and the b-hydroxyl group appears to be critical for the ring to cyclize.**

"*With the synthetic compound 11 in hand (reference 14 for the synthesis is wrong), the authors can clearly demonstrate, that the hydroxylated compound 11 is formed, generating compound 12. This is an interesting finding since compound 12 lacks the ADR moiety. This opens the general question, at which time point* ADR *is attached. The cpz10 deletion mutant only accumulated the* ADR-GlyU *disaccharide (compound 13), suggesting that the ribosyltransferase* Cpz17 *can transfer* ADR *onto both, compound 11 and 12. To clarify the situation, one should consider a cpz17 knock-out. If compound 11 and 12 are accumulated in this mutant, this would be a strong indication for the proposed pathway in Supplementary Scheme 3.":* **As a matter of fact, we have been trying to make a *cpz17* knock-out ever since the biosynthetic scheme proposed as well as during this revision time period, whereas the goal is unfortunately yet to reach. Nonetheless, we like to restate that the proposed biosynthetic scheme was based on our experimental findings out of Cpz10, Mur23, etc., where Cpz10 can only accept compound 11 rather than compound 13 and Mur23 cannot decarboxylate compound 13. The structure of compound 13 serves as the foundation to support the proposed biosynthetic scheme, where ribosyltransferase Cpz17 is very likely to take on compound 12. We agree that this remains an open question worth furthering study and publishing the results in a separate paper.**

Q 5. *The last chapter on phosphotransferase activity offers some preliminary results again contradicting previous findings. First,* Cpz12 *is mentioned in the abstract as an*

enzyme, which was characterized in this study. Besides the successful expression and purification of the phosphotransferase, no data are presented, thereby Cpz12 *should be deleted from this manuscript.*

R. **We thank reviewer #2 for this suggestion. By taking this suggestion, we have removed Cpz12 from the abstract. To determine either Cpz12 or Cpz27 the committed kinase that phosphorylate compound 13. We performed enzymatic assays for both enzymes, whereby Cpz12 was ruled out taking part in the phosphorylation. By taking the reviewer's suggestion, we downplayed Cpz12 (only serves as the contrast to Cpz27) but preserved Cpz27.**

Q 6. *Secondly, the authors could demonstrate, that* Cpz27 *can phosphorylate compound 13, but not caprazol or caprazamycins. This is not an indication, that phosphorylation occurs before diazepanone ring formation as stated by the authors since aglycones and caprazamycins lacking the 3-methylglutaryl-moiety were not tested. In addition, the statement of the authors, that* Cpz27 *is controlling the reaction order is not supported by experimental data.* Cpz12 *and* Cpz27 *may still be the major resistance mechanism and phosphorylation may occur at different biosynthetic steps directed by the* MraY *inhibitory activity of the biosynthetic intermediates.*

R. **This is likewise an open question. Cpz12 and Cpz27 that may be the major resistance mechanism cannot be ruled out at this stage. As to that phosphorylation occurs at different biosynthetic steps directed by the MraY inhibitory activity of the biosynthetic intermediates is somewhat speculative given no supportive evidence. One should realize that neither compound 13/14 nor other intermediates were ever reported possessing antimicrobial activity or showing MraY binding affinity. In literatures, a paper recently published in NCB 2020 by Prof. Yokoyama et al.[1] proposed that Cpz27 might be an enzymatic tag or engage in a self-resistance mechanism. The fact that the phosphorylation of compound 13 does take place before diazepanone ring cyclization in support of the proposition that Cpz27 is a phosphotransferase controlling the reaction order, which is upheld by another paper published by Prof. Kuzuyzmz et al.[2]**

Q 7. *In muraymycin biosynthesis, the transfer of 3-amino-3-carboxypropyl (C4N) onto 3"-O-phospho-ADR-GlyU was recently published. This transfer was shown to be dependent on phosphorylation of the aminoribose. This is in conflict with the data shown in this manuscript, as indicated by the authors. To solve this puzzle, a cpz12 and/or cpz27 knock-out should be presented, otherwise, the conclusions are highly speculative at this time.*

R. **In muraymycin biosynthesis, the transfer of 3-amino-3-carboxypropyl (C4N) onto 3"-O-phospho-ADR-GlyU was recently published, which contradicts to the previously proposed scheme where the phosphorylation takes place after the transfer of 3-amino-3-carboxypropyl (C4N) onto the disaccharide. In caprazamycin biosynthesis, Cpz27 acts on compound 13 to bring on compound 14, which is likewise in conflict with previous results/hypothesis. One should feel no**

surprise given conflicts in results and viewpoints. A *cpz12* and/or *cpz27* knock-out may be conducive to solving this puzzle, whereas this bunch of new results should be published in a separate paper with in-depth elaboration. Here, we focused mainly on Cpz10 and Mur23 that each hydroxylates and decarboxylates chemically synthesized compound 11, the missing or unresolved parts from previous studies in caprazamycin biosynthesis [3 and 4].

Q 8*. Finally, the same is true for* Mur23*. In this manuscript, plausible data are presented that* Mur23 *can catalyse a decarboxylation step of compound 11 to generate compound* 15 *in vitro. Was compound* 13 *or* 14 *used with* Mur23 *as a control? I totally disagree with the statement, that decarboxylation of compound* 11 *is independent from the presence of the ADR moiety just because of the presence of muraymycin* D4 *in culture extracts. Muraymycins* D4, C4 *and* A5 *could be hydrolysis products as the majority of muraymycins contain the ADR moiety. As long as the exact time point of ADR transfer is not fully established, the presented in vitro data remains speculative at this time, especially because they can differ from in vivo data.*

***R*. This is another view of conflict. We understand that scientific evidence supports a view of conflict (as a consequence of in vivo or in vitro experiments) that may actually incorporate multiple properties. Aside from this, we have run the reactions for Mur23 in the presence of compound 13 and the reaction of Mur23 in conjunction with Cpz27 against compound 14, whichever the condition there is no new peak emerged suggesting compounds 13 and 14 are not the substrates of Mur23.**

Q 9. A stronger focus on the nice work on Cpz10*, the structural work and the hypothesis, that ADR transfer might occur at a later step in caprazamycin biosynthesis*

***R.* We thank the reviewer for the comment. That indeed is the centerpiece of the work, where Cpz10 plays a pivotal role that was consolidated by upstream and downstream enzymes in the caprazamycin biosynthetic pathway as well as those from other analogs. The study of this enzyme was pursued though multiple approaches including biochemistry, mutation, biophysics, computation, and synthetic chemistry in order to reveal new findings, resolve puzzles, and raise new questions. The new issues should deserve new efforts to integrate all relevant information as a whole in the future.**

Q 10. An elimination of the repetitive experiments regarding Cpz14*,* Cpz15 *and* Mur17

***R.* Many thanks for the suggestions. The inclusion of these enzymes, minor but cohesive/critical, is meant to establish and conclude the role of Cpz10 in the pathway. Mur17 turns out to be a special case (an equivalent to Cpz14 that is insoluble in this study), as it not only accepts threonine but also glycine, which was not reported in the paper published by Prof. van Lanen[5]. Both Cpz15 and Cpz10 belong to the family of αKG dependent enzymes. Though at gene level Cpz15 is**

equivalent to Mur16, Cpz15 has not been biochemically determined. It, clearly, remained an open issue until the determination in the current work.

Q 11. Either provide data of a cpz17 knock-out or be more speculative on the time point of ADR transfer

R. **Please refer to the reply the third paragraph for question 4.**

Q 12. A removal of Cpz12 *from the manuscript*

R. **Please refer to the reply for question 5. Simplify speaking, Cpz12 has been removed from the abstract and briefly mentioned in the text.**

Q 13. Provision of data of a cpz27 knock-out or be more speculative in your interpretations

R. **Please refer to the reply for question 7.**

Q 14. Provision of data of Mur23 *with compound* 13 *and* 14 *or be more speculative in your interpretations*

R. **Please refer to the reply for question 8.**

References

1. Draelos, Matthew M., et al. "Cryptic phosphorylation in nucleoside natural product biosynthesis." *Nature chemical biology* 17.2 (2021): 213-221.
2. Shiraishi, Taro, et al. "Biosynthesis of the antituberculous agent caprazamycin: Identification of caprazol-3″-phosphate, an unprecedented caprazamycin-related metabolite." *The Journal of general and applied microbiology* 62.3 (2016): 164-166.
3. Cui, Zheng, et al. "Pyridoxal-5′-phosphate-dependent alkyl transfer in nucleoside antibiotic biosynthesis." *Nature chemical biology* 16.8 (2020): 904-911.
4. Cui, Zheng, et al. "Self-resistance during muraymycin biosynthesis: a complementary nucleotidyltransferase and phosphotransferase with identical modification sites and distinct temporal order." *Antimicrobial agents and chemotherapy* 62.7 (2018): e00193-18.
5. Cui, Zheng, et al. "Enzymatic synthesis of the ribosylated glycyl-uridine disaccharide core of peptidyl nucleoside antibiotics." *The Journal of organic chemistry* 83.13 (2018): 7239-7249.

We, once again, thank both reviewers for their thorough and comprehensive review and valuable comments. Please let me know if you need additional information. Thank you for your consideration of the manuscript.

Sincerely yours

Tsung-Lin, Li

REVIEWERS' COMMENTS:

Reviewer #1 (Remarks to the Author):

My questions have been addressed. I think the manuscript can be published

Reviewer #2 (Remarks to the Author):

The Manuscript "β-Hydroxylation for α-amino-β-hydroxylbutanoyl-glycyluridine catalysed by a nonheme hydroxylase warrants reaction fidelity toward maturation of caprazamycin" has been revised. All the major and minor issues were addressed satisfactory. The work indeed add some very interesting insights into biosynthetic steps and possible reaction orders within caprazamycin biosynthesis. The revised version of the manuscript now contains a more careful phrasing concerning previous findings and is now ready for publication.
